# Lanthanide(III) Complexes of Cyclen Triacetates and Triamides Bearing Tertiary Amide-Linked Antennae

**DOI:** 10.3390/molecules25225282

**Published:** 2020-11-12

**Authors:** Salauat R. Kiraev, Emilie Mathieu, Fiona Siemens, Daniel Kovacs, Ellen Demeyere, K. Eszter Borbas

**Affiliations:** Department of Chemistry, Ångström Laboratory, Uppsala University, Box 523, 75120 Uppsala, Sweden; salauat.kiraev@kemi.uu.se (S.R.K.); emilie.mathieu@lcc-toulouse.fr (E.M.); fiona.siemens@googlemail.com (F.S.); daniel.kovacs1990@gmail.com (D.K.); Ellen.Demeyere@UGent.be (E.D.)

**Keywords:** lanthanide, luminescence, DO3A, coumarin, carbostyril, electrochemistry

## Abstract

The coordination compounds of the trivalent lanthanide ions (Ln(III)) have unique photophysical properties. Ln(III) excitation is usually performed through a light-harvesting antenna. To enable Ln(III)-based emitters to reach their full potential, an understanding of how complex structure affects sensitization and quenching processes is necessary. Here, the role of the linker between the antenna and the metal binding fragment was studied. Four macrocyclic ligands carrying coumarin 2 or 4-methoxymethylcarbostyril sensitizing antennae linked to an octadentate macrocyclic ligand binding site were synthesized. Complexation with Ln(III) (Ln = La, Sm, Eu, Gd, Tb, Yb and Lu) yielded species with overall −1, 0, or +2 and +3-charge. Paramagnetic ^1^H NMR spectroscopy indicated subtle differences between the coumarin- and carbostyril-carrying Eu(III) and Yb(III) complexes. Cyclic voltammetry showed that the effect of the linker on the Eu(III)/Eu(II) apparent reduction potential was dependent on the electronic properties of the N-substituent. The Eu(III), Tb(III) and Sm(III) complexes were all luminescent. Coumarin-sensitized complexes were poorly emissive; photoinduced electron transfer was not a major quenching pathway in these species. These results show that seemingly similar emitters can undergo very different photophysical processes, and highlight the crucial role the linker can play.

## 1. Introduction

The luminescence of the coordination compounds of lanthanide ions (Ln(III)) has found application in a variety of fields, such as in biological sensing and imaging [1], in fluorescent lamps and lasers [2], and in anti-counterfeiting [3,4,5]. The direct excitation of Ln(III) is inefficient due to the low absorption coefficients of the Laporte-forbidden 4f-4f transitions [6]. A common method for overcoming the challenge of low Ln(III) absorptions is to excite the metal ion through a light-harvesting chromophore, a so-called antenna [6,7]. The antenna can transfer the excitation energy to the Ln(III), thus combining the attractive Ln(III) luminescence properties (sharp, spiked emission peaks, long excited-state lifetimes) with the strong absorption of common organic chromophores. The presence of the antenna offers additional benefits. It can carry reactive groups for attachment to biomolecules [8], or labels (e.g., F-19) [9] for creating multimodal imaging agents. The antenna can also provide a way to render the Ln(III) complex analyte-responsive [10,11,12,13,14].

**Figure 1:**
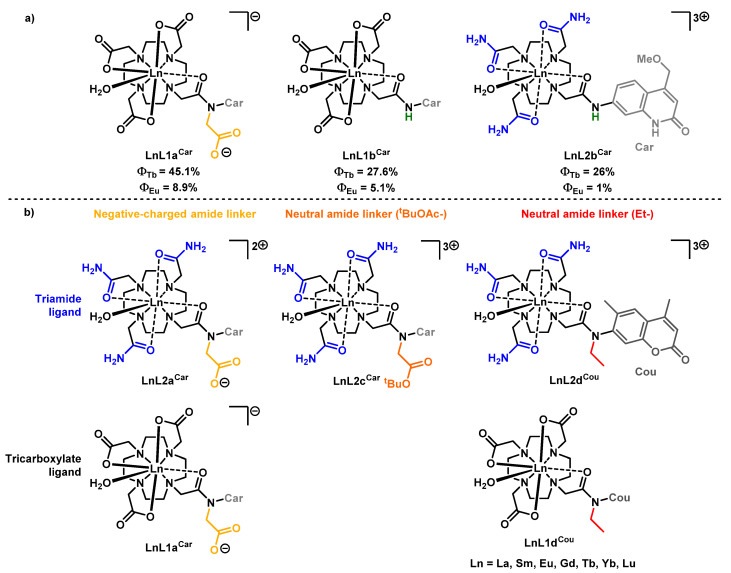
(**a**) Comparison of the Eu(III) and Tb(III) luminescence quantum yields of secondary and tertiary amide-linked carbostyril-sensitized Ln(III) complexes. (**b**) Complexes studied here.

Given the importance of the energy transfer for Ln(III) sensitization, it is not surprising to see the amount of research dedicated to the optimization of the antenna photophysical properties. Helpful guidelines are available for the matching of Ln(III) with the antenna, which discuss the antenna single-triplet gap, and the energy gap between the antenna triplet and the Ln(III) receiving levels [15]. Both the antenna and the lanthanide excited states are susceptible to quenching, which needs to be minimized if highly emissive complexes are to be obtained. Ln(III) have 8–9 coordination sites, most of which need to be occupied by a multidentate ligand for the complex to be kinetically and thermodynamically stable [16]. Furthermore, X-H (X = O, N, C) oscillators quench Ln(III) excited states [17,18], and should thus be eliminated if possible. The complexes shown in Figure 1 have only one inner-sphere water molecule.

Recently, we reported that Ln(III) complexes sensitized by carbostyril antennae that are attached to the metal binding site via tertiary amide linkers are substantially more luminescent than the analogous secondary amide-linked ones (Figure 1a) [19]. While the amide linker certainly influences the antenna excited states, the effect is rather subtle. Furthermore, an X-ray crystal structure of a precursor of the tertiary amide-linked complexes indicated that the orientation of the antenna in the two types of emitters may be different, which could have a substantial effect on the energy transfer efficiency.

Possible structural effects that explain the differences observed between the photophysical properties of secondary and tertiary amide-linked complexes are investigated in this work. We studied the effect of charge on the amide linker substituent, and whether the effect is carried over to complexes carrying a coumarin antenna or a ligand binding site wherein the three carboxylate donors were replaced by three amides (Figure 1b).

## 2. Results

### 2.1. Synthesis

**Scheme 1:**
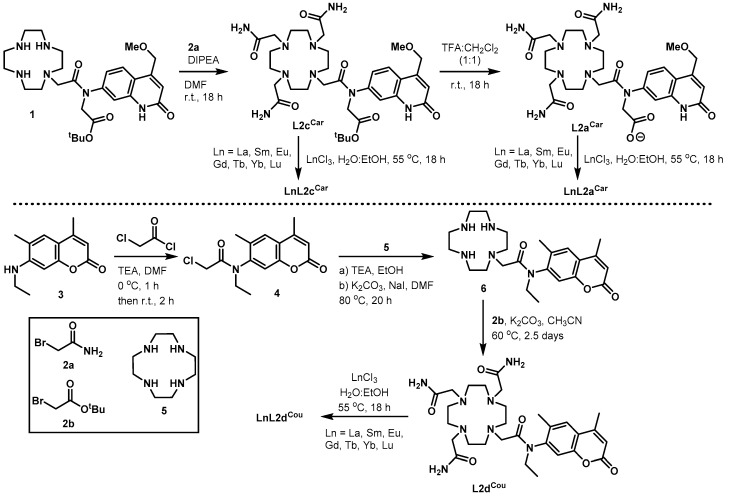
Synthesis of new ligands and complexes.

The syntheses of **LnL1a^Car^** and **LnL1d^Cou^** have been reported previously, except their La, Sm, Yb and Lu analogues [19,20,21]. Triamides **LnL2a^Car^**, **LnL2c^Car^** and **LnL2d^Cou^** were synthesized following analogous procedures, these are summarized in Scheme 1. Detailed synthetic protocols and complete characterization of all new compounds are given in the Materials and methods. Syntheses started from known monoalkylated cyclens **1 [22]** or **6 [20]**. Trialkylation with bromoacetamide was carried out in the presence of diisopropyl ethylamine (DIPEA) or K_2_CO_3_ to yield **L2c^Car^** or **L2d^Cou^**, respectively. Heating the latter at 55 °C in an aqueous-ethanolic mixture with a slight excess of LnCl_3_ afforded **LnL2d^Cou^** (Ln = La, Sm, Eu, Gd, Tb, Yb and Lu) in high yield. Stirring **L2c^Car^** in a 1:1 mixture of CH_2_Cl_2_ and trifluoroacetic acid (TFA) overnight cleaved the *tert*-butyl ester in the tertiary amide side chain to give **L2a^Car^**, which was then used to form the complexes **LnL2a^Car^** analogously to **LnL2d^Cou^**. The reaction mixtures of **LnL2a^Car^** had to be purified via semipreparative HPLC to afford analytically pure samples, as the complexes partially decomposed during synthesis. We attempted to synthesize the *tert*-butyl-protected **LnL2c^Car^** the same way as the other **LnL2** triamide species. The ^t^Bu ester in the Ln(III) complexes was labile, and hydrolyzed quantitatively to **LnL2a^Car^**. This was confirmed by LC-MS analysis of their reaction mixtures stirred overnight which showed the presence of both **LnL2a^Car^** and **LnL2c^Car^** species in a 1:2 ratio (Figures S1–S3). The ^1^H spectra of **LaL2c^Car^** and **LuL2c^Car^** confirmed the presence of both hydrolyzed and ^t^Bu ester-protected species in 1:0.45 and 1:0.25 ratios, respectively (Figures S4 and S5).

### 2.2. Paramagnetic ^1^H NMR Spectroscopy

**Figure 2:**
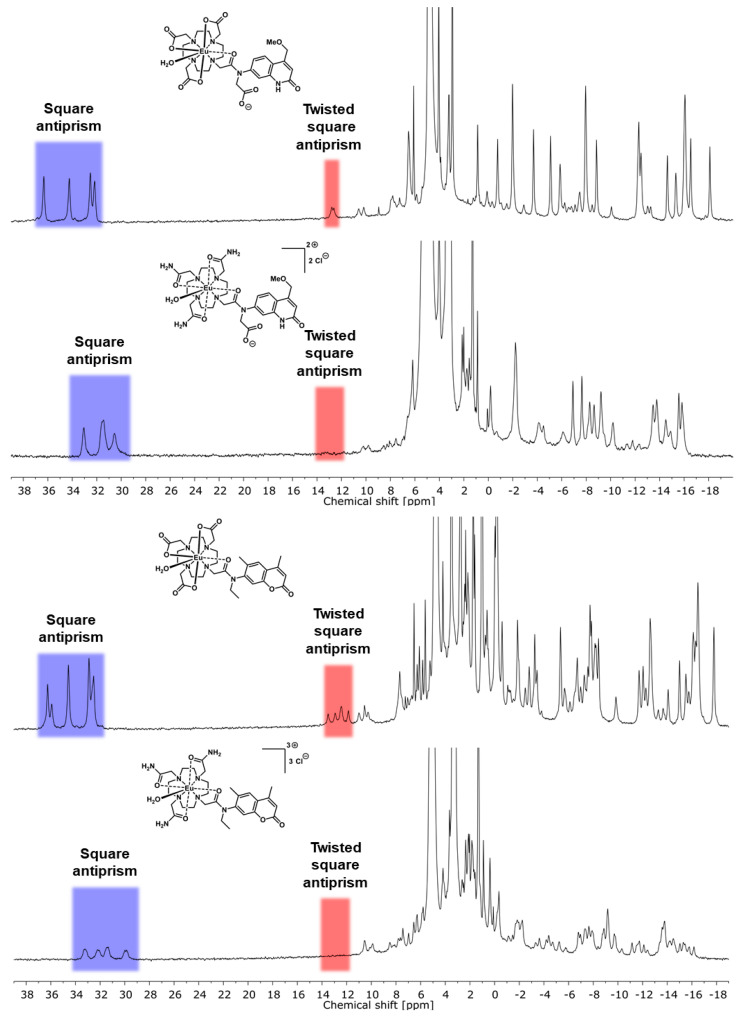
Stacked ^1^H NMR spectra (400 MHz) of Eu complexes measured in D_2_O (**EuL1a^Car^** and **EuL1d^Cou^**) at 10 °C and CD_3_OD (**EuL2a^Car^** and **EuL2d^Cou^**) at 0 °C with the regions corresponding to TSAP and SAP cyclen ring protons in red and blue, respectively.

**Figure 3:**
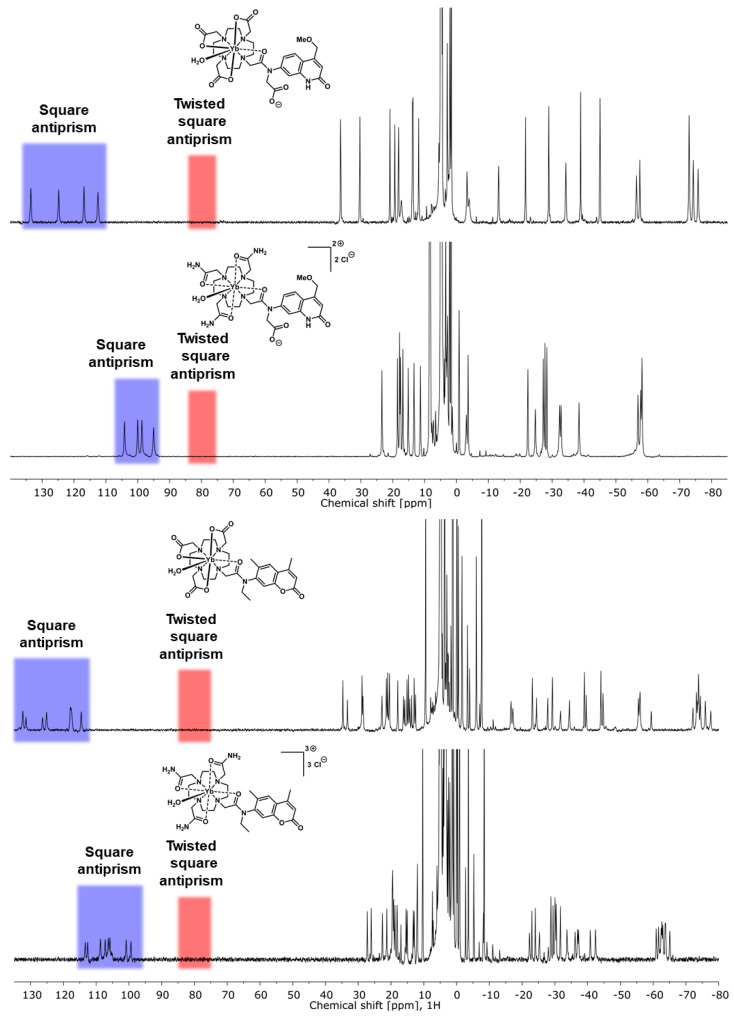
Stacked ^1^H NMR spectra (400 MHz) of Yb complexes measured at r.t. in D_2_O (**YbL1a^Car^**, **YbL1d^Cou^** and **YbL2a^Car^**) and CD_3_OD (**YbL2d^Cou^**) with the regions corresponding to TSAP and SAP cyclen ring protons in red and blue, respectively.

Cyclen-based DO3A-type Ln complexes interconvert between several structures in solution [23]. This dynamic process was studied by paramagnetic ^1^H NMR spectroscopy of the Eu(III) and Yb(III) chelates (Figure 2 and Figure 3). The spectra of the diamagnetic La(III) and Lu(III) complexes were similar to those of the ligands (Figures S6–S13). Eu(III) and Yb(III) paramagnetic centers caused broadening and relocation of the signals due to the exchange between square antiprism (SAP) and twisted SAP (TSAP) isomers (Figures S14–S25). The resonances belonging to SAP and TSAP isomers can be distinguished from their different signals of axial cyclen ring protons [24]. Well-defined signals could be obtained upon cooling down the solutions of the Eu(III) complexes [25]. Tricarboxylate complexes **EuL1a^Car^** and **EuL1d^Cou^** were present as mixtures of TSAP (12.6–12.8 ppm and 11.9–13.5 ppm) and SAP (32.2–36.3 ppm and 32.5–36.3 ppm) conformers in D_2_O at 10 °C (Figure 2). The ratios of the signals attributed to the SAP and TSAP species were 1:0.22 and 1:0.34 for **EuL1a^Car^** and **EuL1d^Cou^**, respectively (Figures S14 and S18). The spectra of triamide **EuL2a^Car^**, **EuL2c^Car^** and **EuL2d^Cou^** complexes in CD_3_OD at 0 °C contained only signals from the SAP isomer (30.6–33.1, 29.9–32.8 and 29.9–33.2 ppm, respectively), as did all the Yb(III) complexes at r.t. (**YbL1**: 112.5–133.5 ppm (D_2_O), **YbL2**: 95.0–113.3 ppm (D_2_O and CD_3_OD), Figure 3).

The most downfield signals are assigned to the pseudo-axial cyclen ring protons in the SAP conformers [26,27]. In the unsymmetrical complexes discussed here, up to four signals are expected. This is the case in the carbostyril complexes, where the ^1^H NMR spectra of both the Eu and Yb species have four distinct peaks in the 32–36/30–33 ppm and 112–133/95–113 ppm (tricarboxylate/triamide) regions, respectively. However, the spectra of the coumarin-appended complexes present more than four well-resolved signals in most cases, indicating the presence of more than one complex with SAP conformation (Figure 2 and Figure 3, bottom). A possible reason for this observation may be restricted rotation of the antenna due to steric clash between the 6-Me and amide C=O groups.

### 2.3. Cyclic Voltammetry

One of the processes that can quench the excited antenna is photoinduced electron transfer (PeT) to the Ln(III). PeT is often thermodynamically feasible for Eu and Yb, but not for Gd and Tb, as the latter two have very negative reduction potentials (E(Gd^III^/Gd^II^) = −3.9 V and E(Tb^III^/Tb^II^) = −3.7 V vs NHE, respectively [28]). The driving force for PeT is larger for Ln(III) with more positive reduction potentials [29]. There is ample evidence that the reduction potential of the Ln(III) depends on the ligand [30,31,32,33,34,35]. The replacement of neutral amide donors with −1 charged carboxylates in DO3A-type ligands has been found to shift the Eu(III) reduction potential to more negative values by ~95 mV/donor [36]. We expected that more subtle tuning may be achieved by the manipulation of the antenna linker. This hypothesis was investigated by cyclic voltammetry, the results are summarized in Table 1.

Similarly to what was observed previously [36], the replacement of tricarboxylate by triamide ligand in coumarin complexes increases the Eu(III)/Eu(II) reduction potential from −854 mV vs NHE in **EuL1d^Cou^** to −565 mV vs NHE in **EuL2d^Cou^**, which corresponds to ~96 mV/donor (Table 1).

**Figure 4:**
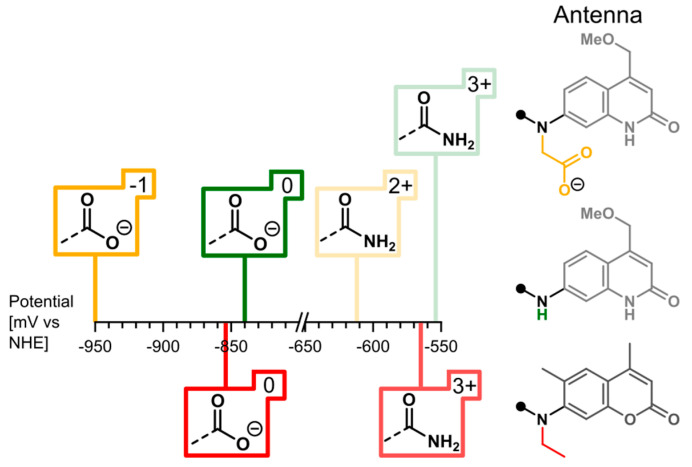
Coordination environment vs apparent Eu(III)/Eu(II) reduction potential of **EuL** complexes. **EuL1-2a^Car^** are in dark yellow, **EuL1-2b^Car^** are in dark green and **EuL1-2d^Cou^** are in red.

The introduction of a negatively-charged amide linker in **EuL1a^Car^** and **EuL2a^Car^** leads to a 109 mV and 58 mV decrease in the apparent Eu(III)/Eu(II) reduction potentials, respectively, compared to **EuL1b^Car^** and **EuL2b^Car^** with secondary amide-linked antennae (Figure 4, Table S3). The shift is most likely due to the additional negative charge on the linker, as the reduction potentials of **EuL2b^Car^** and the ^t^Bu-protected ester-carrying **EuL2c^Car^** are almost identical (−554 mV vs −564 mV vs NHE, respectively). The apparent reduction potential of **EuL1d^Cou^** is only 15 mV lower than the secondary amide-linked **EuL1b^Car^**. Hence, the influence of the neutral amide linker (^t^Bu-protected methylcarboxylate or Et) on the reduction potential is small (Figure S26), whereas the negatively charged arm leads to a better stabilization of Eu(III). It is possible that the replacement of the carbostyril heterocycle with a coumarin 2 also has a slight influence. Either way, the effects are small (Figure S27) and are in the measurement error range (~10 mV at a scan rate 100 mV/s [36]).

From the above data we can conclude that the electronic impact of the amide linker in **EuL1a^Car^** can be ascribed to its negative charge, which contributes to the stabilization of the +3 oxidation state.

### 2.4. Photophysical Characterization

**Figure 5:**
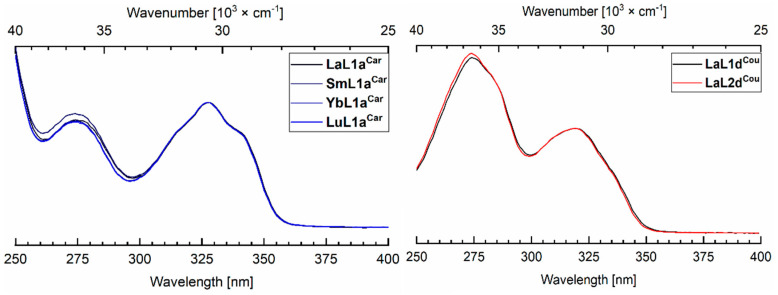
Superimposed UV-Vis absorption spectra of **LnL1a^Car^** (Ln = La, Sm, Yb, Lu) (left) and **LaL1d^Cou^** and **LaL2d^Cou^** (right) complexes normalized at 328 nm and 319 nm, respectively. [**LnL**] = 10 µM and was measured in aqueous 10 mM PIPES buffer at pH 6.5.

The UV-Vis absorption and emission properties of the complexes were studied in 10 mM PIPES buffer in H_2_O or D_2_O (pH or pD 6.5) at r.t. at nominally 10 µM concentrations. The new carbostyril-carrying complexes had UV-Vis absorption spectra that resembled those of the previously reported **LnL1a^Car^**. Only small variations were seen between complexes of the same ligand with different Ln(III) ions (Figure 5, left), and the complexes had local absorption maxima at *λ*_abs_ = 328 nm. Replacing the tricarboxylate metal-binding framework with a triamide one essentially did not impact the complex absorptions (Figure S42). Coumarin-appended **LnL1d^Cou^** and **LnL2d^Cou^** similarly had superimposable absorption spectra (Figure 5, right), with *λ*_abs_ = 319 nm (Figure S43). 

The triplet levels of the carbostyril and coumarin antennae were determined from the steady-state emission spectra of the Gd(III) complexes at 77 K upon excitation at *λ*_ex_ = 327 and 315 nm, respectively (Figure S44). The triplet excited state (T_1_) of carbostyril was located at 23,000 cm^−1^, while that of the coumarin was found at slightly lower energies, and 22,200 cm^−1^. These values were determined from the 0-0 phonon transitions. The energies of both types of antennae were expected to be suitable for sensitization of Tb(III), Eu(III) and Sm(III), with excited states at 20,600 (^5^D_4_), 17,300 (^5^D_0_) and 18,000 (^4^G_5/2_) cm^−1^ [37], respectively. As the coumarin T_1_ is within ~2000 cm^−1^ of the Tb(III) excited state, back energy transfer from Tb(III) to T_1_ was considered a possibility.

**Figure 6:**
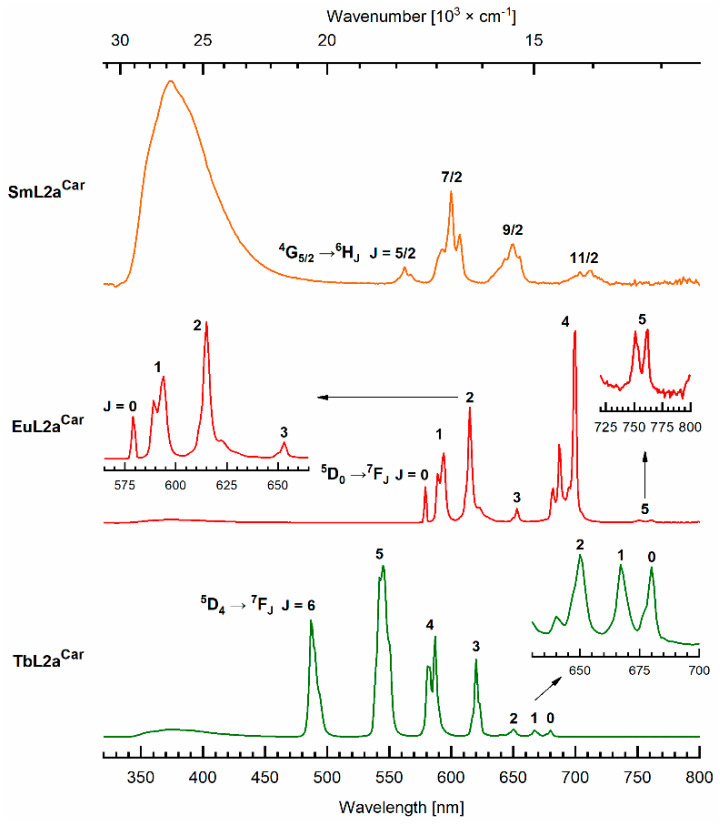
Steady-state emission spectra of **SmL2a^Car^** (top, orange), **EuL2a^Car^** (middle, red) and **TbL2a^Car^** (bottom, green) complexes. [**LnL2a^Car^**] = 10 µM and was measured in D_2_O (Ln = Sm and Eu) or aqueous (Ln = Tb) 10 mM PIPES buffer solutions (10 mM) at pH (pD) 6.5 with *λ*_ex_ = 327 nm.

**Figure 7:**
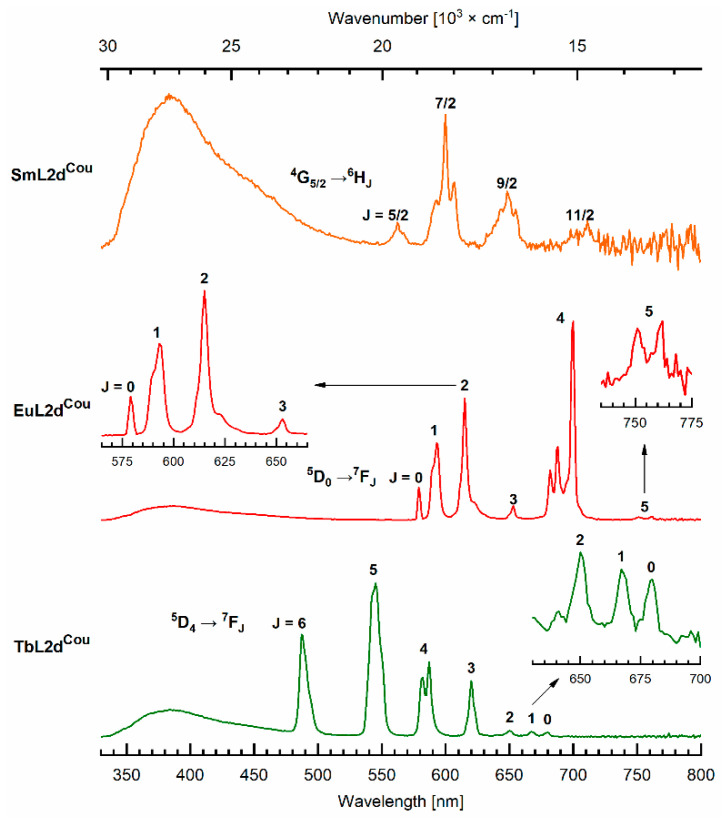
Steady-state emission spectra of **SmL2d^Cou^** (top, orange), **EuL2d^Cou^** (middle, red) and **TbL2d^Cou^** (bottom, green) complexes. [**LnL2d^Cou^**] = 10 µM and was measured in D_2_O (Ln = Sm) or aqueous (Ln = Eu and Tb) 10 mM PIPES buffer solutions (10 mM) at pH (pD) 6.5 with *λ*_ex_ = 315 nm.

Antenna excitation at *λ*_ex_ = 327 or 315 nm yielded Ln(III)-centered emission (Ln = Eu, Tb and Sm) (Figure 6 and Figure 7, Table 2) with all four ligands. The emission spectra along with the assignment of the peaks are shown in Figure 6 and Figure 7. Varying amounts of residual antenna fluorescence were also seen at *λ*_em_ = 375 and 385 nm in the carbostyril and the coumarin-sensitized complexes, respectively. The ligand fluorescence quantum yield (*Φ*_L_) of **EuL2a^Car^** was 3.2 times lower than that of **EuL1a^Car^**. In both Eu(III) triamide and tricarboxylate complexes carrying the coumarin antenna *Φ*_L_ was ~0.60%. The new Eu and Tb complexes had lower Ln(III) emission quantum yields (*Φ*_Ln_) than the previously reported **LnL1a^Car^**. In the case of **LnL2a^Car^** the lower *Φ*_Ln_ is due to the replacement of the tricarboxylate metal-binding fragment with the triamide one. The latter places 6 N-H oscillators close to the Ln(III), which quenches some of the Ln(III) emission. PeT quenching is more efficient for more electron-rich antennae [20] and for more reducible Eu(III) ions [30,36]. As Eu(III) in **EuL2a^Car^** is easier to reduce than in **EuL1a^Car^**, PeT quenching might be more prominent in the former. This suggestion is supported by the significantly smaller *Φ*_L_ value of **EuL2a^Car^** compared to that of **EuL1a^Car^**. *Φ*_Ln_ of **SmL1a^Car^** and **SmL2a^Car^** were similar to those obtained for Sm(III) emitters sensitized by secondary amide-linked carbostyril antennae [20]. 

The coumarin-sensitized complexes were less emissive than their carbostyril analogues (Table 2) irrespective of the Ln(III). For Ln = Tb this may be due to the oxygen sensitivity of the excited complex. However, for Eu(III), the reason is less obvious. The coumarin T_1_ is well-placed for energy transfer to the Eu(III) ^5^D_2_ (21500 cm^−1^) level. Based on the work of Latva, this antenna should provide excellent sensitization [38]. The *Φ*_L_ data do not indicate that there is either PeT quenching of the excited state or direct energy transfer from antenna singlet level (S_1_), as the *Φ*_L_ for the coumarin-sensitized complexes are similar irrespective of the Ln(III) and the type of metal binding site.

Secondary amide-linked carbostyril complexes have previously been shown to sensitize Yb(III) emission [20]. The Yb(III) complexes of the new ligands with tertiary amide linkers were prepared, and their luminescence spectra in the near infrared were recorded. While the sensitivity of the instrument available to us has decreased considerably over the past years (Figures S56 and S57), the relative emission intensities of the previously reported complexes [20] could be compared to those of **YbL1a^Car^** or **YbL2a^Car^** and **YbL1d^Cou^** or **YbL2d^Cou^** (Figure S58). Only very weak emission was observed for **YbL1d^Cou^** and **YbL2d^Cou^**, much weaker than for either the secondary or the tertiary amide-linked carbostyril-sensitized species. As the most likely sensitization pathway in these emitters is via PeT-back electron transfer [30], these data are in line with an inefficient PeT in the coumarin-carrying complexes.

The Ln(III) luminescence lifetimes (*τ*) were measured using time-resolved emission spectroscopy. In PIPES-buffered aqueous solutions the triamide-based Eu(III) complexes had *τ*_H2O_ between 0.51–0.54 ms (Table 3); the lifetimes of the tricarboxylates increased to 0.62–0.66 ms due to the absence of the quenching amide N-H oscillators. The Tb(III) lifetimes in **TbL1a^Car^** and **TbL2a^Car^** were 1.81 ms and 1.50 ms, respectively. In the coumarin-sensitized Tb(III) complexes *τ*_H2O_ was much shorter. This is likely due to the oxygen-sensitivity of these emissions, which also precluded the determination of the hydration states (*q*) of **TbL1d^Cou^** and **TbL2d^Cou^**. For the other Eu(III) and Tb(III) complexes, *q* was found to be ~1 using the Horrocks [17,40] and Beeby [18] method. This value is in accordance with the octadentate ligand structure and the nine-coordinate environment of Eu(III) and Tb(III). The Sm(III) lifetimes were unsurprisingly short, around 10 μs. These lifetimes are similar to what was previously obtained for Sm(III) emitters in DO3A-type ligands [20].

The reason for the low *Φ*_Ln_ of **EuL1d^Cou^** and **EuL2d^Cou^** was next investigated by an analysis of the Eu(III) luminescence spectra. Sensitized Ln(III) emission is the product of multiple processes: photon absorption by the antenna, energy transfer from the antenna to the Ln(III), and Ln(III) luminescence. For Eu(III), it is possible to obtain information on the processes leading to the excited Ln(III) using Equations (1) and (2) [41,42]. The overall luminescence intensity (*Φ*_Ln_) is a product of the luminescent decay of the excited Ln(III), quantified by its intrinsic quantum yield ($\Phi_{Ln}^{Ln}$), and of the sensitization efficiency (*η*_sens_), i.e., the efficiency with which the Ln(III) excited state is populated. $\Phi_{Ln}^{Ln}$ is the ratio of the observed and radiative luminescence lifetimes (*τ*_obs_ and *τ*_rad_, respectively). The latter for Eu(III) can be calculated from the luminescence spectrum using Equation (1), where *A*_MD,0_ is the spontaneous emission probability for the ^5^D_0_→^7^F_1_ transition of Eu(III) in vacuo with a value of 14.65 s^−1^, *n* is the refractive index of the medium (1.333 for H_2_O [43] and 1.328 for D_2_O [44]), and *I*_tot_ and *I*_MD_ are the total corrected Eu(III) emission spectrum (521–800 nm) and the ^5^D_0_→^7^F_1_ band (582–603 nm), respectively [42]. The obtained values are summarized in Table 3.

$\Phi_{Ln}^{Ln}$ quantifies the extent to which the excited Ln(III) can emit in the given coordination environment; similar values were obtained for both types of complexes in H_2_O and D_2_O. Clearly, the difference in *Φ*_Eu_ for the four Eu(III) emitters is primarily down to differences in *η*_sens_. The highest *η*_sens_ is obtained for **EuL1a^Car^**, and the second highest for **EuL2a^Car^**. The lower *η*_sens_ of **EuL2a^Car^** is likely due to a combination of PeT and also possibly the quenching of the antenna by the amide N-H oscillators. The latter, along with solvent quenching of the antenna, can be eliminated by determining *η*_sens_ in D_2_O (Table 3). Solvent deuteration does indeed result in a small increase in *η*_sens_ across the board. The differences in *η*_sens_ between the **EuL1a^Car^** (80.3%) and **EuL2a^Car^** (29.8%), on the one hand, and their coumarin analogues **EuL1d^Cou^** (23.0%) and **EuL2d^Cou^** (16.0%), on the other, remain large.

(1) $$\frac{1}{\tau_{rad}}=A_{MD,0}\times n^{3}\left(\frac{I_{tot}}{I_{MD}}\right)$$

(2) $$\Phi_{Ln}=\eta_{\mathrm{sens}}\cdot \Phi_{Ln}^{Ln}=\eta_{\mathrm{sens}}\cdot \frac{\tau_{\mathrm{obs}}}{\tau_{\mathrm{rad}}}$$

The addition of external fluoride to a solution of **EuL2b^Car^** was shown to increase *Φ*_Eu_ up to 7.6-fold [36]. Most of this increase could be ascribed to Eu(III) stabilization by the negatively charged F^−^ ligand, which reduced PeT quenching. This was apparent from the 3.35-fold larger *η*_sens_ in the presence of KF compared to *η*_sens_ without KF. To evaluate the cause of the low *η*_sens_ in **EuL1d^Cou^** and **EuL2d^Cou^** their photophysical properties in the presence of a large excess of KF (**EuL-F**) in PIPES-buffered H_2_O (Table 4) and D_2_O (Table 5) solutions were determined.

KF addition increased *Φ*_L_ and *Φ*_Eu_ 2.73-fold and 4.56-fold in the case of triamide **EuL2a^Car^-F**. This increase was somewhat smaller than seen for the secondary amide **EuL2b^Car^-F**, suggesting that PeT quenching was smaller in former. The increase in *Φ*_L_ and *Φ*_Eu_ in the case of **EuL1a^Car^-F** was negligible, either because PeT quenching in the absence of fluoride was already small, or because fluoride binding was ineffective due to the overall −1 charge of the complex. The absence of fluoride binding was supported by the fact that the **EuL1a^Car^-F** and **EuL1d^Cou^-F** both retained their water ligand and had *q* = 1 (Figures S63 and S65). **EuL2a^Car^-F** and **EuL2d^Cou^-F** on the other hand had *q* = 0, which is in accordance with the presence of a fluoride ligand instead of a water molecule (Table S28, Figures S64 and S66).

Fluoride binding increased *Φ*_Eu_ in **EuL2d^Cou^-F** 2.2-fold. This increase was ascribable to an improved intrinsic quantum yield (1.96-fold increase), which in turn was caused by the removal of the inner-sphere water molecule (*q* = 0). Changes in *Φ*_L_ and *η*_sens_ were negligible, which suggests that the low *Φ*_Eu_ in **EuL2d^Cou^** was not due to PeT quenching. 

When fluoride addition was carried out in D_2_O, ~2.6-fold higher *Φ*_Eu_ was recorded for the triamide complexes compared to what was obtained in H_2_O. For the tricarboxylates, the increase was on average 3.5-fold. These changes are consistent with the removal of outer-sphere X-H oscillators, as shown by a comparable increase in the intrinsic quantum yields. The greater *Φ*_Eu_ increase for **EuL1** further supports the hypothesis that fluoride does not bind to these Eu(III) centers, and thus, X-H oscillators are only removed by deuteration rather than ligand exchange. 

Having excluded PeT quenching as the major cause of the low *η*_sens_, poor energy transfer efficiency and inefficient population of the antenna feeding levels remain as possible alternatives. While these were not investigated in depth, we note that the steady-state and time-resolved emission spectra of Eu(III) complexes at 77 K did not contain the triplet emission bands from the antennae (Figure S71). This indicates that T_1_ was quenched in these complexes, possibly by energy transfer to Eu(III).

## 3. Materials and Methods

### 3.1. General Procedures

^1^H NMR (400 MHz), ^13^C NMR (100 MHz) and ^19^F NMR (376 MHz) spectra were recorded on a JEOL 400 MHz instrument (JEOL RESONANCE Inc., Tokyo, Japan). Chemical shifts were referenced to residual solvent peaks and are given as follows: chemical shift (δ, ppm), multiplicity (s, singlet; br, broad; d, doublet, t, triplet; q, quartet; m, multiplet), coupling constant (Hz), integration. LC-MS analysis was carried out using an analytical Dionex UltiMate 3000 HPLC instrument (Dionex Softron GmbH, Germering, Germany) coupled to a Thermo Finnigan LCQ DECA XP MAX mass spectrometer (Thermo ELECTRON CORPORATION, San Jose, CA, USA). HR-ESI-MS analyses were performed at the Organisch Chemisches Institut WWU Münster, Germany or at the Stenhagen Analyslab AB, Mölndal, Sweden. All compounds displayed the expected isotope distribution pattern. Anhydrous CH_2_Cl_2_ was obtained by distillation from CaH_2_ under an Ar atmosphere.

Compounds **1** [19], **6** [19], **L1a^Car^** [19], **LnL1a^Car^** (Ln = Eu, Gd, Tb) [19] and **L1d^Cou^** [20] were synthesized following literature methods. All other chemicals were from commercial sources (Sigma Aldrich, St. Louis, MO, USA or Fluorochem, Hadfield, UK) and used as received.

### 3.2. Paramagnetic ^1^H NMR

^1^H NMR spectra of Eu complexes were recorded at 400 MHz using the following parameters: cooling for 5 min until the temperature stabilizes at 0 ± 0.1 °C for samples measured in CD_3_OD and at 10 ± 0.1 °C for samples measured in D_2_O; relaxation delay: 1 s; number of scans: 128; number of points: 131,072; range: −60 to 60 ppm. For Yb complexes measured at r.t. the number of points were 524,288 and the range was from −240 to 240 ppm.

### 3.3. Chromatography

Preparative chromatography was carried out on silica gel [Normasil 60 chromatographic silica media (40–63 micron)] and aluminum oxide [activated, neutral, Brockmann Activity I, Sigma-Aldrich (Sigma Aldrich, St. Louis, MO, USA)]. Thin layer chromatography was performed on silica-coated (60G F254) glass plates from Merck and aluminum oxide coated with 254 nm fluorescent indicator aluminum plates from Sigma-Aldrich. Samples were visualized by UV-light (UVP LLC, Upland, CA, USA) (254 and 365 nm).

HPLC-analysis was performed on a Dionex UltiMate 3000 system (Dionex Softron GmbH, Germering, Germany) using a Phenomenex Gemini^®^ C18 TMS end-capped 150 mm × 4.6 mm HPLC column with HPLC water (0.05% formic acid): CH_3_CN (0.05% formic acid) eluent system using the methods: (a) 0–8 min: 10→20% and 8–12 min: 20% iso and 12–16 min 20→90% CH_3_CN, 0.5 mL/min; (b) 0–8 min: 10% iso and 8–12 min: 10%→50% and 12–16 min 50%→90% CH_3_CN, 0.25 mL/min. UV-Vis (UltiMate 3000 Photodiode Array Detector (Dionex Softron GmbH, Germering, Germany)) and ESI-MS detections (Thermo Finnigan LCQ DECA XP MAX (Thermo ELECTRON CORPORATION, San Jose, CA, USA)) were used. Semi-preparative HPLC was performed on Dionex UltiMate 3000 system (Dionex Softron GmbH, Germering, Germany) using a Phenomenex Gemini^®^ C18 TMS end-capped 150 mm × 30 mm HPLC column with water (0.05% formic acid): MeOH (0.05% formic acid) eluent system with the same UV-detection. The method utilized for semi-preparative purification was the following: 0–6 min: 14% iso and 6–9 min: 95% iso & 9–12 min: 14% iso MeOH, 25 mL/min.

### 3.4. Electrochemistry

Cyclic voltammograms (CV) were obtained in an argon atmosphere at room temperature (~20 °C) using an AUTOLAB PGSTAT 100 potentiostat, or an AUTOLAB PGSTAT 204N potentiostat, equipped with a 3 mm glassy carbon (GC) working electrode, a Pt wire auxiliary electrode, and a saturated calomel electrode (SCE) as a reference. The solution was stirred between each measurement. The solution was allowed to equilibrate for 10 s at the start potential before starting the measurements. A step potential of −0.9 mV was used for 50, 100 and 200 mV/s scan rates, and of −2 mV was used for 500 and 1000 mV/s scan rates. For measurements in aqueous media the supporting electrolyte was LiCl (0.1 M), in the case of non-aqueous (DMF) solutions it was TBAPF_6_ (0.1 M).

General procedure for CV measurements in water: a solution of LiCl (0.1 M) was prepared and pH was set to ~6.5 by addition of NaOH (0.1 M) or HCl (0.1 M). This solution was added to the electrochemical cell, allowed to stir and purged with argon for 10 min prior to each measurement. The working electrode was polished with 0.05 µm alumina on a polishing pad, washed with water and ethanol and dried with air. The three electrodes (GC working electrode, Pt wire auxiliary electrode, and SCE reference electrode) were inserted into the cell setup and a background scan was recorded with a scan rate of 100 mV/s, and four sweeps. A lack of oxygen redox signal verified that oxygen had been removed below detectable levels. The Eu complex (1 mM) was added in the solution, and the pH of the resulting solution was adjusted to ~6.5 (Table S1) by addition of NaOH (0.1 M) or HCl (0.1 M). The resulting solution was stirred and purged with argon for 10 min. Scans were recorded at various scan rates (50 to 1000 mV/s) with four sweeps for each measurement. The voltammograms obtained at various scan rates are shown in Figures S28–S34. The anodic and cathodic peak current intensities (*I*_pa_ and *I*_pc_, respectively) were plotted vs. the square root of scan rate and fit to a linear regression to ensure that the electron transfer was heterogenous.

General procedure for CV measurements in DMF: a sample of TBAPF_6_ (194 mg) was dissolved in 5 mL of DMF (0.1 M) and purged with argon for 10 min. After detecting blank signal without oxygen redox events, the CVs were recorded as it is described in the procedure for aqueous media, with 1 mM concentration of Eu complex. At the end of each experiment a sample of Ferrocene (Fc) was added at the tip of the spatula into the electrochemical cell to adjust potentials according to Fc^0^/Fc^+^ redox events vs SCE which was then shifted according to the difference vs NHE [45]. The cyclic voltammograms of increasing scan rates are displayed in Figures S35–S41.

### 3.5. UV-Vis Absorption and Emission Spectroscopy

All measurements were performed in PIPES-buffered HPLC water or D_2_O at pH 6.5 or pD 6.5. [**LnL**] was nominally 10 µM; however, small quantities of Ln salts may diminish this. Glycerol was of 99.9+% purity. Quartz cells with 1 cm optical pathlengths were used for the room temperature measurements. The absorbance spectra were measured by a Varian Cary 100 Bio UV-Visible spectrophotometer (VARIAN AUSTRALIA PTY LTD, Mulgrave, Victoria, Australia). The emission and excitation spectra, lifetimes, time-resolved spectra and quantum yields were recorded on a Horiba FluoroMax-4P (HORIBA Jobin Yvon, Edison, NJ, USA). All emissions were corrected by the wavelength sensitivity (correction function) of the spectrometer. All measurements were performed at room temperature unless stated otherwise.

Quantum yields were measured at room temperature, using quinine sulfate (QS) in H_2_SO_4_ 0.05 M (*Φ*_ref_ = 0.59) as reference [39] in Equation (3). Quantum yields were calculated according to (3), with *Φ*_s_ the quantum yield of the sample, *Φ*_ref_ the quantum yield of the reference, *I* the integrated corrected emission intensity of the sample (s) and of the reference (ref), *f*_A_ the absorption factor of the sample (s) and of the reference (ref) at the excitation wavelength and *n* the refractive indexes of the sample (s) and of the reference (ref). The concentration of the complexes was adjusted to obtain an absorbance around the maxima of the antennae matching that of the QS fluorescence standard. The excitation wavelength where the absorption factors of the samples and of the reference were the same was chosen (i.e., where the absorptions are identical). The corrected emission spectra of the sample and reference standard were then measured under the same conditions over the 330–800 nm (320-800 nm for carbostyril complexes) spectral range as well as blank samples containing only the solvent (i.e., PIPES-buffered aqueous solutions). The appropriate blanks were subtracted from their respective spectra, and the antenna fluorescence and Ln(III) luminescence were separated by fitting the section of the antenna emission overlapping the Ln(III) emission with an exponential decay or with a scaled emission spectrum from the corresponding Gd(III) complexes. The quantum yields were then calculated according to (3). The given relative error on the quantum yields (δ*Φ* = Δ*Φ*/*Φ*, where Δ*Φ* is the absolute error) take into account the accuracy of the spectrometer and of the integration procedure [δ(*I*_s_/*I*_ref_) < 2%], an error of 0.59 ± 0.01 on the quantum yield of the reference QS [δ(*Φ*_ref_) < 2%], an error on the ratio of the absorption factors [δ(*f*_Aref_/*f*_As_) < 5%, relative to the fixed absorption factor of the reference QS] and an error on the ratio of the squared refractive indexes [δ(*n*_s_^2^/*n*_ref_^2^) < 1%, < 0.25% around 1.333 for H_2_O [43] and 1.328 for D_2_O [44] on each individual refractive index], which sums to a total estimated relative error that should be δ*Φ*_s_ < 10%. A limit value of 10% is thus chosen.

(3) $$\Phi =\frac{I_{s}}{I_{\mathrm{ref}}}\;\times \;\frac{f_{\mathrm{Aref}}}{f_{\mathrm{As}}}\;\times \;\frac{{\left(n_{s}\right)}^{2}}{{\left(n_{\mathrm{ref}}\right)}^{2}}\;\times \Phi_{\mathrm{ref}}$$

Low temperature measurements were done in quartz capillaries (0.2 cm optical pathlength) at 77 K by immersion in a liquid N_2_-filled quartz Dewar and with addition of glycerol (1 drop) to the solutions (9 drops) measured at room temperature.

Lifetimes in the millisecond range were recorded 0.05 ms after pulsed excitation at the excitation maxima (*λ*_ex_) of either 315 (coumarin) or 327 nm (carbostyril) by measuring the decay of the lanthanide main emission peak (i.e., Sm 600 nm, Eu 615 nm and Tb 545 nm). The increments after the initial delay were adjusted between 0.2–20 μs depending on the lifetime in order to have a good sampling of the decay. The obtained data were fitted by single and double exponential decay models in OriginPro 9 (OriginLab Corporation, Northampton, MA, USA), and the most reliable value was chosen according to the adjusted R^2^ value and the shape of the residuals. A relative error of 10% is typically found among a series of measurements on the same sample.

Hydration numbers (*q*) were obtained by measuring the lifetimes of the same quantity of complex in a PIPES buffered solution in H_2_O and in D_2_O and fitting the difference according to the model of Horrocks et al. [17], and Beeby et al. [18].

The NIR emission and excitation spectra were recorded on a Horiba Jobin Yvon Fluorolog3-22 instrument (HORIBA Jobin Yvon, Edison, NJ, USA), and were automatically corrected for wavelength dependent instrument sensitivity.

### 3.6. Synthetic Procedures and Characterization Data

**4**. Known compound [22], new procedure. Coumarin 2 (**4**, 2.00 g, 9.21 mmol) and triethylamine (3.85 mL, 27.6 mmol, 3.0 equiv.) were dissolved in 28.2 mL DMF. The solution was cooled to 0 °C and chloroacetyl chloride (3.19 mL, 36.8 mmol, 4.0 equiv.) was added dropwise while stirring. After 1 h at 0 °C the solution was allowed to warm to r.t. The reaction proceeded for additional 2 h, after which TLC analysis showed full conversion of the starting material. The solution was poured into water (100 mL), which resulted in the formation of a light brown suspension. The mixture was filtered, and the filter cake was washed with water, and the solid was dried in vacuo. The product was obtained as a light brown solid (2.54 g, 94%). ^1^H NMR (400 MHz, CDCl_3_) δ ppm 7.55 (s, 1H), 7.15 (s, 1H), 6.35 (s, 1H), 4.19 (dq, *J* = 14.5, 7.0 Hz, 1H), 3.73 (d, *J* = 2.0 Hz, 2H), 3.26 (dq, *J* = 14.0, 7.0 Hz, 1H), 2.46 (s, 3H), 2.33 (s, 3H), 1.17 (t, *J* = 7.0 Hz, 3H); ^13^C NMR (101 MHz, CDCl_3_) δ (ppm) = 165.7, 160.2, 152.2, 151.4, 142.4, 132.2, 127.4, 120.6, 117.8, 116.3, 44.2, 41.7, 18.8, 17.6, 12.7; HR-ESI-MS obsd 316.07095, calcd 316.07109 [(M + Na)^+^, M = C_15_H_16_NO_3_Cl].

**L2c^Car^**. A sample of **1** (188 mg, 0.354 mmol) was dissolved in DMF (5.1 mL) and DIPEA (308 µL, 1.77 mmol, 5.0 equiv.) was added, followed by the addition of 2-bromoacetamide (161 mg, 1.17 mmol, 3.3 equiv.). The reaction mixture was let to stir overnight at r.t. When LCMS analysis showed full conversion to the product, DMF was removed by co-evaporation with toluene. The concentrated reaction mass was suspended in an equal mixture of THF:Et_2_O and an off-white precipitate was formed which was filtered and washed with diethyl ether (209 mg, 83%). ^1^H NMR (400 MHz, DMSO-*d*6) δ ppm 1.34–1.51 (s, 9H), 2.10–2.48 and 2.53–4.55 (m, 32 H (29H + 3H solvent residuals), 4.67 (s, 2H), 6.52 (s, 1H), 6.98–7.93 (m, 9H), 11.84 (s, 1H); ^13^C NMR (101 MHz, DMSO-*d*6) δ ppm 27.7, 48.6, 48.7, 50.1, 50.4, 51.5, 51.8, 52.1, 52.2, 53.6, 54.5, 54.7, 55.6, 56.3, 58.2, 70.2, 81.4, 81.6, 113.8, 117.0, 119.7, 120.9, 125.8, 139.7, 143.1, 146.8, 161.6, 167.9, 170.5, 170.7, 173.2; RP-HPLC t_R_ = 1.72, 3.55 min, method (a) from general procedures; ESI-MS obsd 702.57, calcd 702.39 (M + H)^+^; HR-ESI-MS obsd 370.6766, calcd 370.6782 [(M + K + H)^2+^, M = C_33_H_51_N_9_O_8_].

**L2a^Car^**. **Method A**. A sample of **L2c^Car^** (100 mg, 0.142 mmol) was dissolved in a 1:1 mixture of CH_2_Cl_2_ and TFA (3.2 mL) and this solution was let to stir for overnight at r.t. After full conversion was observed the solvents were removed on the rotary evaporator (co-evaporation with toluene). The crude product was purified on semi-preparative HPLC using the method described in the general procedures to yield a brownish-white solid (78 mg, 85%). For characterization see **Method B**.

**Method B**. An excess of TFA (0.76 mL) was added into the vial containing **L2c^Car^** (48 mg, 0.068 mmol). The sticky solid of **L2c^Car^** was sonicated with TFA until complete dissolving. The resulting solution was transferred into a 5 mL round bottom flask and CH_2_Cl_2_ was added (0.76 mL). The formed beige suspension was stirred overnight at r.t. The next day CH_2_Cl_2_ was removed under low pressure and TFA was removed via evaporation in a mixture with toluene (5 mL). The remaining oily residue was dissolved in 0.5 mL of MeOH and a large excess of Et_2_O (5 mL) was added. The precipitated product was filtered, dissolved in MeOH and the solution was dried under vacuum to afford beige solid (44 mg, 99%). RP-HPLC t_R_ = 2.35 min, method (b) from general procedures; ^1^H NMR (400 MHz, D_2_O) δ ppm 2.70–4.75 (m, 31H), 6.61 (s, 1H), 7.33 (dd, *J*_1_ = 8.5 Hz, *J*_2_ = 2.0 Hz, 1H), 7.44 (d, *J* = 2.0 Hz, 1H), 7.81 (d, *J* = 8.5 Hz, 1H); ^13^C NMR (101 MHz, D_2_O) δ ppm 47.5–52.5, 51.7, 54.0, 54.7, 55.2, 58.4, 70.3, 115.2, 118.5, 118.7, 122.3, 126.3, 138.4, 142.4 (br), 148.8, 164.1, 165.0–176.0 (br), 172.8; ESI-MS obsd 646.51, calcd 646.33 (M + H)^+^; HR-ESI-MS obsd 646.3316, calcd 646.3313 [(M + H)^+^, M = C_29_H_43_N_9_O_8_].

**L2d^Cou^**. Compound **6** (400 mg, 0.932 mmol, 1.0 equiv.), 2-bromoacetamide (514 mg, 3.73 mmol, 4.0 equiv.) and K_2_CO_3_ (773 mg, 5.59 mmol, 6.0 equiv.) were suspended in MeCN (9.3 mL) and stirred at 60 °C for 2.5 days. The solids were filtered off and the filtrate was concentrated under reduced pressure. The isolated solid was the target product together with an excess of potassium carbonate. Therefore, the solid mixture was dissolved in a minimal amount of MeOH and filtrated from K_2_CO_3_. After evaporation of the solvent the product was obtained as a light brown solid (442 mg, 79%). ^1^H NMR (400 MHz, CD_3_OD) δ ppm 7.75 (s, 1H), 7.29 (s, 1H), 6.34 (s, 1H), 4.09 (dq, *J* = 14.0, 7.1 Hz, 1H), 3.95–3.64 (m, 3H), 3.27 (s, 3H), 3.18–2.39 (m, 20H), 2.32 (s, 3H), 2.08–2.00 (m, 2H), 1.12 (t, *J* = 7.0 Hz, 3H); ^13^C NMR (101 MHz, CD_3_OD) δ ppm 176.1, 171.6, 162.5, 161.4, 158.9, 154.6, 153.3, 144.0, 129.0, 121.6, 118.6, 116.2, 64.6, 62.9, 62.4, 58.3, 44.8, 44.4, 17.5, 13.0; HR-ESI-MS obsd: 623.32682. calcd: 623.32760 [(M + Na)^+^, M = C_29_H_44_N_8_O_6_].

### 3.7. General Procedure for Ln (III) Complexation

A sample of the appropriate ligand (1 equiv.) in the mixture with the corresponding anhydrous LnCl_3_ (1.05 equiv. for **LnL1-2a,c^Car^** and 1.50 equiv. for **LnL1-2d^Cou^**) were dissolved in H_2_O:EtOH equal mixture (0.05 M). For the synthesis of **LnL1a^Car^** only water (0.02 M) was used due to the low solubility of **L1a^Car^** in ethanol. The reaction mixtures were stirred at 55 °C for 24 h. The completion of the complexation was observed via TLC analysis. The purification of **LnL1a^Car^** was done with column chromatography on neutral alumina (MeCN:H_2_O:NH_4_OH (30% aqueous solution), 80:20:3 drops→50:50:9 drops) of the reaction mixtures yielding colorless complexes. The complexes of **LnL2a^Car^** were purified via semi-preparative HPLC using the method described in the general procedures. The coumarin complexes **LnL1-2d^Cou^** and **LnL2c^Car^** were used for characterization without further purification. The isolated compounds contain traces of initial lanthanide chlorides.

**LaL1a^Car^**. 8 mg (65%). ^1^H NMR (400 MHz, D_2_O, 2 isomers in 1:0.05 ratio, major isomer reported) δ ppm 1.96–4.61 ppm (m, 30.45H both isomers + 3H MeOH), 3.51 (s, 3H), 4.84 (s, 2H), 6.78 (s, 1H), 7.39 (dd, *J*_1_ = 8.5 Hz, *J*_2_= 2.1 Hz, 1H), 7.54 (s, 1H), 7.93 (d, *J* = 8.5 Hz, 1H); ESI-MS obsd 783.12, calcd 783.15 [(M)^−^, M = C_29_H_36_N_6_O_11_La]; *λ*_em_ = 375 nm (*λ*_ex_ = 327 nm).

**SmL1a^Car^**. 9 mg (72%). ESI-MS obsd 796.18, calcd 796.16 [(M)^−^, M = C_29_H_36_N_6_O_11_Sm]; *λ*_em_ = 376, 563, 568, 593, 601, 608, 644, 651, 656, 704, 713 nm (*λ*_ex_ = 327 nm).

**YbL1a^Car^**. 12 mg (93%). ESI-MS obsd 818.17, calcd 818.18 [(M)^−^, M = C_29_H_36_N_6_O_11_Yb]; *λ*_em_ = 375 nm (*λ*_ex_ = 327 nm).

**LuL1a^Car^**. 10 mg (77%). ^1^H NMR (400 MHz, D_2_O, 2 isomers in 1:0.05 ratio, major isomer reported) δ ppm 2.25–4.15 ppm (m, 30.45H both isomers + 3H MeOH), 3.51 (s, 3H), 4.85 (s, 2H), 6.79 (s, 1H), 7.43 (d, *J* = 8.5 Hz, 1H), 7.57 (s, 1H), 7.93 (d, *J* = 8.5 Hz, 1H); ESI-MS obsd 819.19, calcd 819.19 [(M)^−^, M = C_29_H_36_N_6_O_11_Lu]; *λ*_em_ = 375 nm (*λ*_ex_ = 327 nm).

**LaL2a^Car^**. 31 mg (quant.). ^1^H NMR (400 MHz, D_2_O, 2 isomers in 1:0.2 ratio, major isomer reported) δ ppm 1.97–3.23 and 3.28–4.75 ppm (m, 37.2H both isomers), 3.53 (s, 3H), 6.77 (s, 1H), 7.34 (d, *J* = 9.0 Hz, 1H), 7.48 (s, 1H), 7.91 (d, *J* = 8.0 Hz, 1H); ESI-MS obsd 783.20, calcd 783.22 [(M)^+^, M = C_29_H_42_N_9_O_8_La]; *λ*_em_ = 373 nm (*λ*_ex_ = 327 nm).

**SmL2a^Car^**. 23 mg (quant.). ESI-MS obsd 796.24, calcd 796.24 [(M)^+^, M = C_29_H_42_N_9_O_8_Sm]; *λ*_em_ = 374, 562, 567, 593, 600, 607, 643, 650, 654, 704, 712 nm (*λ*_ex_ = 327 nm).

**EuL2a^Car^**. 13 mg (47%). RP-HPLC t_R_ = 1.17 min, method (a) from general procedures; ESI-MS obsd 398.72, calcd 398.62 (M)^2+^; HR-ESI-MS obsd 398.61797, calcd 398.61801 [(M)^2+^, M = C_29_H_42_N_9_O_8_Eu]; *λ*_em_ = 375, 579, 589, 594, 615, 622, 653, 682, 687, 695, 699, 752, 760 nm (*λ*_ex_ = 327 nm).

**GdL2a^Car^**. 6 mg (44%). RP-HPLC t_R_ = 1.18 min, method (a) from general procedures; ESI-MS obsd 401.13, calcd 401.12 (M)^2+^; HR-ESI-MS obsd 401.11986, calcd 401.11965 [(M)^2+^, M = C_29_H_42_N_9_O_8_Gd]; *λ*_em_ = 375 nm (*λ*_ex_ = 327 nm).

**TbL2a^Car^**. 9 mg (65%). RP-HPLC t_R_ = 1.18 min, method (a) from general procedures; ESI-MS obsd 401.80, calcd 401.62 (M)^2+^; HR-ESI-MS obsd 401.61996, calcd 401.61994 [(M)^2+^, M = C_29_H_42_N_9_O_8_Tb]; *λ*_em_ = 374, 487, 542, 545, 581, 587, 620, 640, 650, 667, 680 nm (*λ*_ex_ = 327 nm).

**YbL2a^Car^**. 14 mg (74%). ESI-MS obsd 817.19, calcd 817.25 [(M − H)^−^, M = C_29_H_42_N_9_O_8_Yb]; *λ*_em_ = 374 nm (*λ*_ex_ = 327 nm).

**LuL2a^Car^**. 20 mg (quant.). ^1^H NMR (400 MHz, D_2_O, 2 isomers in 1:0.2 ratio, major isomer reported) δ ppm 2.44–3.00 and 3.30–4.17 ppm (m, 37.2H both isomers), 3.51 (s, 3H), 6.79 (s, 1H), 7.38 (d, *J* = 8.0 Hz, 1H), 7.55 (s, 1H), 7.93 (d, *J* = 8.5 Hz, 1H); ESI-MS obsd 818.31, calcd 818.25 [(M − H)^−^, M = C_29_H_42_N_9_O_8_Lu]; *λ*_em_ = 373 nm (*λ*_ex_ = 327 nm).

**LaL2c^Car^**. 4.7 mg (92%). Partial characterization due to the complex being unstable. ^1^H NMR (400 MHz, CD_3_OD, mixture of **LaL2a^Car^** and **LaL2c^Car^** in 1:0.45 ratio, **LaL2c^Car^** peaks reported) δ ppm 1.30 (s, 4.05 H), 2.10-3.27 and 3.57–4.35 ppm (m, 37.7H both species), 3.51 (s, 4.35H), 4.76 (s, 2.9H), 6.76 (s, 0.45H), 7.41 (m, 0.45H), 7.58 (s, 0.45H), 7.89 (d, *J* = 8.0 Hz, 0.45H); *λ*_em_ = 374 nm (*λ*_ex_ = 327 nm). 

**SmL2c^Car^**. 5.1 mg (98%). Partial characterization due to the complex being unstable. *λ*_em_ = 374, 562, 567, 593, 600, 607, 643, 650, 654, 704, 711 nm (*λ*_ex_ = 327 nm).

**EuL2c^Car^**. 6.4 mg (91%). Partial characterization due to the complex being unstable. RP-HPLC t_R_ = 1.28 min, method (a) from general procedures; ESI-MS obsd 449.62, calcd 449.14 (M + Na − H)^2+^, M = C_33_H_51_N_9_O_8_Eu; *λ*_em_ = 374, 579, 589, 594, 615, 623, 653, 682, 687, 695, 699, 752, 761 nm (*λ*_ex_ = 327 nm). HRMS shows only the hydrolyzed complex **EuL2a^Car^**. The LCMS obtained after overnight stirring shows 1:2 ratio of hydrolyzed:^t^Bu species (Figure S1).

**GdL2c^Car^**. 4.9 mg (70%). Partial characterization due to the complex being unstable. RP-HPLC t_R_ = 1.27 min, method (a) from general procedures; ESI-MS obsd 451.70, calcd 451.64 (M + Na − H)^2+^, M = C_33_H_51_N_9_O_8_Gd; *λ*_em_ = 374 nm (*λ*_ex_ = 327 nm). HRMS shows only the hydrolyzed complexes **GdL2a^Car^**. The LCMS obtained after overnight stirring shows 1:2 ratio of hydrolyzed:^t^Bu species (Figure S2).

**TbL2c^Car^**. 3.8 mg (54%). Partial characterization due to the complex being unstable. RP-HPLC t_R_ = 1.27 min, method (a) from general procedures; ESI-MS obsd 452.57, calcd 452.14 (M + Na − H)^2+^, M = C_33_H_51_N_9_O_8_Tb; *λ*_em_ = 373, 487, 545, 545, 582, 587, 620, 640, 650, 667, 680 nm (*λ*_ex_ = 327 nm). HRMS shows only the hydrolyzed complexes **TbL2a^Car^**. The LCMS obtained after overnight stirring shows 1:2 ratio of hydrolyzed:^t^Bu species (Figure S3).

**YbL2c^Car^**. 5 mg (98%). Partial characterization due to the complex being unstable. *λ*_em_ = 374 nm (*λ*_ex_ = 327 nm).

**LuL2c^Car^**. 4.8 mg (94%). Partial characterization due to the complex being unstable. ^1^H NMR (400 MHz, CD_3_OD, mixture of **LaL2a^Car^** and **LaL2c^Car^** in 1:0.45 ratio, both species reported) δ ppm 1.29 (s, 2.25 H), 2.47–3.08 and 3.57–4.00 ppm (m, 32.5H both species), 3.51 (s, 3.75H), 4.76 (s, 2.5H), 6.76–6.79 (s, 1.25H), 7.37 (dd, *J*_1_ = 8.5 Hz, *J*_2_ = 2.0 Hz, 1.25H), 7.58 (s, 1.25H), 7.91–7.98 (d, *J* = 8.0 Hz, 1.25H); *λ*_em_ = 375 nm (*λ*_ex_ = 327 nm).

**LaL1d^Cou^**. 27 mg (quant.); ^1^H NMR (400 MHz, D_2_O, 2 isomers in 1:0.33 ratio, major isomer reported) δ ppm 1.16 (t, *J* = 7.0 Hz, 3H), 2.36 (s, 3H), 2.50 (s, 3H) 2.82–3.90 ppm (m, 33.25H both isomers + 3H MeOH), 4.19 (dq, *J*_1_ = 15.0 Hz, *J*_2_ = 7.5 Hz, 1H), 6.46 (s, 1H), 7.48 (s, 1H), 7.86 (s, 1H); HR-ESI-MS *m*/*z*: obsd: 762.16330, calcd: 762.16252 [(M + Na)^+^, M = C_29_H_38_N_5_O_9_La]; *λ*_em_ = 387 nm (*λ*_ex_ = 315 nm).

**SmL1d^Cou^**. Known compound [20], new procedure. 16 mg (quant.); HR-ESI-MS *m*/*z*: obsd: 775.17683, calcd: 775.17601 [(M + Na)^+^, M = C_29_H_38_N_5_O_9_Sm]; *λ*_em_ = 383, 563, 568, 592, 601, 608, 650 nm (*λ*_ex_ = 315 nm).

**EuL1d^Cou^**. Known compound [20], new procedure. 33 mg (quant.); HR-ESI-MS *m*/*z*: obsd: 754.19657, calcd: 754.19573 [(M + H)^+^, M = C_29_H_38_N_5_O_9_Eu]; *λ*_em_ = 387, 579, 588, 594, 614, 623, 653, 682, 688, 694, 700 nm (*λ*_ex_ = 315 nm).

**GdL1d^Cou^**. Known compound [20], new procedure. 18 mg (quant.); HR-ESI-MS *m*/*z*: obsd: 781.18182, calcd: 781.18098 [(M + Na)^+^, M = C_29_H_38_N_5_O_9_Gd]; *λ*_em_ = 387 nm (*λ*_ex_ = 315 nm).

**TbL1d^Cou^**. Known compound [20], new procedure. 18 mg (quant.); HR-ESI-MS *m*/*z*: obsd: 782.18214, calcd: 782.18152 [(M + Na)^+^, M = C_29_H_38_N_5_O_9_Tb]; *λ*_em_ = 389, 487, 542, 545, 583, 587, 620, 651, 668, 682 nm (*λ*_ex_ = 315 nm).

**YbL1d^Cou^**. 33 mg (quant.); HR-ESI-MS *m*/*z*: obsd: 797.19648, calcd: 797.19553 [(M + Na)^+^, M = C_29_H_38_N_5_O_9_Yb]; *λ*_em_ = 388 nm (*λ*_ex_ = 315 nm).

**LuL1d^Cou^**. Known compound [20], new procedure. 26 mg (quant.); ^1^H NMR (400 MHz, D_2_O, 2 isomers in 1:0.1 ratio, major isomer reported) δ ppm 1.16 (t, *J* = 7.0 Hz, 3H), 2.36 (s, 3H), 2.50 (s, 3H) 2.57–3.72 ppm (m, 27.5H both isomers + 3H MeOH), 4.17 (m, 1H), 6.46 (s, 1H), 7.48 (s, 1H), 7.87 (s, 1H); HR-ESI-MS *m*/*z*: obsd: 798.19742, calcd: 798.19694 [(M + Na)^+^, M = C_29_H_38_N_5_O_9_Lu]; *λ*_em_ = 388 nm (*λ*_ex_ = 315 nm).

**LaL2d^Cou^**. 24 mg (quant.); ^1^H NMR (400 MHz, D_2_O, 2 isomers in 1:0.05 ratio, major isomer reported) δ ppm 1.16 (m, 3H), 2.33 (s, 3H), 2.50 (s, 3H) 2.7–4.27 ppm (m, 27.3H both isomers + 3H MeOH), 6.48 (s, 1H), 7.41 (s, 1H), 7.87 (s, 1H); HR-ESI-MS *m*/*z*: obsd: 369.11778, calcd: 369.11790 [(M − H)^2−^, M = C_29_H_44_N_8_O_6_La]; *λ*_em_ = 387 nm (*λ*_ex_ = 315 nm).

**SmL2d^Cou^**. 16 mg (quant.); HR-ESI-MS *m*/*z*: obsd: 375.62442, calcd: 375.62464 [(M − H)^2−^, M = C_29_H_44_N_8_O_6_Sm]; *λ*_em_ = 385, 563, 567, 593, 600, 606, 648 nm (*λ*_ex_ = 315 nm).

**EuL2d^Cou^**. 32 mg (quant.); HR-ESI-MS *m*/*z*: obsd: 375.12529, calcd: 376.12547 [(M − H)^2−^, M = C_29_H_44_N_8_O_6_Eu]; *λ*_em_ = 387, 579, 593, 615, 623, 653, 682, 688, 700 nm (*λ*_ex_ = 315 nm).

**GdL2d^Cou^**. 17 mg (quant.); HR-ESI-MS *m*/*z*: obsd: 378.62684, calcd: 378.62711 [(M − H)^2−^, M = C_29_H_44_N_8_O_6_Gd]; *λ*_em_ = 386 nm (*λ*_ex_ = 315 nm).

**TbL2d^Cou^**. 17 mg (quant.); HR-ESI-MS *m*/*z*: obsd: 379.12710, calcd: 379.12740 [(M − H)^2−^, M = C_29_H_44_N_8_O_6_Tb]; *λ*_em_ = 387, 487, 545, 582, 587, 620, 650, 667, 680 nm (*λ*_ex_ = 315 nm).

**YbL2d^Cou^**. 32 mg (quant.); HR-ESI-MS *m*/*z*: obsd: 386.63411, calcd: 386.63439 [(M − H)^2−^, M = C_29_H_44_N_8_O_6_Yb]; *λ*_em_ = 387 nm (*λ*_ex_ = 315 nm).

**LuL2d^Cou^**. 25 mg (quant.); ^1^H NMR (400 MHz, D_2_O, 2 isomers in 1:0.15 ratio, major isomer reported) δ ppm 1.17 (t, *J* = 7.0 Hz, 3H), 2.36 (s, 3H), 2.51 (s, 3H) 2.57–3.06 and 3.20–4.25 ppm (m, 29.9H both isomers + 9H 3MeOH), 6.48 (s, 1H), 7.43 (d, *J* = 23.0 Hz, 1H), 7.88 (d, *J* = 10.0 Hz, 1H); HR-ESI-MS *m*/*z*: obsd: 387.13483, calcd: 387.13511 [(M − H)^2−^, M = C_29_H_44_N_8_O_6_Lu]; *λ*_em_ = 388 nm (*λ*_ex_ = 315 nm).

## 4. Conclusions

Octadentate ligands carrying 4-methoxymethylcarbostyril or coumarin 2 sensitizing antennae mounted on a DO3A ligand binding site were prepared and characterized. Paramagnetic ^1^H NMR spectroscopy indicated that the coumarin-carrying ligands existed as a mixture of SAP rotamers, possibly due to the steric clash between the antenna 6-Me group and the linker carbonyl. Only one species was seen for the carbostyrils with H in the 6-position. The Eu(III)/Eu(II) reduction potentials were found to be quite unaffected by the nature of the amide linker (secondary vs tertiary) between the antenna and the metal binding site. The Eu(III) oxidation state was nevertheless stabilized by a negatively charged methylcarboxylate substituent on the tertiary amide linker.

Ln(III) (Ln = Eu, Tb, Sm) emission was detected from both coumarin and carbostyril-sensitized complexes. The coumarin antenna was unexpectedly ineffective in sensitizing Ln(III) emission. In the case of the Eu(III) complexes, the low overall quantum yield appears to be caused by low sensitization efficiency. While a large part of the excitation energy was eliminated due to PeT in the triamide carbostyril complex, this quenching pathway did not seem prominent in the coumarin 2-sensitized +3-charged species. This was indicated by the lack of improvement in Eu(III) sensitization efficiency upon the stabilization of the +3 oxidation state via fluoride binding. These results underscore the importance of understanding the structures of emitters in solution and show that superficially similar complexes can be subject to different quenching processes.

## Figures and Tables

**Table 1:** Cyclic voltammetry of Eu(III) complexes (positively charged complexes are with Cl-counterions) ^[a]^.

Compound	*E*_1/2_^[b]^	*E*_pa_^[b]^	*E*_pc_^[b]^	Δ*E* ^[b]^
EuCl_3_	−384	−337	−431	94
**EuL1a^Car^**	−948 (–839 ^[c]^)	−766	−1131	365
**EuL2a^Car^**	−612 (–554 ^[d]^)	−437	−787	350
**EuL2c^Car^**	−564	−437	−691	254
**EuL1d^Cou^**	−854	−744	−965	221
**EuL2d^Cou^**	−565	−512	−617	105

^[a]^*E*_1/2_ is a half-wave potential, *E*_pa_ (E_pc_) is anodic (cathodic) peak potential, Δ*E* is peak separation. ^[b]^ Values are in mV vs. NHE. Measured in H_2_O (LiCl 0.1 M, pH ~6.5) with a sample concentration of 1 mM at a glassy C electrode using a SCE as reference electrode and a Pt wire counter electrode with a scan rate of 100 mV/s. ^[c]^ *E*_1/2_ of the corresponding secondary amide complex **EuL1b^Car^**, from [36]. ^[d]^ *E*_1/2_ of the corresponding secondary amide complex **EuL2b^Car^**, from [36].

**Table 2:** Antenna- and Ln(III)-based luminescence quantum yields, Ln(III) lifetimes and hydration states of **LnL**.

Complex	*Φ*_L_ [%] ^[a]^	*Φ*_Ln_ [%] ^[a]^	*τ*_H2O_ [ms]	*τ*_D2O_ [ms]	*q* ^[b]^
**LaL1a^Car^**	4.9	-	-	-	-
**SmL1a^Car^**	3.6 ^[c]^	0.19 ^[c]^	0.009	0.032	-
**EuL1a^Car^** ^[d]^	2.5	8.9	0.66	2.17	1.0
**GdL1a^Car^** ^[d]^	5.1	-	-	-	-
**TbL1a^Car^** ^[d]^	4.5	45.1	1.81	2.92	0.8
**YbL1a^Car^**	4.5	-	-	-	-
**LuL1a^Car^**	4.7	-	-	-	-
**LaL2a^Car^**	4.1	-	-	-	-
**SmL2a^Car^**	3.4 ^[c]^	0.21 ^[c]^	0.010	0.032	-
**EuL2a^Car^**	0.77 ^[e]^	2.7 ^[e]^	0.51	2.05	0.9
**GdL2a^Car^**	4.3 ^[c]^	-	-	-	-
**TbL2a^Car^**	4.1	36.6	1.50	2.70	1.2
**YbL2a^Car^**	4.3	-	-	-	-
**LuL2a^Car^**	4.5	-	-	-	-
**LaL1d^Cou^**	0.66	-	-	-	-
**SmL1d^Cou^**	0.64 ^[c]^	0.059 ^[c]^	0.010	0.031	-
**EuL1d^Cou^**	0.62 ^[e]^	2.2 ^[e]^	0.62	1.96	1.0
**GdL1d^Cou^**	0.66 ^[c]^	-	-	-	-
**TbL1d^Cou^**	0.62	1.7	0.47	0.61	-
**YbL1d^Cou^**	0.66	-	-	-	-
**LuL1d^Cou^**	0.64	-	-	-	-
**LaL2d^Cou^**	0.66	-	-	-	-
**SmL2d^Cou^**	0.64 ^[c]^	0.067 ^[c]^	0.012	0.032	-
**EuL2d^Cou^**	0.58 ^[e]^	1.5 ^[e]^	0.54	2.01	0.8
**GdL2d^Cou^**	0.63 ^[c]^	-	-	-	-
**TbL2d^Cou^**	0.67	1.0	0.29	0.45	-
**YbL2d^Cou^**	0.57	-	-	-	-
**LuL2d^Cou^**	0.61	-	-	-	-

Measurements were performed with [**LnL**] = 10 µM in 10 mM aqueous PIPES buffer solutions at pH (pD) 6.5. ^[a]^ Relative to quinine sulfate (*Φ* = 0.59) in H_2_SO_4_ (0.05 M) [39]. ^[b]^ Calculated using the Equation *q* = 5(1/*τ*_H2O_ – 1/*τ*_D2O_ – 0.06) for Tb, and *q* = 1.2(1/*τ*_H2O_ – 1/*τ*_D2O_ – 0.25 – *n* × 0.075), where n is the number of nearby N-H oscillators, for Eu [18]. ^[c]^ Mean for two independent measurements. ^[d]^ Data from [19]. ^[e]^ Mean for three independent measurements.

**Table 3:** Photophysical properties of **EuL** in PIPES-buffered H_2_O and D_2_O solutions ^[a]^.

Complex	*τ*_rad,Ln_^[b]^	${\Phi_{Ln}^{Ln}\;}^{\left[c\right]}$	*η*_sens_^[c]^	*Φ*_L,D2O_^[d]^	*Φ*_Ln,D2O_^[d]^	*τ*_rad,Ln_ (D_2_O) ^[e]^	${\Phi_{Ln}^{Ln}(D_{2}O)\;}^{\left[f\right]}$	*η*_sens_ (D_2_O) ^[f]^
**EuL1a^Car^**	5.36	12.2	72.8	2.6 (1.04)	32.3 (3.6)	5.39	40.3 (3.30)	80.3 (1.10)
**EuL2a^Car^**	5.11	10.0	27.6	0.83 (1.08)	11.8 (4.2)	5.16	39.7 (3.97)	29.8 (1.08)
**EuL1d^Cou^**	5.26	11.8	17.6	0.66 (1.10)	8.5 (4.0)	5.31	36.9 (3.15)	23.0 (1.30)
**EuL2d^Cou^**	5.03	10.7	14.1	0.60 (1.03)	6.3 (4.2)	5.11	39.3 (3.67)	16.0 (1.14)

^[a]^ [**LnL**] = 10 µM in 10 mM aqueous PIPES buffer H_2_O or D_2_O solutions at pH (pD) 6.5. ^[b]^ In ms, determined using Equation (1). ^[c]^ In %, determined using Equation (2). ^[d]^ In %, relative to quinine sulfate (*Φ* = 0.59) in H_2_SO_4_ (0.05 M) [39]. ^[e]^ In ms, determined using Equation (1). [f] In %, determined using Equation (2), in parentheses change relative to the solution in H_2_O.

**Table 4:** Photophysical properties of **EuL-F** in PIPES-buffered H_2_O ^[a]^.

Complex	*Φ*_L_ [%] ^[b]^	*Φ*_Ln_ [%] ^[b]^	*τ*_rad,Ln_^[c]^	*τ*_obs_ [ms]	${\Phi_{Ln}^{Ln}\;}^{\left[d\right]}$	*η*_sens_^[e]^
**EuL1a^Car^-F**	2.5	9.5 (×1.07)	5.33	0.68	12.8 (×1.05)	73.4 (1.01)
**EuL2a^Car^-F**	2.1 (×2.73)	12.3 (×4.56)	4.86	1.01	20.7 (×2.07)	58.9 (2.13)
**EuL1d^Cou^-F**	0.64 (×1.03)	2.3 (×1.10)	5.19	0.69	13.3 (×1.14)	17.3 (0.98)
**EuL2d^Cou^-F**	0.65 (×1.12)	3.3 (×2.20)	4.81	1.01	21.0 (×1.96)	15.7 (1.12)

^[a]^ Formed by the addition of excess KF (0.1 M, 10^4^-fold excess) to a solution of **EuL**. [**EuL**] = 10 µM in 10 mM aqueous PIPES buffer solutions at pH 6.5. In parentheses fold increase compared to **EuL** without added KF. ^[b]^ Relative to quinine sulfate (*Φ* = 0.59) in H_2_SO_4_ (0.05 M) [39]. ^[c]^ In ms, determined using Equation (1). ^[d]^ In %, determined using Equation (2). ^[e]^ In %, determined using Equation (2).

**Table 5:** Antenna- and Eu(III)-based luminescence properties of **EuL-F** in PIPES-buffered D_2_O solution ^[a]^.

Complex	*Φ*_L_ [%] ^[b]^	*Φ*_Ln_ [%] ^[b]^	*τ*_rad,Ln_^[c]^	*τ*_obs_ [ms]	${\Phi_{Ln}^{Ln}\;}^{\left[d\right]}$	*η*_sens_^[e]^
**EuL1a^Car^-F**	2.6 (1.04)	32.3 (3.4)	5.38	2.29	42.6 (3.33)	75.7 (1.03)
**EuL2a^Car^-F**	2.2 (1.05)	31.3 (2.6)	4.89	2.40	49.1 (2.37)	63.7 (1.08)
**EuL1d^Cou^-F**	0.67 (1.06)	8.2 (3.6)	5.29	2.25	42.6 (3.20)	19.2 (1.11)
**EuL2d^Cou^-F**	0.69 (1.08)	8.7 (2.6)	4.88	2.39	48.9 (2.33)	17.8 (1.13)

^[a]^ Formed by the addition of KF (0.1 M, 10^4^-fold excess) to a solution of **EuL**. [**EuL**] = 10 µM in 10 mM PIPES buffer solutions in D_2_O at pD 6.5. In parentheses: fold increase relative to the solution in H_2_O. ^[b]^ Relative to quinine sulfate (*Φ* = 0.59) in H_2_SO_4_ (0.05 M) [39]. ^[c]^ In ms, determined using Equation (1). ^[d]^ In %, determined using Equation (2). ^[e]^ In %, determined using Equation (2).

## Supplementary Materials

The following are available online, characterization data for new compounds (^1^H and ^13^C NMR spectra) and for new complexes (LC-MS analysis for **LnL2c^Car^**, ^1^H NMR spectra), additional photophysical and electrochemical characterization of **LnL**, ^1^H and ^19^F NMR spectra and photophysical characterization of **LnL-F**.
