# Supplementary material for: Lanthanide(III) Complexes of Cyclen Triacetates and Triamides Bearing Tertiary Amide-Linked Antennae
Source: Molecules. 2020 Nov 12;25(22):5282. doi: 10.3390/molecules25225282 (PMC7698001; doi:10.3390/molecules25225282)
Supplement: Supplementary file 1 [file molecules-25-05282-s001.pdf]

Lanthanide(III) complexes of cyclen triacetates and triamides bearing tertiary amide-linked antennae

Salauat R. Kiraev, Emilie Mathieu, Fiona Siemens, Daniel Kovacs, Ellen Demeyere and K. Eszter Borbas*

Department of Chemistry, Ångström Laboratory, Uppsala University, Box 523, 75120 Uppsala, Sweden

Email: eszter.borbas@kemi.uu.se

Table of contents

General experimental procedures	S2
Additional chemical characterization	S8
LC-MS analysis	S8
^{1}H NMR spectra of Ln(III) complexes	S10
Cyclic voltammetry	S21
Photophysical characterization	S38
^{1}H and ^{19}F spectra of Ln(III)-F complexes	S60
^{1}H and ^{13}C NMR spectra	S72
References	S78

General experimental procedures

^{1}H NMR (400 MHz), ^{13}C NMR (100 MHz) and ^{19}F NMR (376 MHz) spectra were recorded on a JEOL 400 MHz instrument (JEOL RESONANCE Inc., Tokyo, Japan). Chemical shifts were referenced to residual solvent peaks and are given as follows: chemical shift (δ, ppm), multiplicity (s, singlet; br, broad; d, doublet, t, triplet; q, quartet; m, multiplet), coupling constant (Hz), integration. LC-MS analysis was carried out using an analytical Dionex UltiMate 3000 HPLC instrument (Dionex Softron GmbH, Germering, Germany) coupled to a Thermo Finnigan LCQ DECA XP MAX mass spectrometer (Thermo ELECTRON CORPORATION, San Jose, California, USA). HR-ESI-MS analyses were performed at the Organisch Chemisches Institut WWU Münster, Germany or at the Stenhagen Analyslab AB, Mölndal. All compounds displayed the expected isotope distribution pattern. Anhydrous CH_2Cl_2 was obtained by distillation from CaH_2 under an Ar atmosphere.

Compounds **1** [1], **6** [1], **L1a^{Car}** [1], **LnL1a^{Car}** (Ln = Eu, Gd, Tb) [1] and **L1d^{Cou}** [2] were synthesized following literature methods. All other chemicals were from commercial sources (Sigma Aldrich, St. Louis, Missouri, USA or Fluorochem, Hadfield, UK) and used as received.

Paramagnetic ^{1}H NMR. ^{1}H NMR spectra of Eu-complexes were recorded at 400 MHz using the following parameters: cooling for 5 min until the temperature stabilizes at 0±0.1 °C for samples measured in CD_3OD and at 10±0.1 °C for samples measured in D_2O; relaxation delay: 1 s; number of scans: 128; number of points: 131,072; range: −60 to 60 ppm. For Yb complexes measured at r.t. the number of points were 524,288 and the range was from −240 to 240 ppm.

Chromatography. Preparative chromatography was carried out on silica gel [Normasil 60 chromatographic silica media (40–63 micron)] and aluminum oxide [activated, neutral,

Brockmann Activity I, Sigma-Aldrich (Sigma Aldrich, St. Louis, Missouri, USA)]. Thin layer chromatography was performed on silica-coated (60G F254) aluminum plates from Merck and aluminum oxide coated with 254 nm fluorescent indicator aluminum plates from Sigma-Aldrich. Samples were visualized by UV-light (UVP LLC, Upland, California, USA) (254 and 365 nm).

HPLC-analysis was performed on a Dionex UltiMate 3000 system (Dionex Softron GmbH, Germering, Germany) using a Phenomenex Gemini® C18 TMS end-capped 150 mm×4.6 mm HPLC column with HPLC water (0.05% formic acid): CH_3CN (0.05% formic acid) eluent system using the methods: (a) 0–8 min: 10→20% & 8–12 min: 20% iso & 12–16 min 20→90% CH_3CN, 0.5 mL/min; (b) 0–8 min: 10% iso & 8–12 min: 10%→50% & 12–16 min 50%→90% CH_3CN, 0.25 mL/min. UV- (UltiMate 3000 Photodiode Array Detector (Dionex Softron GmbH, Germering, Germany)) and ESI-MS detections (Thermo Finnigan LCQ DECA XP MAX (Thermo ELECTRON CORPORATION, San Jose, California, USA)) were used. Semi-preparative HPLC was performed on Dionex UltiMate 3000 system (Dionex Softron GmbH, Germering, Germany) using a Phenomenex Gemini® C18 TMS end-capped 150 mm×30 mm HPLC column with water (0.05% formic acid): MeOH (0.05% formic acid) eluent system with the same UV-detection. The method utilised for semi-preparative purification was the following: 0–6 min: 14% iso & 6–9 min: 95% iso & 9–12 min: 14% iso MeOH, 25 mL/min.

Electrochemistry. Cyclic voltammograms (CV) were obtained in an argon atmosphere at room temperature (~20 °C) using an AUTOLAB PGSTAT 100 potentiostat, or an AUTOLAB PGSTAT 204N potentiostat, equipped with a 3 mm glassy carbon (GC) working electrode, a Pt wire auxiliary electrode, and a saturated calomel electrode (SCE) as a reference. The solution was stirred in between each measurement. The solution was let to equilibrate for 10 s at the start potential before starting the measurements. A step potential of −0.9 mV was used

for 50, 100 and 200 mV/s scan rates, and of −2 mV was used for 500 and 1000 mV/s scan rates. For measurements in aqueous media the supporting electrolyte was LiCl (0.1 M), in case of non-aqueous (DMF) solutions it was $TBAPF_6$ (0.1 M).

General procedure for CV measurements in water: a solution of LiCl (0.1 M) was prepared and pH was set to ~6.5 by addition of NaOH (0.1 M) or HCl (0.1 M). This solution was added to the electrochemical cell, allowed to stir, and purged with argon for 10 min prior to each measurement. The working electrode was polished with 0.05 μm alumina on a polishing pad, washed with water and ethanol, and dried with air. The three electrodes (GC working electrode, Pt wire auxiliary electrode, and SCE reference electrode) were inserted into the cell setup and a background scan was recorded with a scan rate of 100 mV/s, and four sweeps. A lack of oxygen redox signal verified that oxygen had been removed below detectable levels. The Eu complex (1 mM) was added in the solution, and the pH of the resulting solution was adjusted to ~6.5 (Table S1) by addition of NaOH (0.1 M) or HCl (0.1 M). The resulting solution was stirred and purged with argon for 10 min. Scans were recorded at various scan rates (50 to 1000 mV/s) with four sweeps for each measurement. The voltammograms obtained at various scan rates are shown in Figures S28–34. The anodic and cathodic peak current intensities (I_{pa} and I_{pc} respectively) were plotted vs. the square root of scan rate and fit to a linear regression to ensure that the electron transfer was heterogenous.

General procedure for CV measurements in DMF: a sample of $TBAPF_6$ (194 mg) was dissolved in 5 mL of DMF (0.1 M) and purged with argon for 10 minutes. After detecting blank signal without oxygen redox events, the CVs were recorded as it is described in the procedure for aqueous media, with 1 mM concentration of Eu complex. At the end of each experiment a sample of Ferrocene (Fc) was added at the tip of the spatula into the electrochemical cell to adjust potentials according to Fc^0/Fc^+ redox events vs SCE which was

then shifted according to the difference vs NHE [3]. The cyclic voltammograms of increasing scan rates are displayed in Figures S35–41.

UV-Vis absorption and emission spectroscopy. All measurements were performed in PIPES-buffered HPLC water or D_2O at pH 6.5 or pD 6.5. [**LnL**] was nominally 10 µM, however, small quantities of Ln salts may diminish this. Glycerol was of 99.9+% purity. Quartz cells with 1 cm optical pathlengths were used for the room temperature measurements. The absorbance spectra were measured by a Varian Cary 100 Bio UV-Visible spectrophotometer (VARIAN AUSTRALIA PTY LTD, Mulgrave, Victoria, Australia). The emission and excitation spectra, lifetimes, time-resolved spectra and quantum yields were recorded on a Horiba FluoroMax-4P (HORIBA Jobin Yvon, Edison, New Jersey, USA). All emissions were corrected by the wavelength sensitivity (correction function) of the spectrometer. All measurements were performed at room temperature unless stated otherwise.

Quantum yields were measured at room temperature, using quinine sulfate (QS) in H_2SO_4 0.05 M ($\Phi_{ref} = 0.59$) as reference [4] in Equation S1. Quantum yields were calculated according to (3), with Φ_s the quantum yield of the sample, Φ_{ref} the quantum yield of the reference, I the integrated corrected emission intensity of the sample (s) and of the reference (ref), f_A the absorption factor of the sample (s) and of the reference (ref) at the excitation wavelength and n the refractive indexes of the sample (s) and of the reference (ref). The concentration of the complexes was adjusted to obtain an absorbance around the maxima of the antennae matching that of the QS fluorescence standard. The excitation wavelength where the absorption factors of the samples and of the reference were the same was chosen (i.e. where the absorptions are identical). The corrected emission spectra of the sample and reference standard were then measured under the same conditions over the 330–800 nm (320-800 nm for carbostyril complexes) spectral range as well as blank samples containing only the solvent (i.e. PIPES-buffered aqueous solutions). The appropriate blanks were subtracted from

their respective spectra, and the antenna fluorescence and Ln(III) luminescence were separated by fitting the section of the antenna emission overlapping the Ln(III) emission with an exponential decay or with a scaled emission spectrum from the corresponding Gd(III) complexes. The quantum yields were then calculated according to (3). The given relative error on the quantum yields (δΦ = ΔΦ/Φ, where ΔΦ is the absolute error) take into account the accuracy of the spectrometer and of the integration procedure [$\delta(I_s/I_{ref}) < 2\%$], an error of 0.59 ±0.01 on the quantum yield of the reference QS [$\delta(\Phi_{ref}) < 2\%$], an error on the ratio of the absorption factors [$\delta(f_{Aref}/f_{As}) < 5\%$, relative to the fixed absorption factor of the reference QS] and an error on the ratio of the squared refractive indexes [$\delta(n_s^2/n_{ref}^2) < 1\%$, < 0.25% around 1.333 for H_2O [5] and 1.328 for D_2O [6] on each individual refractive index], which sums to a total estimated relative error that should be $\delta\Phi_s < 10\%$. A limit value of 10% is thus chosen.

$$\Phi = \frac{I_s}{I_{ref}} \times \frac{f_{Aref}}{f_{As}} \times \frac{(n_s)^2}{(n_{ref})^2} \times \Phi_{ref} \qquad \text{S1}$$

Low temperature measurements were done in quartz capillaries (0.2 cm optical pathlength) at 77 K by immersion in a liquid N_2-filled quartz Dewar and with addition of glycerol (1 drop) to the solutions (9 drops) measured at room temperature.

Lifetimes were recorded 0.05 ms after pulsed excitation at the excitation maxima (λ_{ex}) of either 315 (coumarin) or 327 nm (carbostyril) by measuring the decay of the lanthanide main emission peak (i.e. Sm 600 nm, Eu 615 nm and Tb 545 nm). The increments after the initial delay were adjusted between 0.2–20 μs depending on the lifetime in order to have a good sampling of the decay. The obtained data were fitted by single and double exponential decay models in OriginPro 9 (OriginLab Corporation, Northampton, Massachusetts, USA), and the most reliable value was chosen according to the adjusted R^2 value and the shape of the

residuals. A relative error of 10% is typically found among a series of measurements on the same sample.

Hydration numbers (*q*) were obtained by measuring the lifetimes of the same quantity of complex in a PIPES buffered solution in H_2O and in D_2O and fitting the difference according to the model of Horrocks et al. [7], and Beeby et al [8].

The NIR emission and excitation spectra were recorded on a Horiba Jobin Yvon Fluorolog3-22 instrument (HORIBA Jobin Yvon, Edison, New Jersey, USA) and automatically corrected for wavelength dependent instrument sensitivity.

Additional chemical characterization

LC-MS analysis

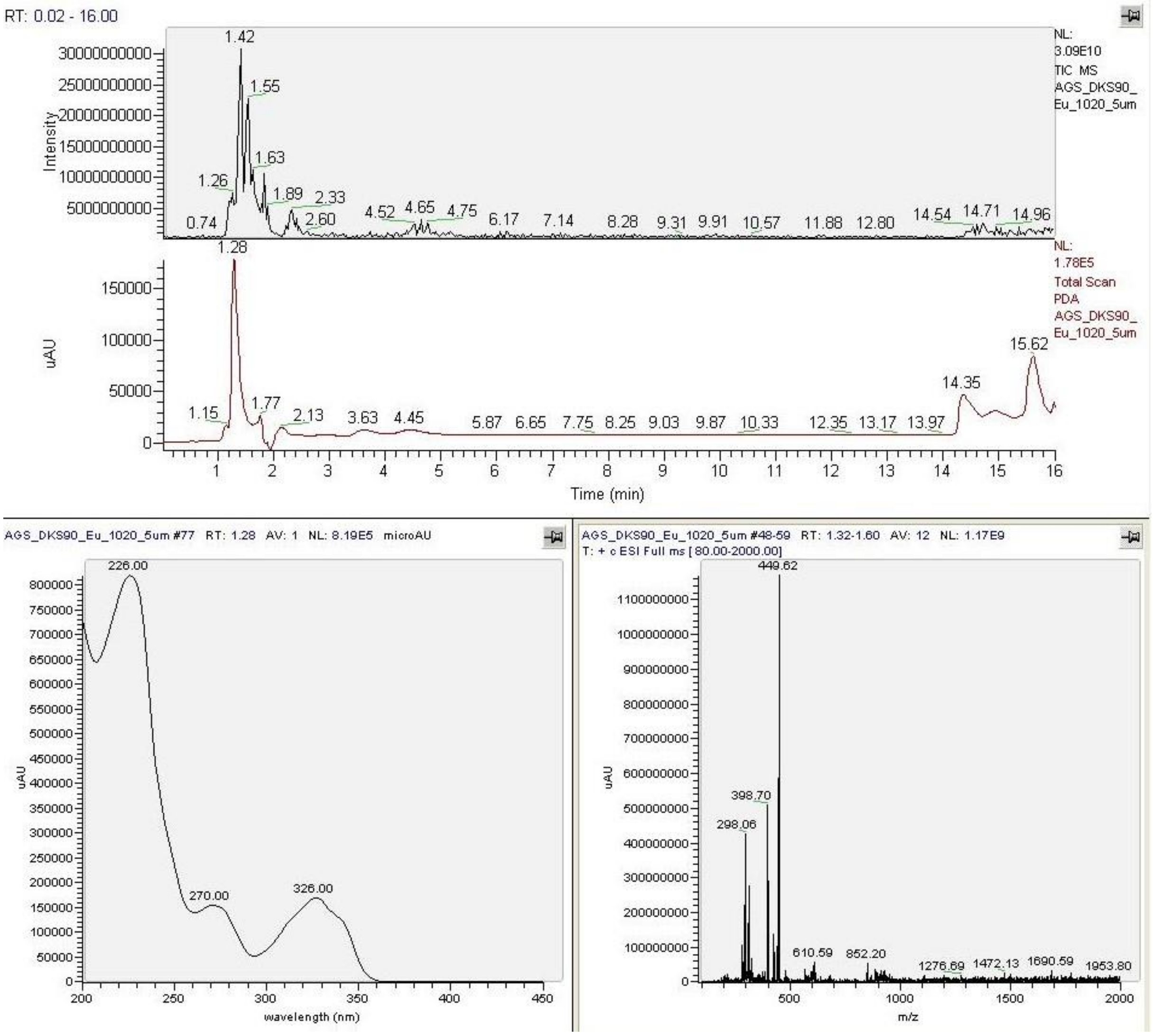

Figure S1. LC-MS analysis of **EuL2c^{Car}** measured via method (a) from general procedures.

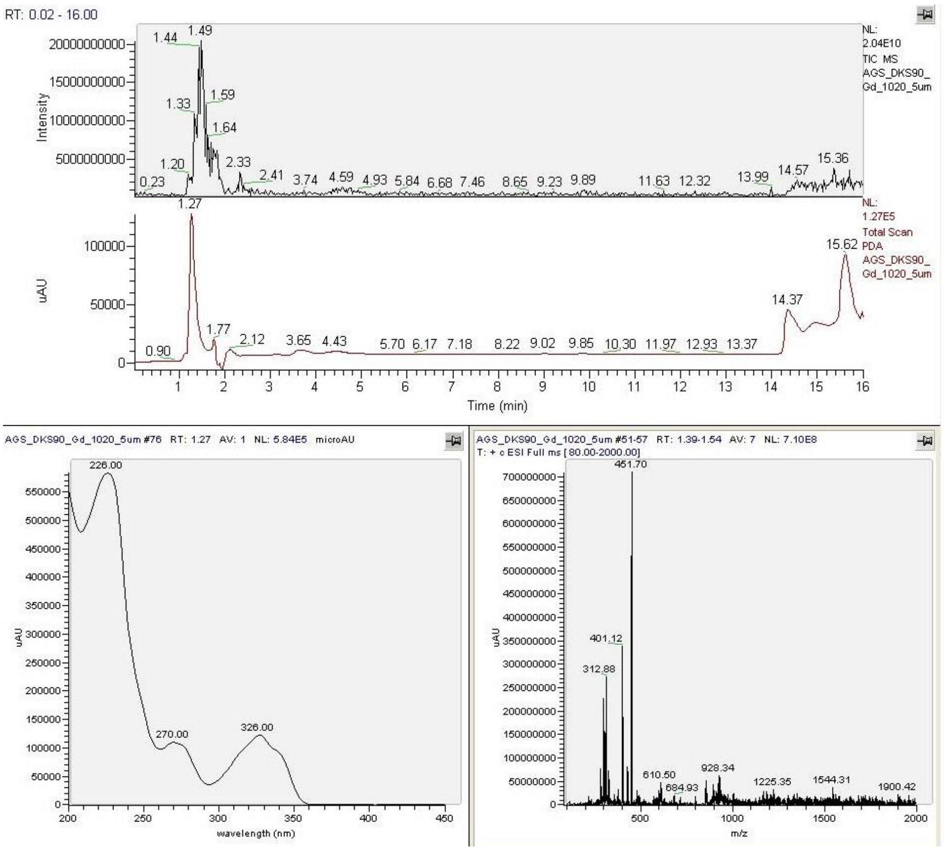

Figure S2. LC-MS analysis of **GdL2c$^{\mathbf{Car}}$** measured via method (a) from general procedures.

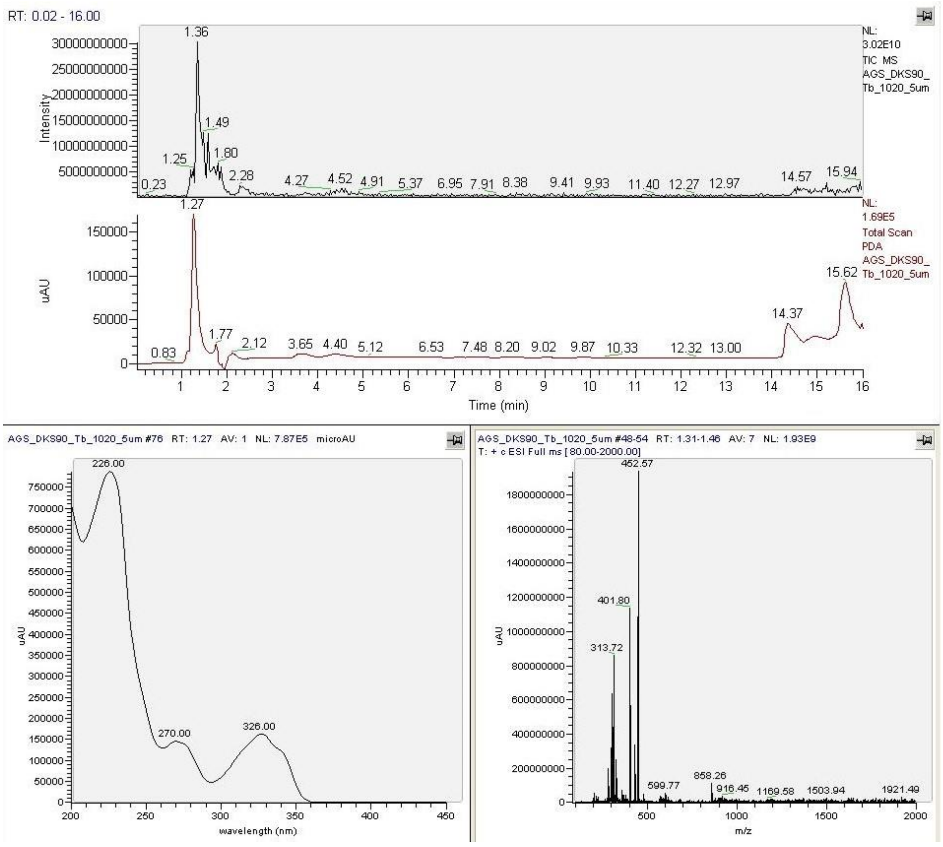

Figure S3. LC-MS analysis of **TbL2c$^{\mathbf{Car}}$** measured via method (a) from general procedures.

^{1}H NMR spectra of Ln(III) complexes

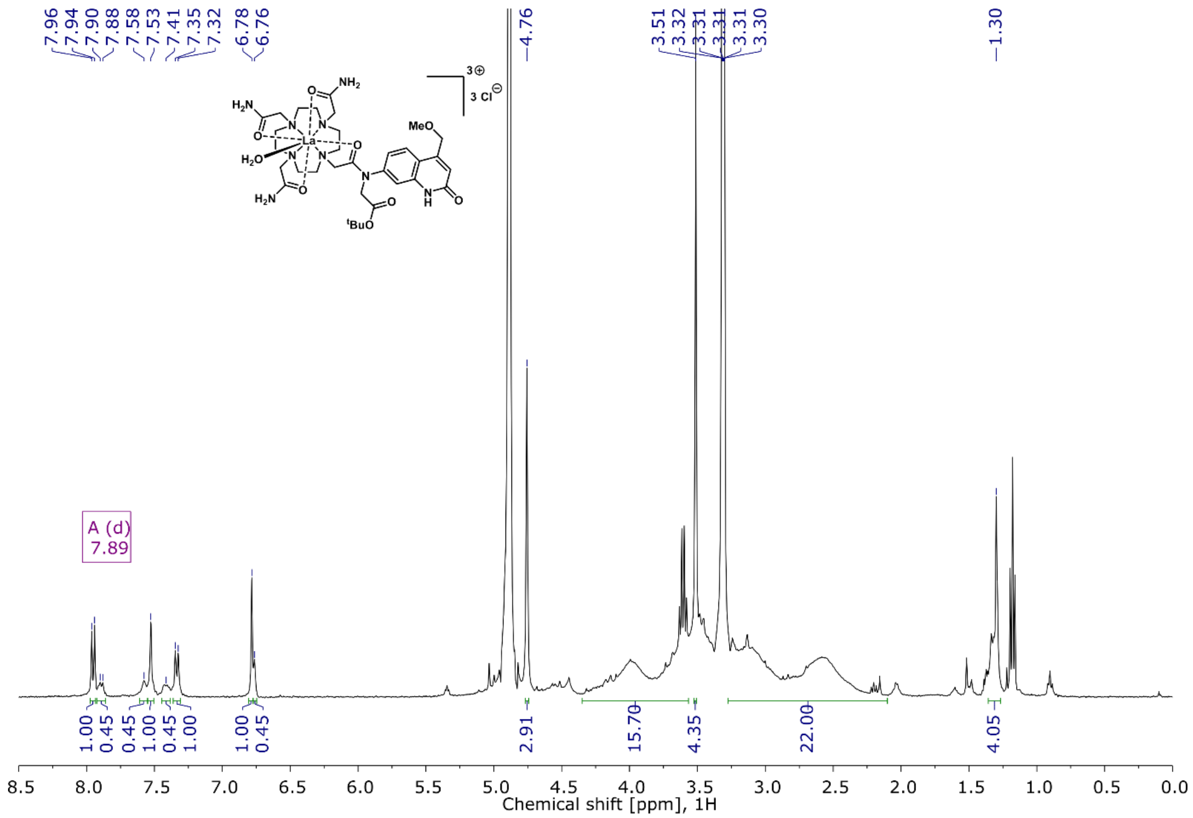

Figure S4. ^{1}H NMR spectrum (400 MHz) of **LaL2c$^{\mathbf{Car}}$** measured in CD_3OD at r.t.

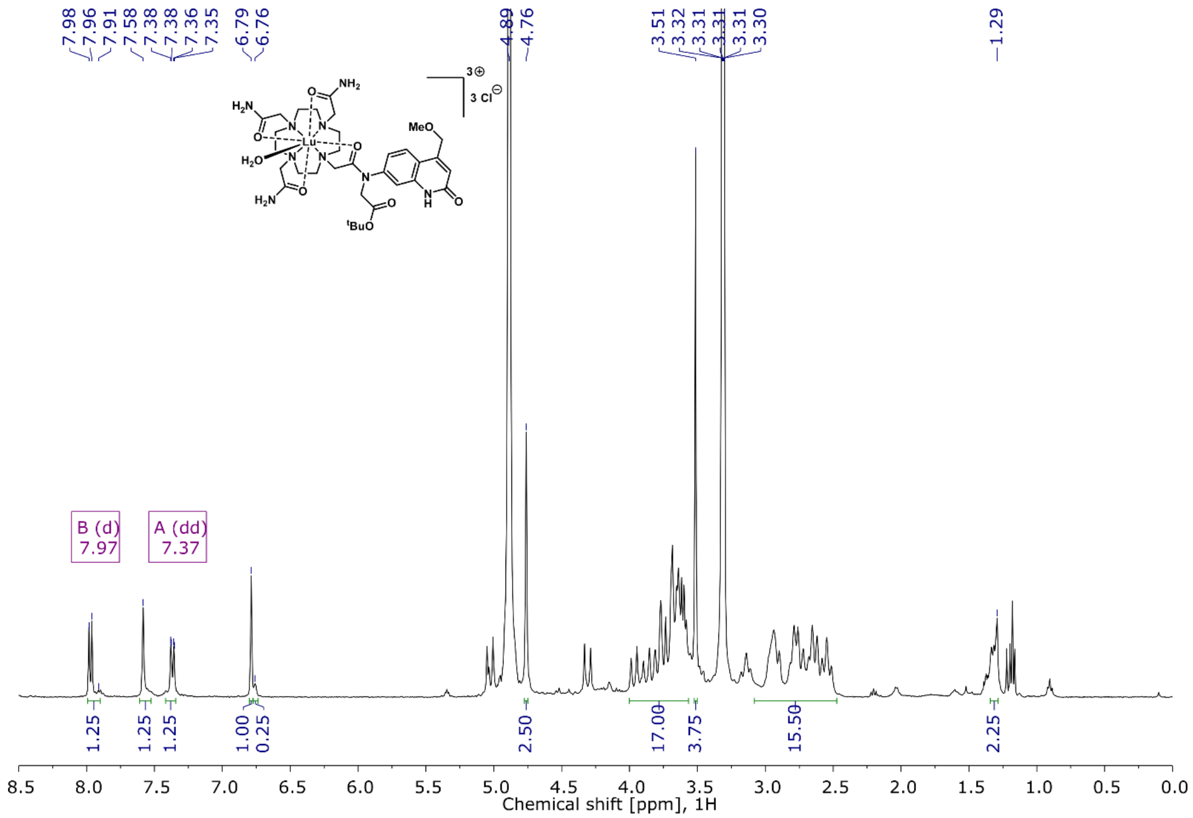

Figure S5. ^{1}H NMR spectrum (400 MHz) of **LuL2c$^{\mathbf{Car}}$** measured in CD_3OD at r.t.

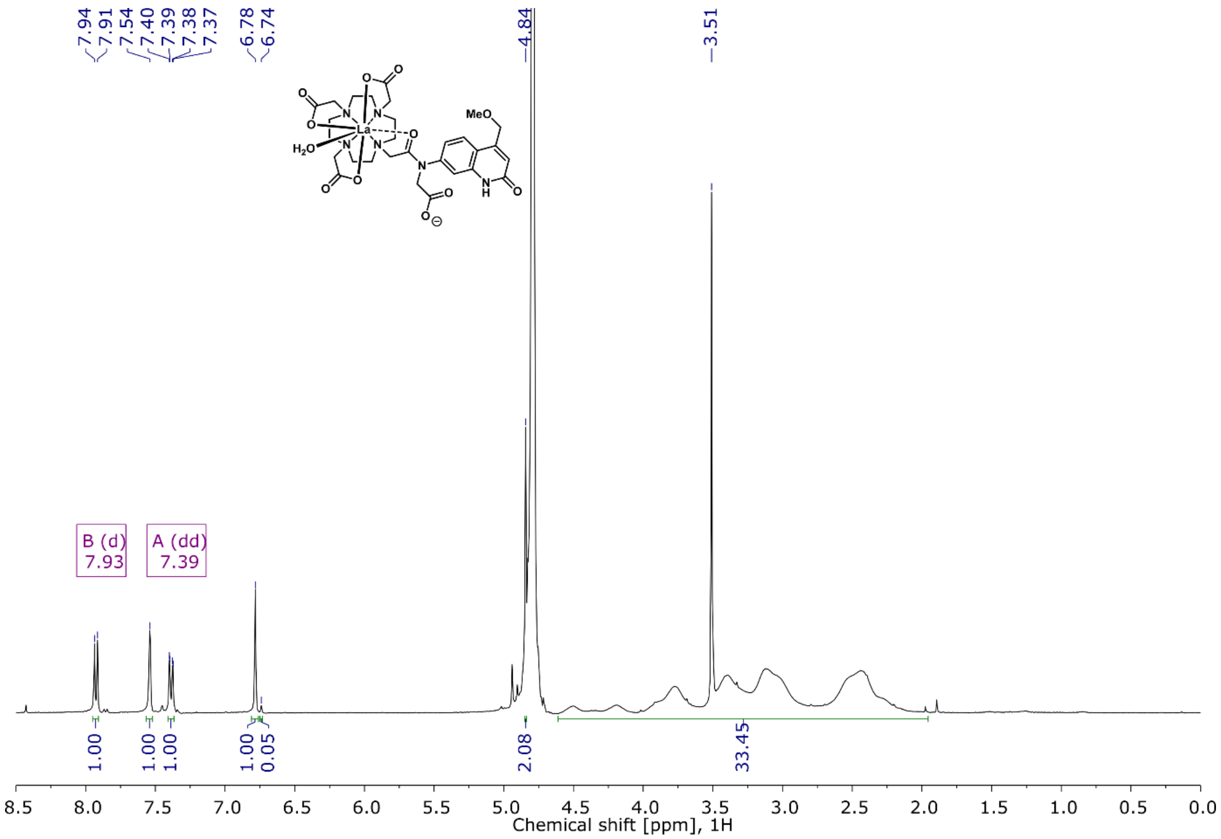

Figure S6. ^{1}H NMR spectrum (400 MHz) of **LaL1a^{Car}** measured in D_2O at r.t.

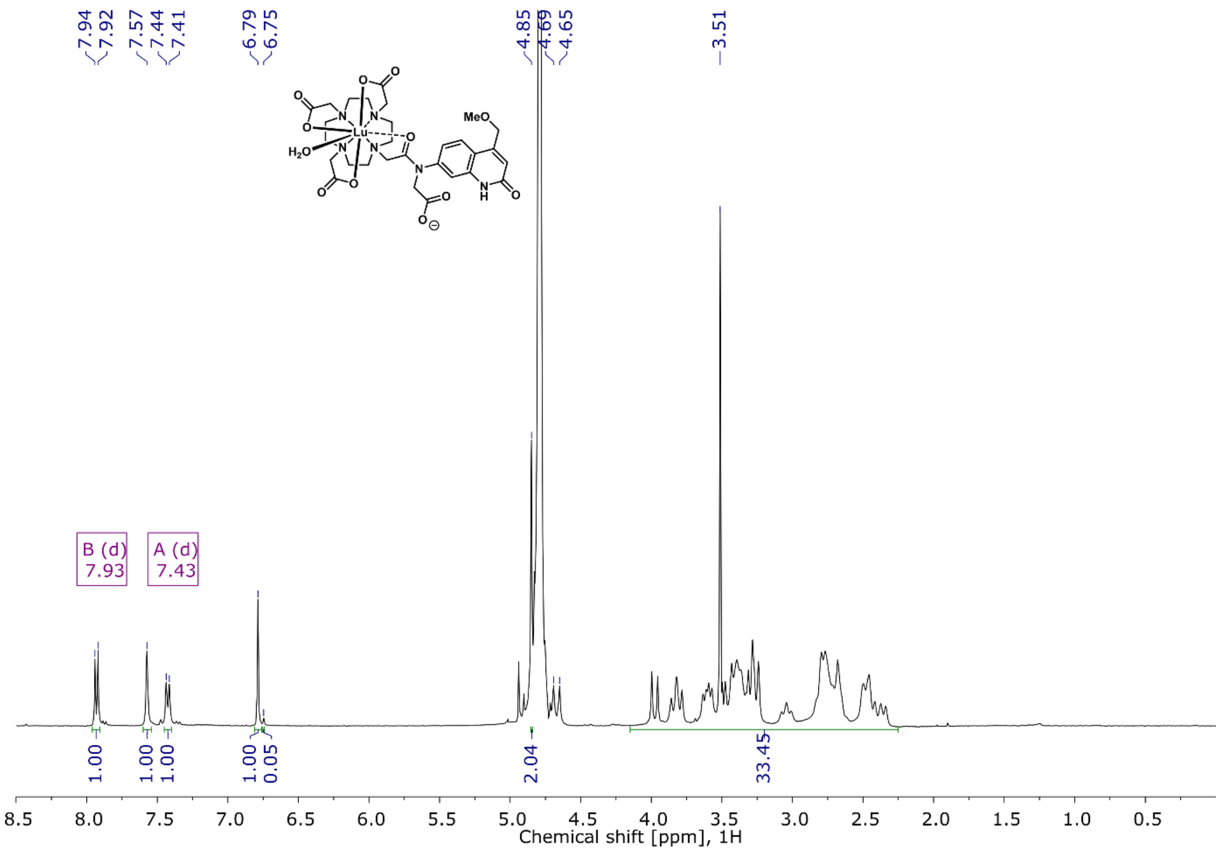

Figure S7. ^{1}H NMR spectrum (400 MHz) of **LuL1a^{Car}** measured in D_2O at r.t.

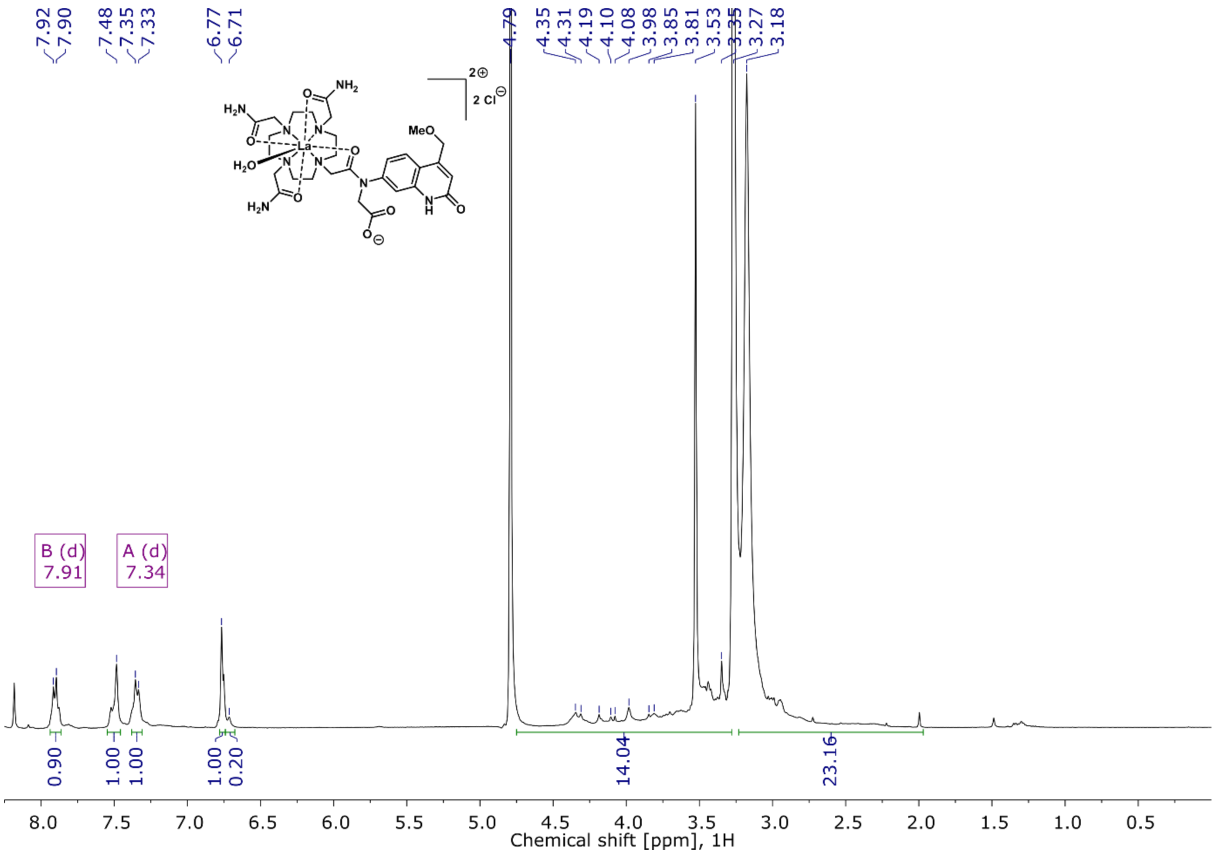

Figure S8. ^{1}H NMR spectrum (400 MHz) of **LaL2a$^{\mathbf{Car}}$** measured in D_2O at r.t.

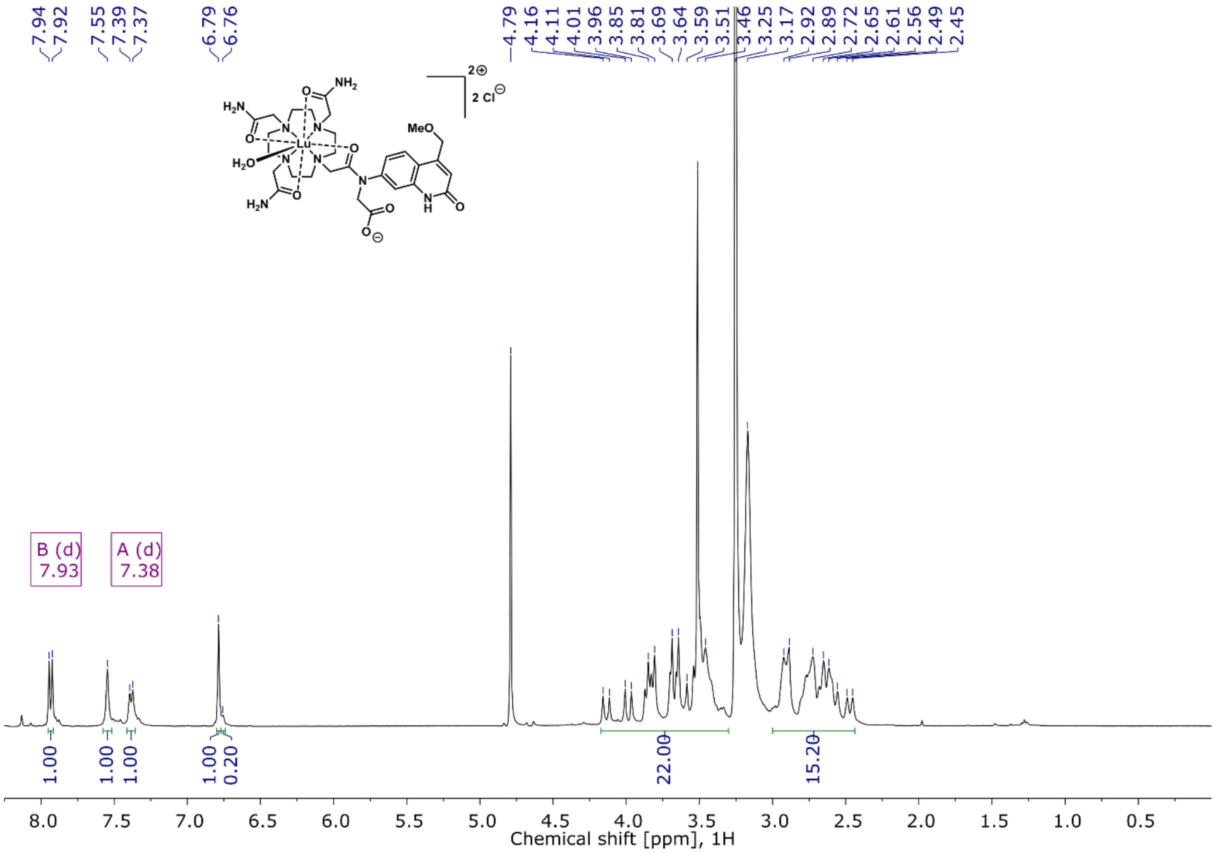

Figure S9. ^{1}H NMR spectrum (400 MHz) of **LuL2a$^{\mathbf{Car}}$** measured in D_2O at r.t.

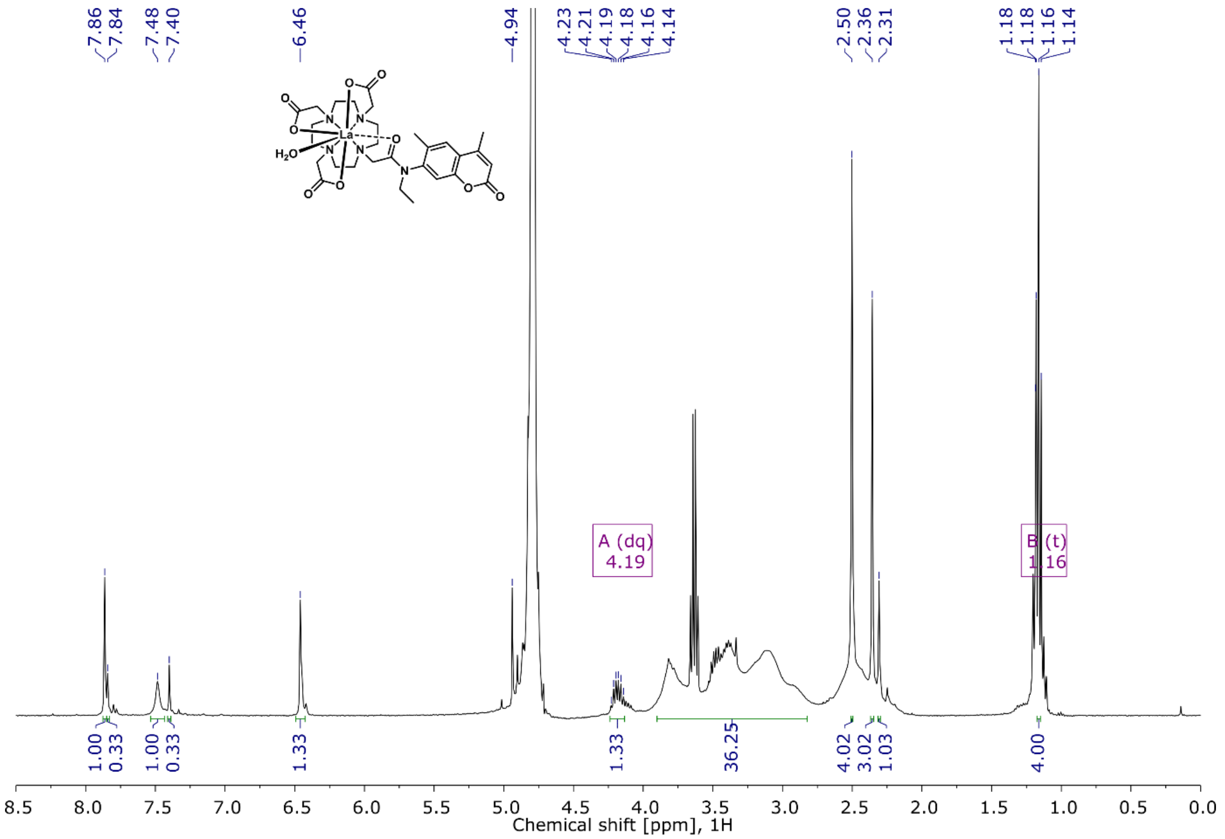

Figure S10. ^{1}H NMR spectrum (400 MHz) of **LaL1d^{Cou}** measured in D_2O at r.t.

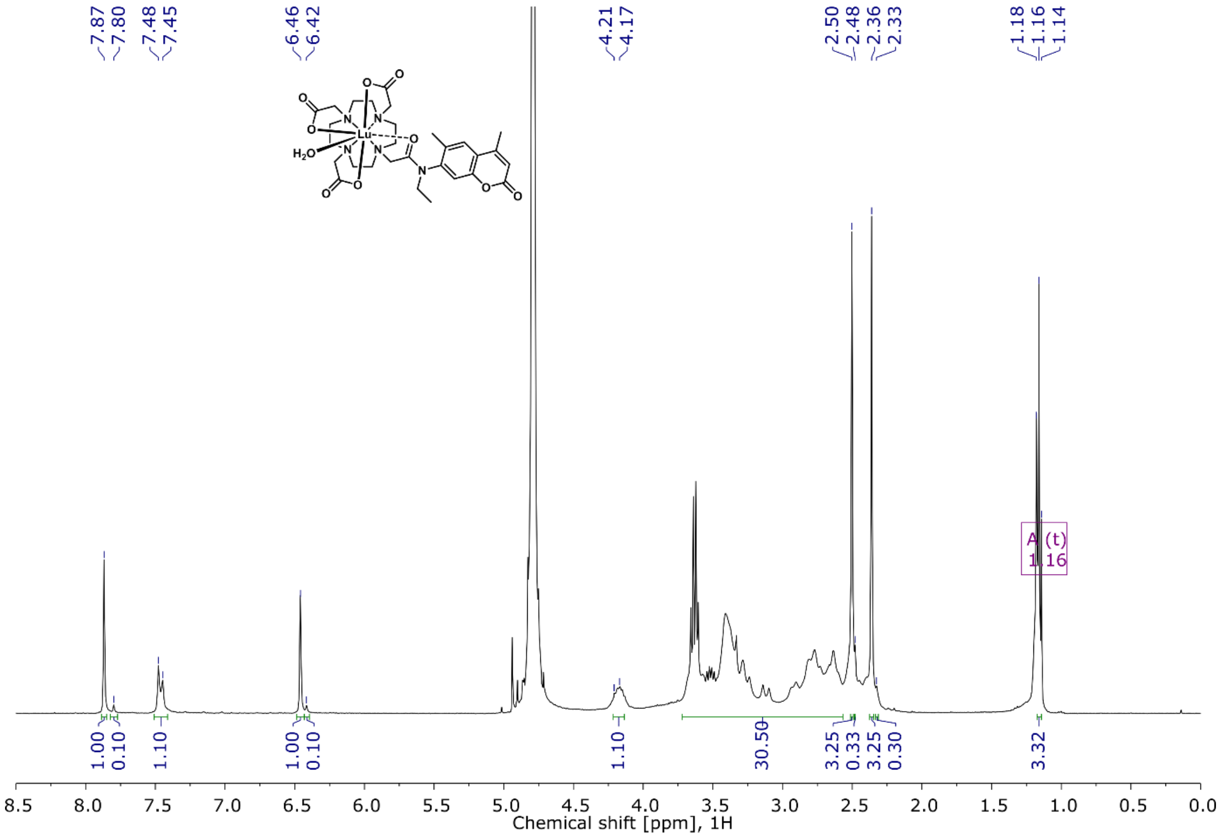

Figure S11. ^{1}H NMR spectrum (400 MHz) of **LuL1d^{Cou}** measured in D_2O at r.t.

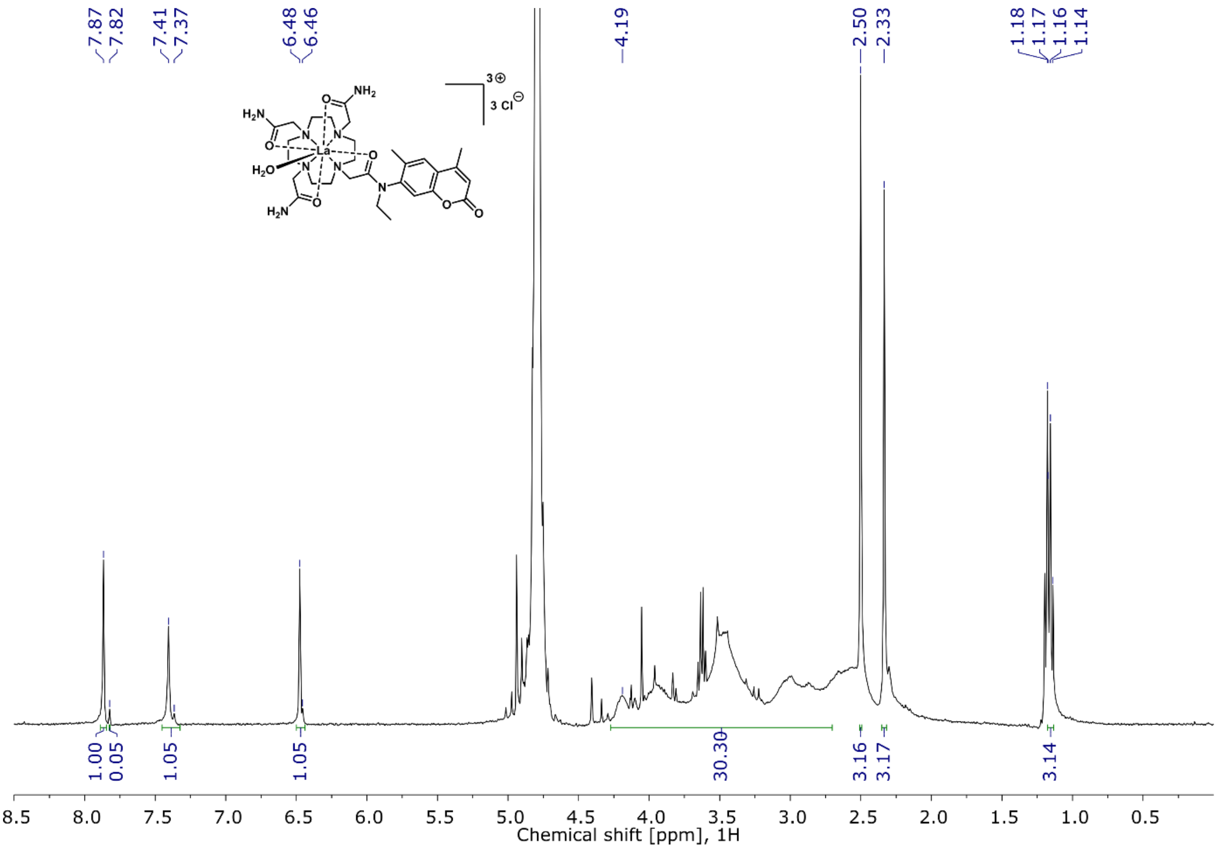

Figure S12. ^{1}H NMR spectrum (400 MHz) of **LaL2d**$^{\mathbf{Cou}}$ measured in D_2O at r.t.

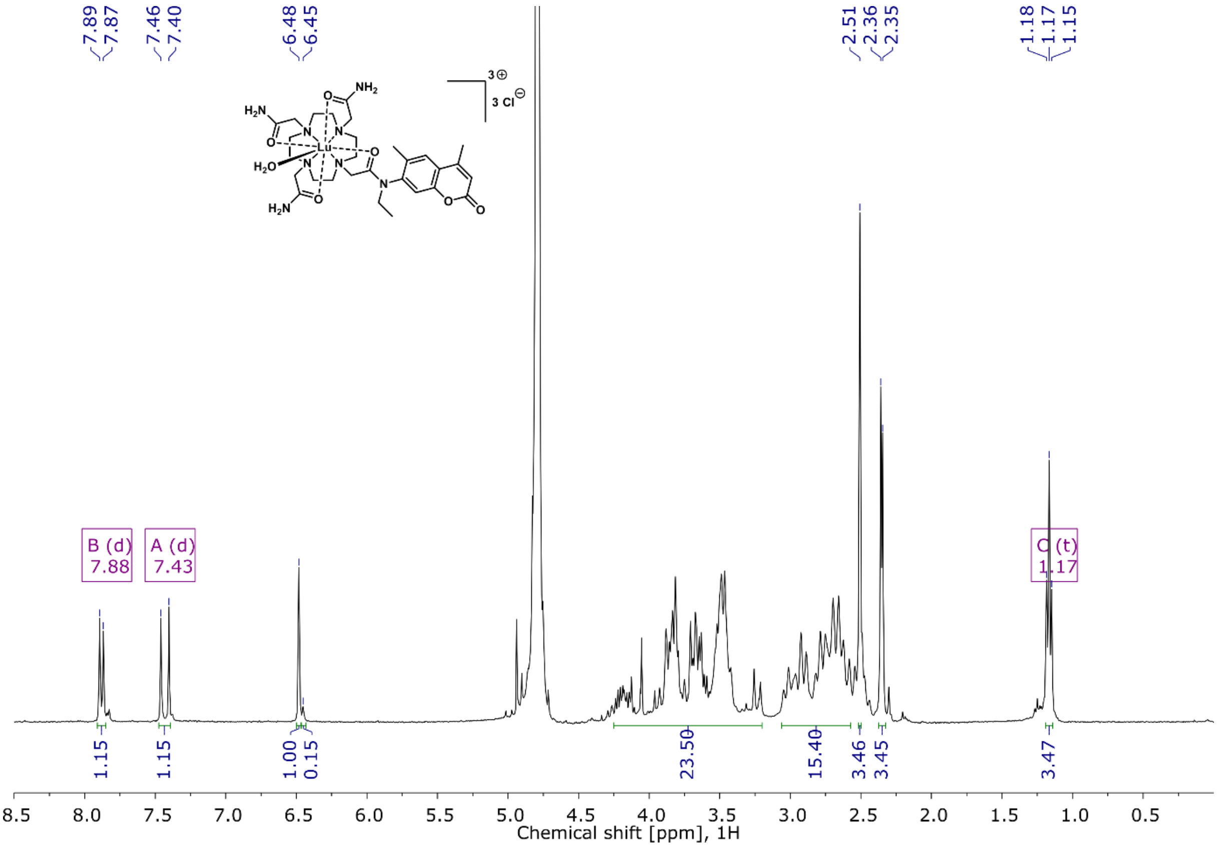

Figure S13. ^{1}H NMR spectrum (400 MHz) of **LuL2d**$^{\mathbf{Cou}}$ measured in D_2O at r.t.

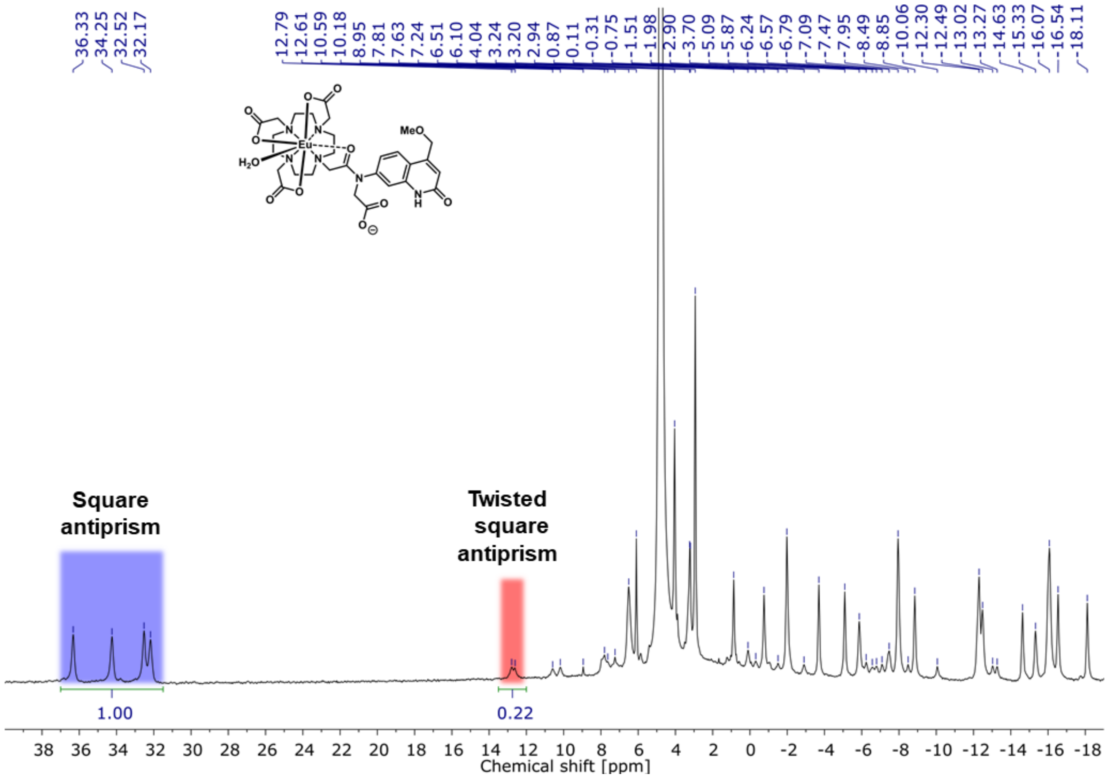

Figure S14. ^{1}H NMR spectrum (400 MHz) of **EuL1a^{Car}** measured in D_2O at 10 °C with the regions corresponding to TSAP and SAP cyclen ring protons in red and blue respectively.

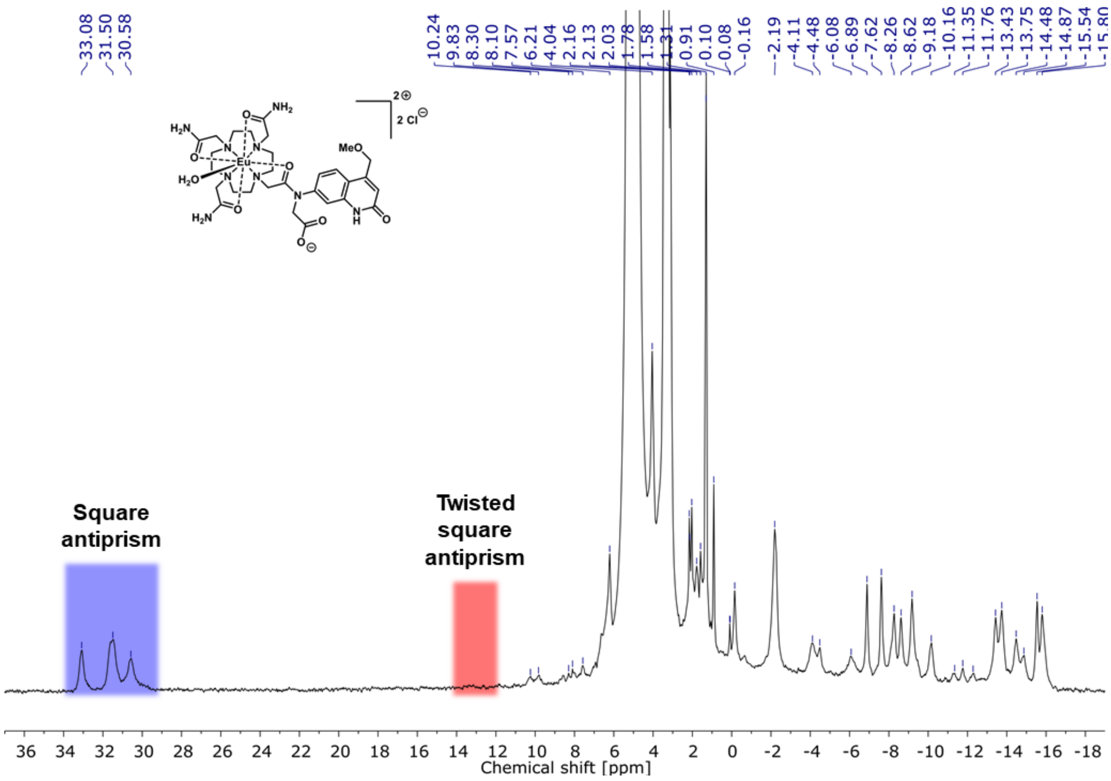

Figure S15. ^{1}H NMR spectrum (400 MHz) of **EuL2a^{Car}** measured in CD_3OD at 0 °C with the regions corresponding to TSAP and SAP cyclen ring protons in red and blue respectively.

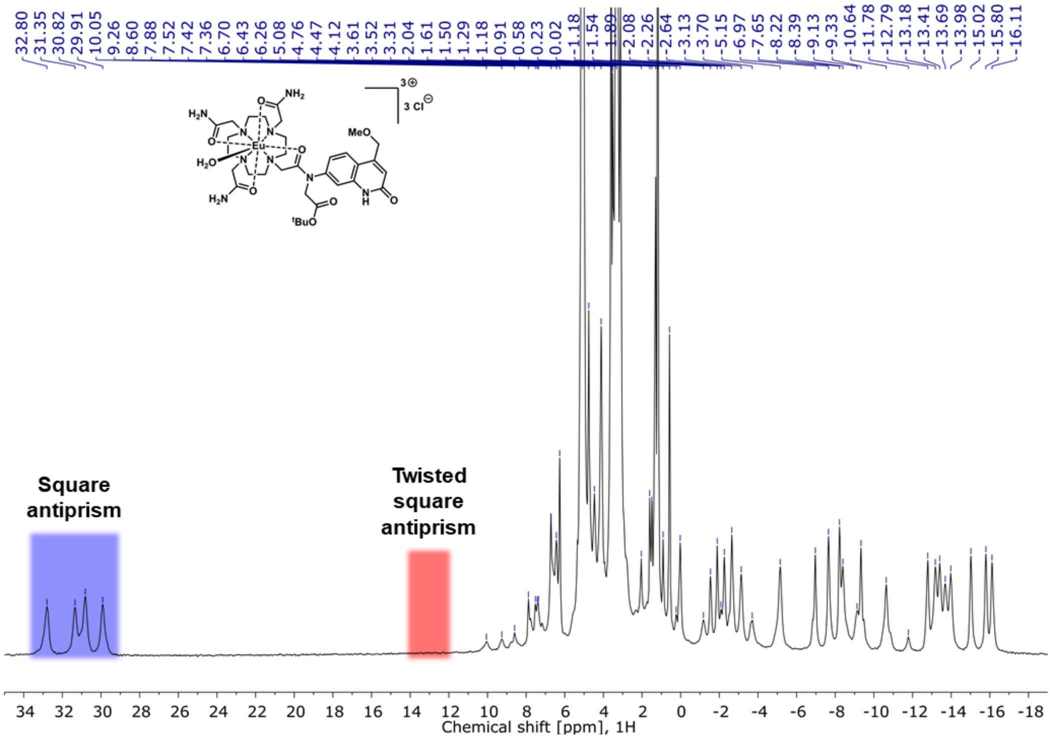

Figure S16. ^{1}H NMR spectrum (400 MHz) of **EuL2c^{Car}** measured in CD_3OD at 0 °C with the regions corresponding to TSAP and SAP cyclen ring protons in red and blue respectively.

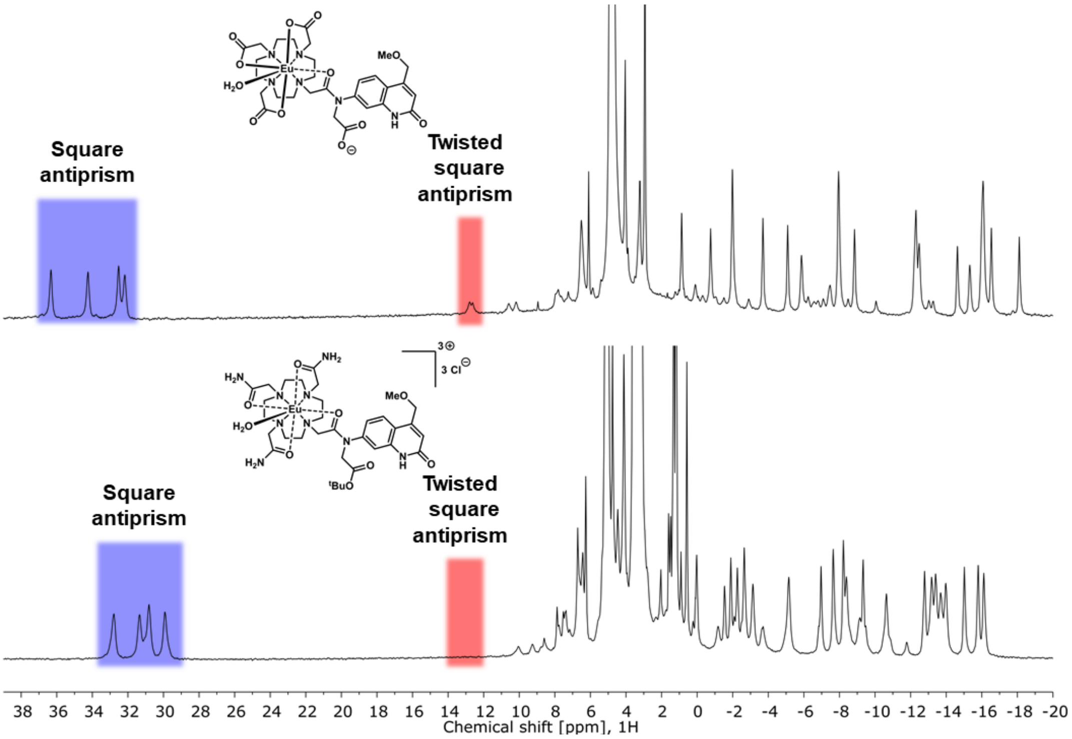

Figure S16. Stacked ^{1}H NMR spectra (400 MHz) of **EuL1a^{Car}** (top, D_2O, 10 °C) and **EuL2c^{Car}** (bottom, CD_3OD, 0°C) with the regions corresponding to TSAP and SAP cyclen ring protons in red and blue respectively.

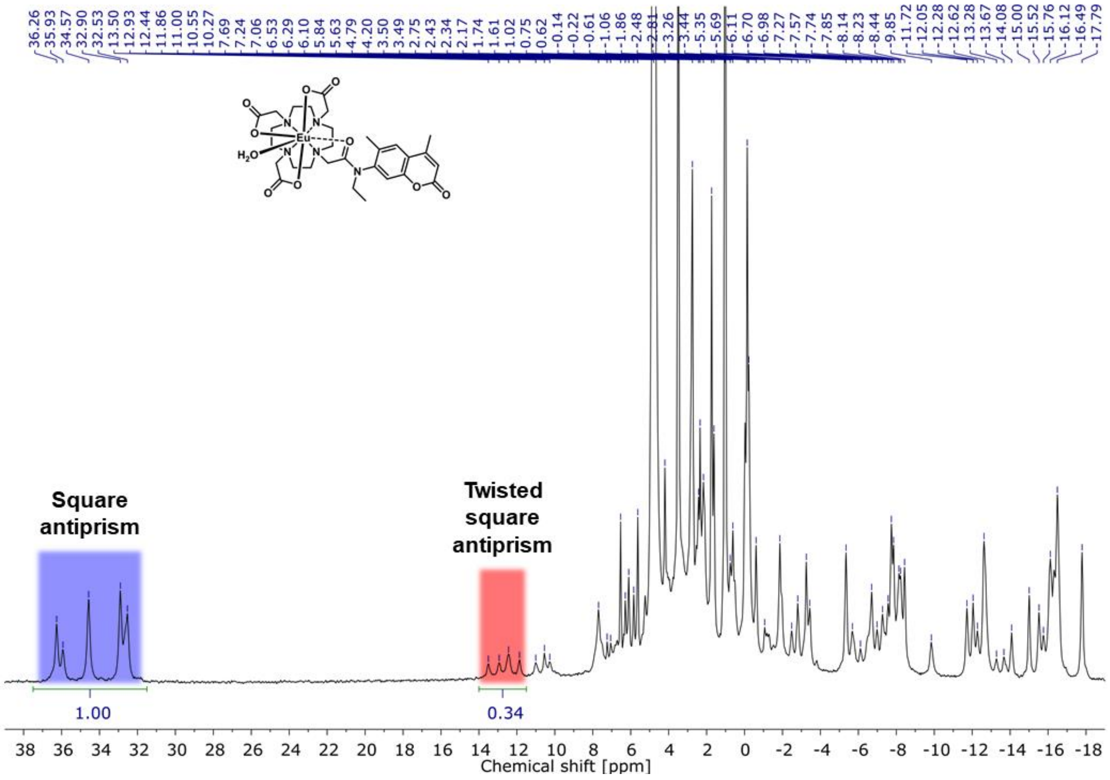

Figure S18. ^{1}H NMR spectrum (400 MHz) of **EuL1d$^{\mathbf{Cou}}$** measured in D_2O at 10 °C with the regions corresponding to TSAP and SAP cyclen ring protons in red and blue respectively.

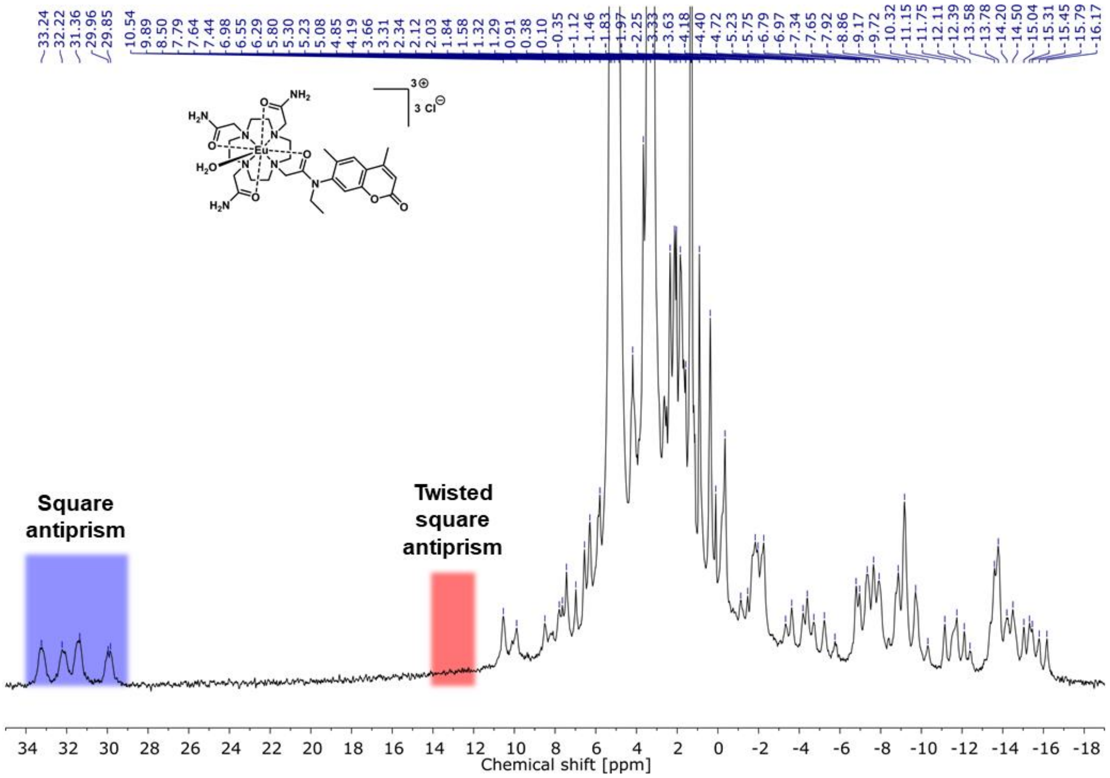

Figure S19. ^{1}H NMR spectrum (400 MHz) of **EuL2d$^{\mathbf{Cou}}$** measured in CD_3OD at 0 °C with the regions corresponding to TSAP and SAP cyclen ring protons in red and blue respectively.

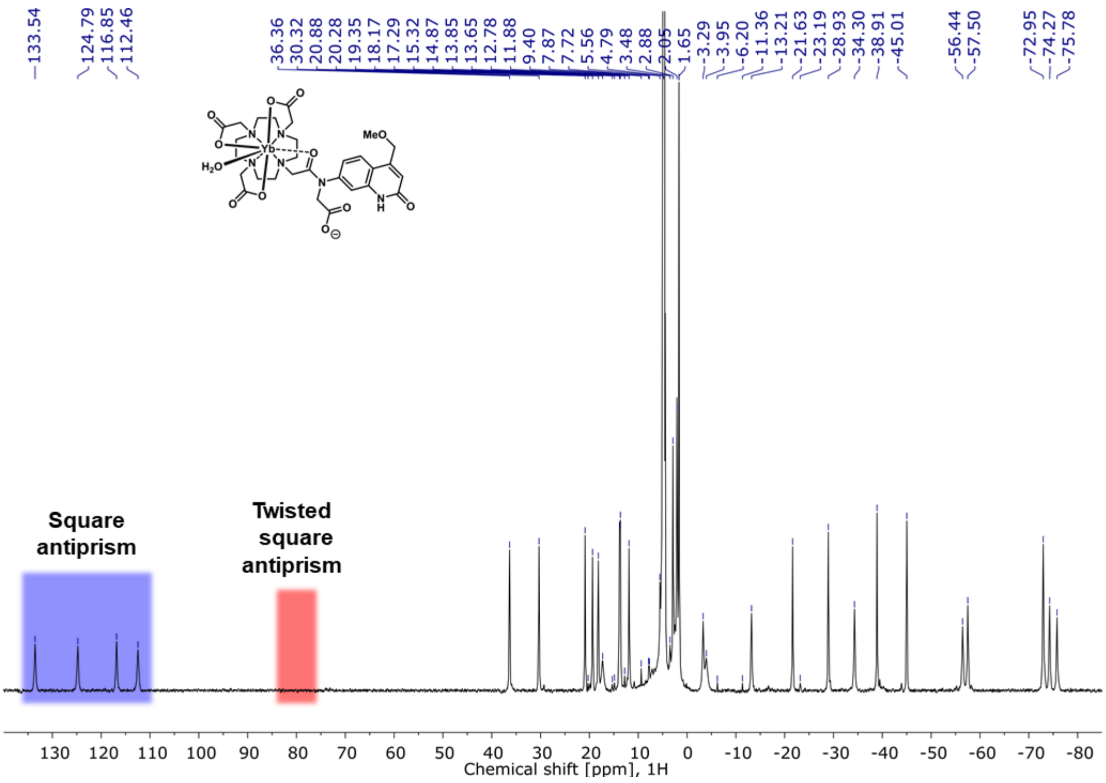

Figure S20. ^{1}H NMR spectrum (400 MHz) of **YbL1a^{Car}** measured in D_2O at r.t. with the regions corresponding to TSAP and SAP cyclen ring protons in red and blue respectively.

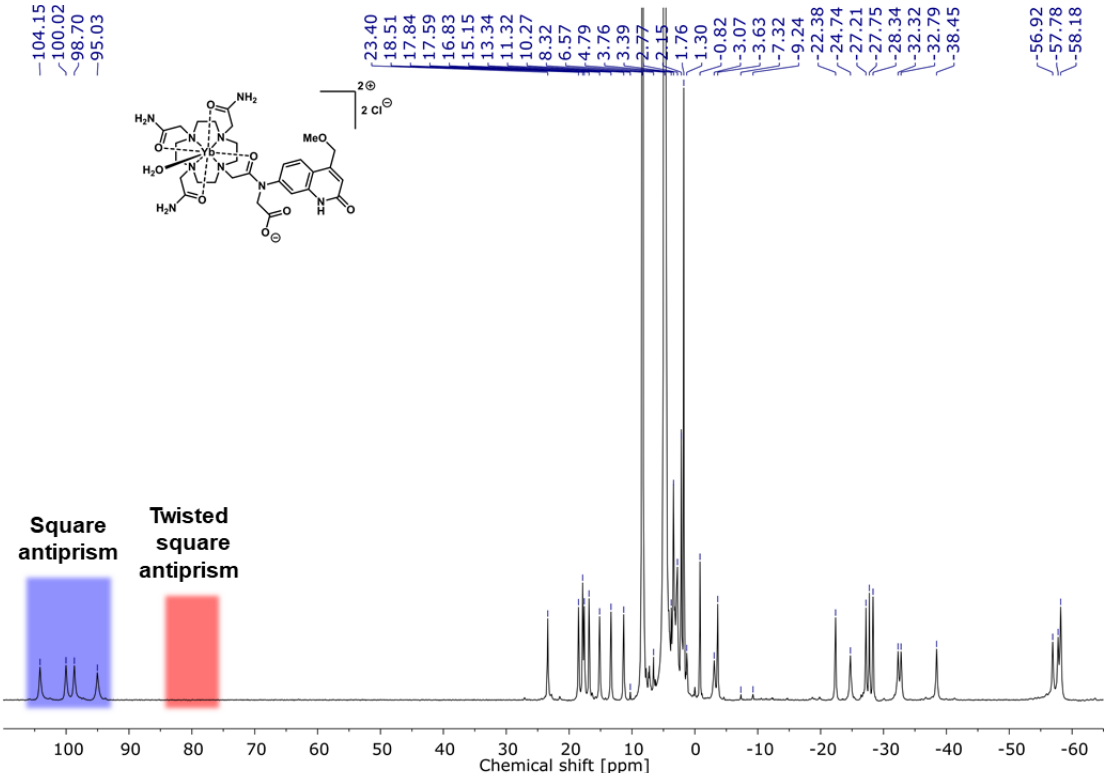

Figure S21. ^{1}H NMR spectrum (400 MHz) of **YbL2a^{Car}** measured in D_2O at r.t. with the regions corresponding to TSAP and SAP cyclen ring protons in red and blue respectively.

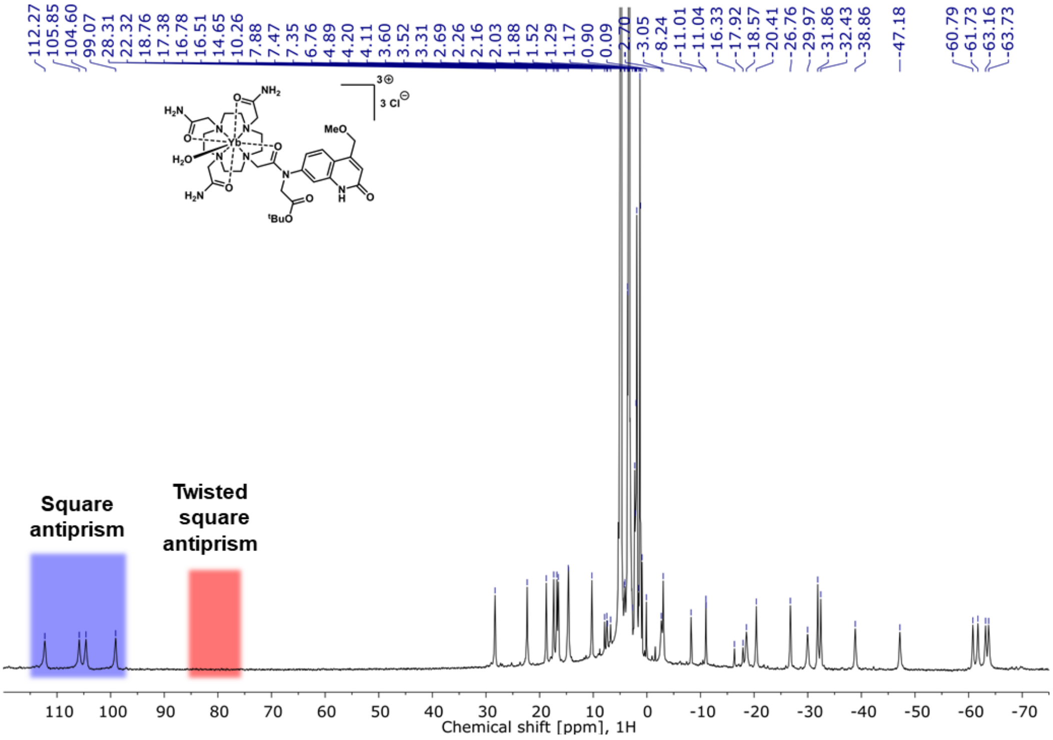

Figure S22. ^{1}H NMR spectrum (400 MHz) of **YbL2c^{Car}** measured in CD_3OD at r.t. with the regions corresponding to TSAP and SAP cyclen ring protons in red and blue respectively.

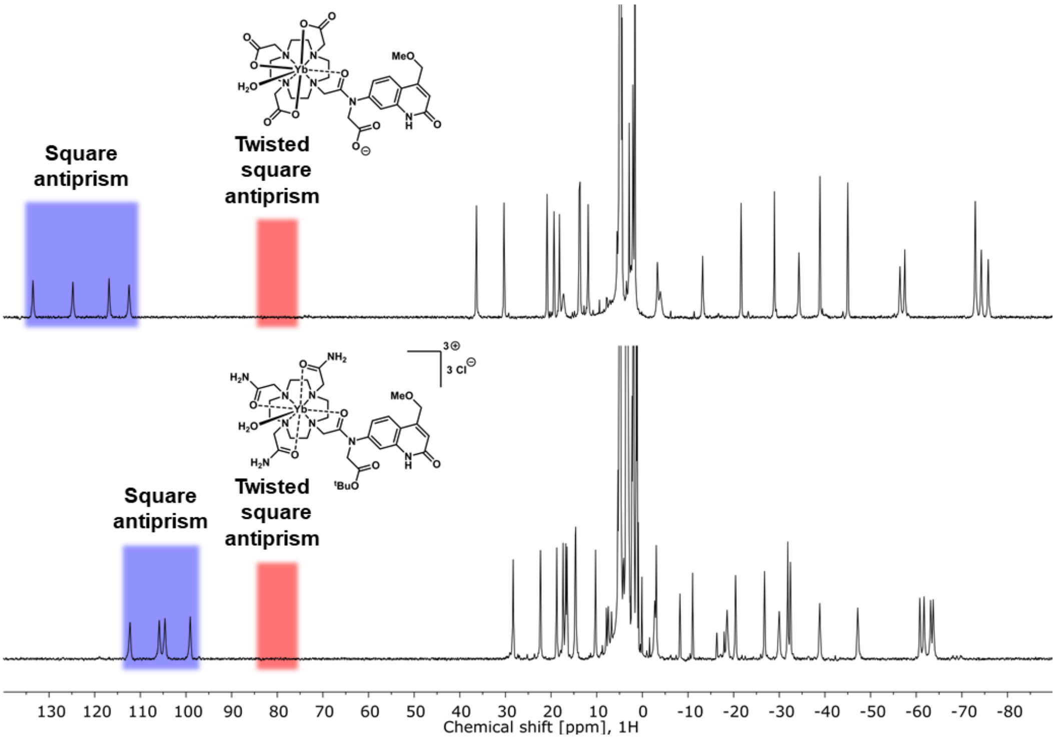

Figure S23. Stacked ^{1}H NMR spectra (400 MHz) of **YbL1a^{Car}** (top, D_2O, r.t.) and **YbL2c^{Car}** (bottom, CD_3OD, r.t.) with the regions corresponding to TSAP and SAP cyclen ring protons in red and blue respectively.

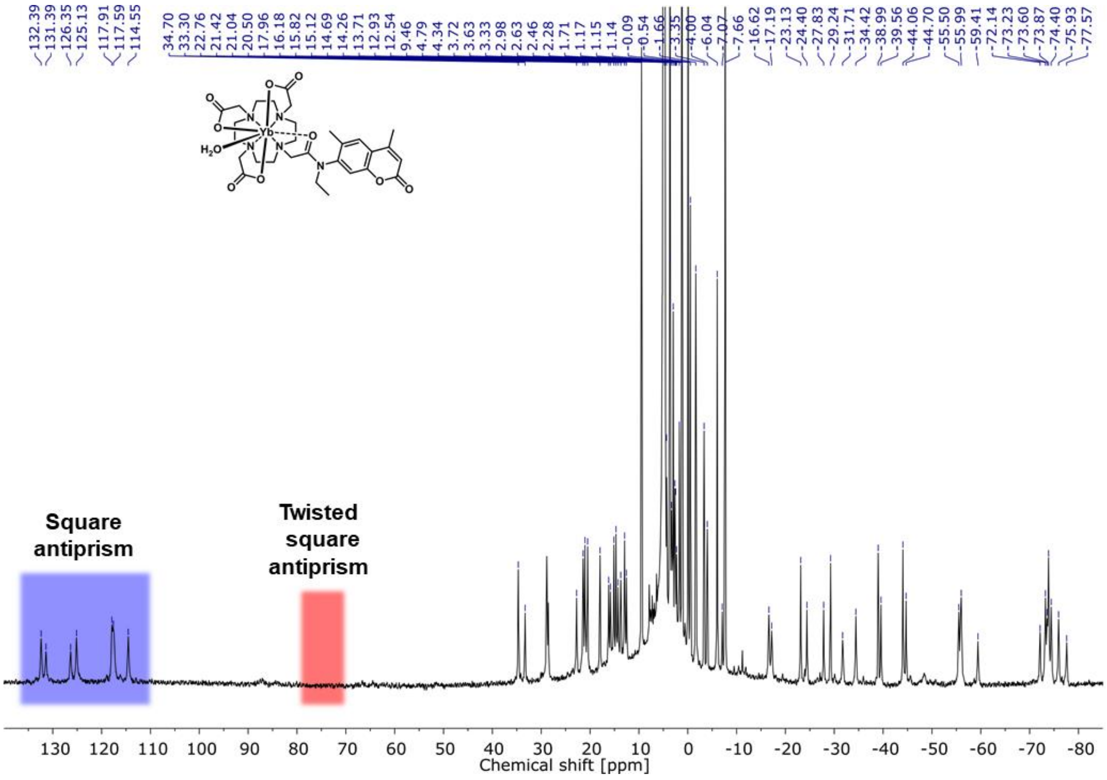

Figure S24. ^{1}H NMR spectrum (400 MHz) of **YbL1d^{Cou}** measured in D_2O at r.t. with the regions corresponding to TSAP and SAP cyclen ring protons in red and blue respectively.

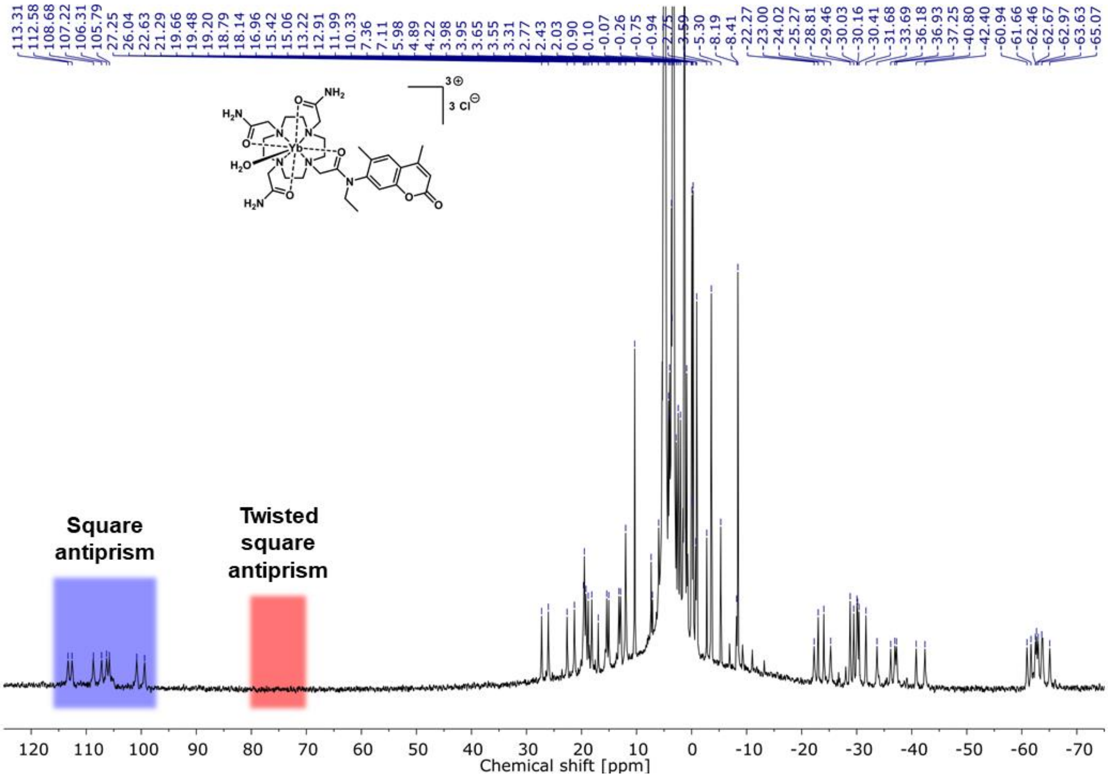

Figure S25. ^{1}H NMR spectrum (400 MHz) of **YbL2d^{Cou}** measured in CD_3OD at r.t. with the regions corresponding to TSAP and SAP cyclen ring protons in red and blue respectively.

Cyclic Voltammetry

In the samples of **EuL1-2d^{Cou}** Eu^{3+} (aq) is also detected in the cyclic voltammograms.

Aqueous solutions

Table S1. pH values of the solutions used in aqueous cyclic voltammetry.

Complex	pH
**$EuCl_3$**	6.53
**EuL1a^{Car}**	6.47
**EuL2a^{Car}** (Cl^- counterions)	6.51
**EuL2c^{Car}** (Cl^- counterions)	6.52
**EuL1d^{Cou}**	6.58
**EuL2d^{Cou}** (Cl^- counterions)	6.53
**EuL2d^{Cou}** (OTf^- counterions)	6.57

Table S2. Cyclic voltammetry of Eu(III) complexes with OTf-counterions. [a]

Compound	$E_{1/2}$ [b]	E_{pa} [b]	E_{pc} [b]	ΔE [b]
**EuL2d^{Cou}**	–634	–536	–732	196

[a] $E_{1/2}$ is a half-wave potential, E_{pa} (E_{pc}) is anodic (cathodic) peak potential, ΔE is peaks separation. [b] Values are in mV vs. NHE. Measured in H_2O (LiCl 0.1 M, pH 6.57) with a sample concentration of 1 mM at a glassy C electrode using a SCE as a reference electrode and a Pt wire counter electrode with a scan rate of 100 mV/s.

Table S3. Comparison of cyclic voltammetry data for secondary and tertiary amide-linked carbostyril Eu(III) complexes. [a]

Compound	$E_{1/2}$ [b]	E_{pa} [b]	E_{pc} [b]	ΔE [b]
**EuL1a^{Car}**	–948	–766	–1131	365
**EuL1b^{Car}** [c]	–839	–771	–908	137
**EuL2a^{Car}**	–612	–437	–787	350
**EuL2b^{Car}** [c]	–554	–472	–643	171

[a] $E_{1/2}$ is a half-wave potential, E_{pa} (E_{pc}) is anodic (cathodic) peak potential, ΔE is peaks separation. [b] Values are in mV vs. NHE. Measured in H_2O (LiCl 0.1 M, pH 6.3–6.7) with a sample concentration of 1 mM at a glassy C electrode using a SCE as a reference electrode and a Pt wire counter electrode with a scan rate of 100 mV/s. [c] Data from [9].

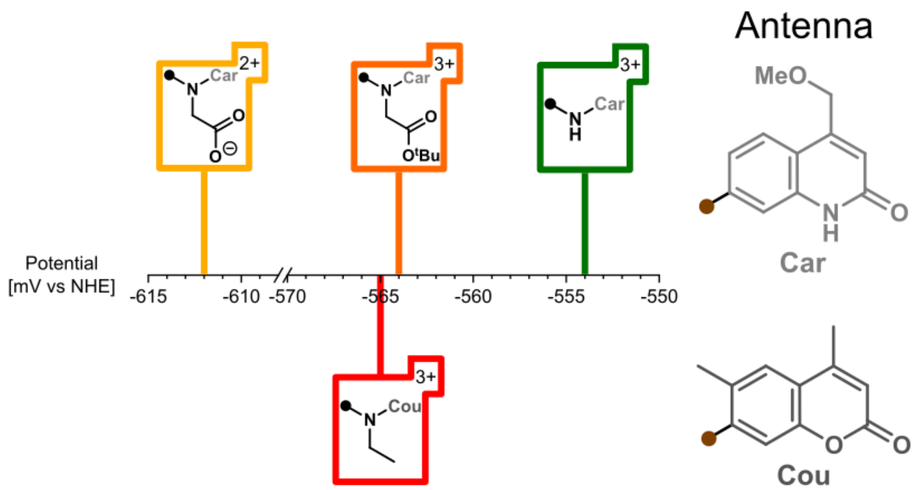

Figure S26. Tertiary amide linker structure vs apparent Eu(III)/Eu(II) reduction potential of **EuL2** complexes. **EuL2a^{Car}** is in dark yellow, **EuL2c^{Car}** is in orange **EuL2b^{Car}** is in dark green and **EuL2d^{Cou}** is in red.

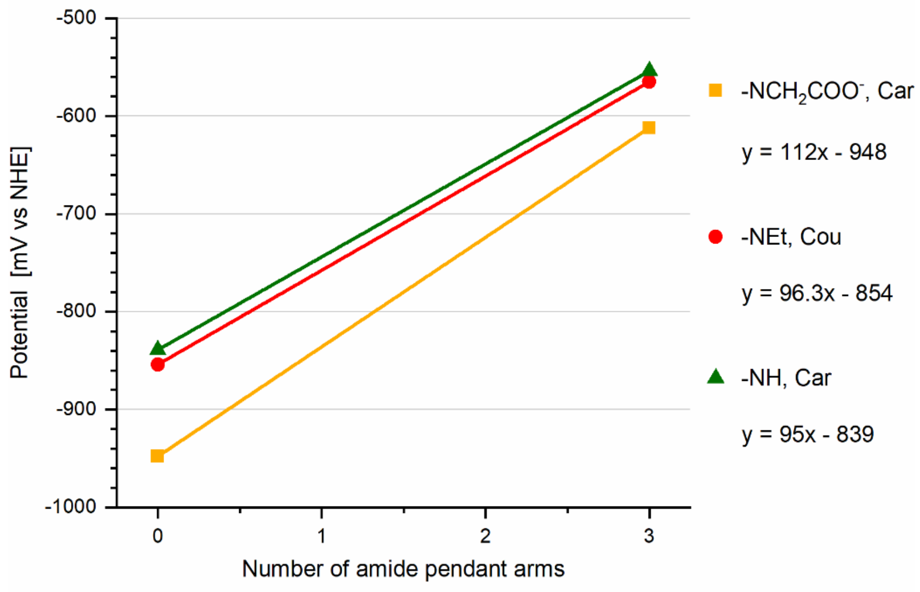

Figure S27. Number of amide pendant arms vs apparent Eu(III)/Eu(II) reduction potential of **EuL** complexes. **EuL1-2a^{Car}** are in dark yellow, **EuL1-2b^{Car}** are in dark green and **EuL1-2d^{Cou}** are in red.

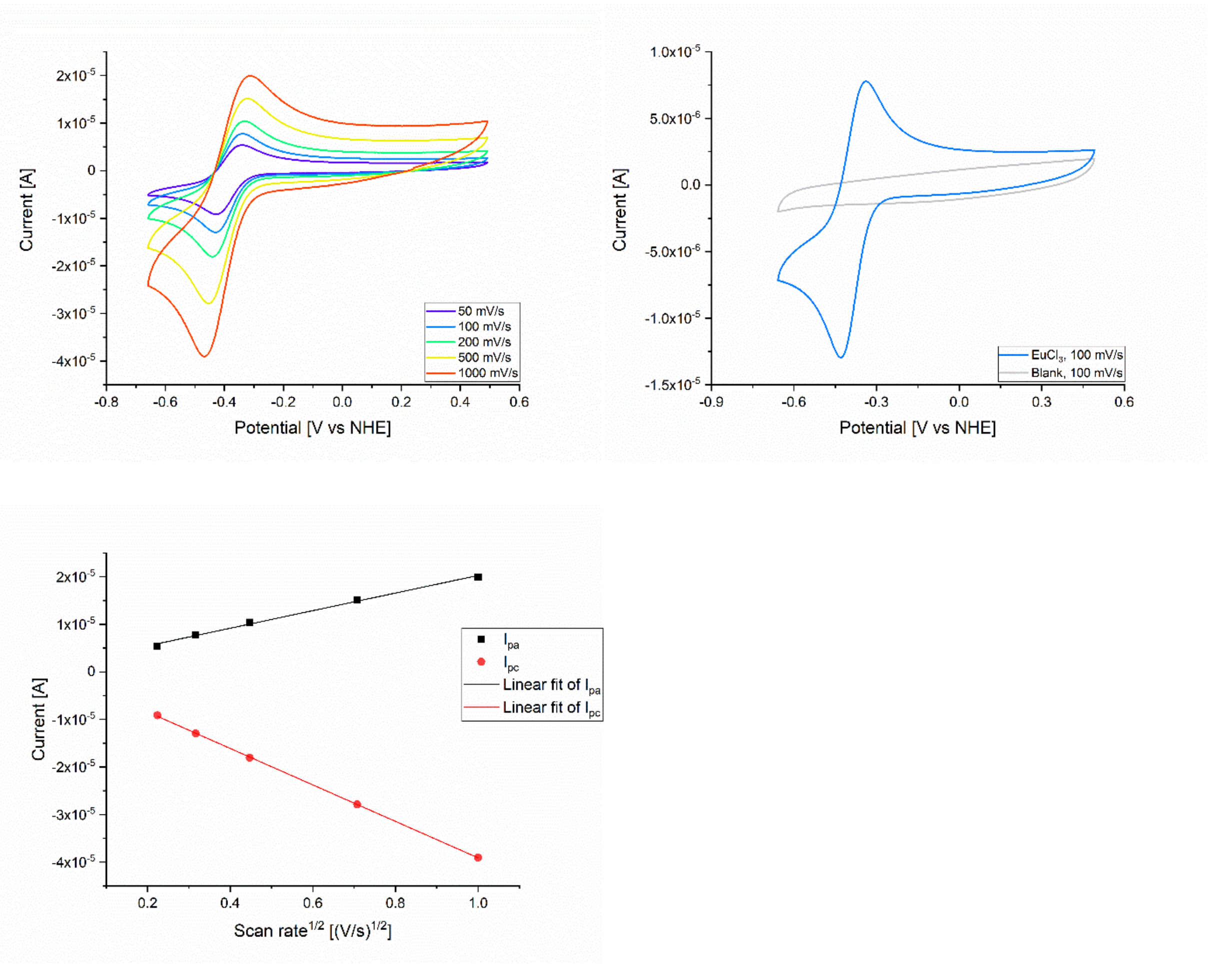

Figure S28. Cyclic voltammograms at various scan rates for **$EuCl_3$** and plot of I_{pa} and I_{pc} vs. square root of scan rate.

Table S4. Values for linear fit of I_{pa} and I_{pc} *vs*. square root of scan rate for $EuCl_3$.

**Equation: y = a*x + b**	**I_{pa}**	**I_{pc}**
Slope (a)	$1.85 \cdot 10^{-5} \pm 7.21 \cdot 10^{-7}$	$-3.84 \cdot 10^{-5} \pm 2.26 \cdot 10^{-7}$
Intercept (b)	$1.78 \cdot 10^{-6} \pm 4.39 \cdot 10^{-7}$	$-7.59 \cdot 10^{-7} \pm 1.38 \cdot 10^{-7}$
R-Square (COD)	0.99546	0.9999

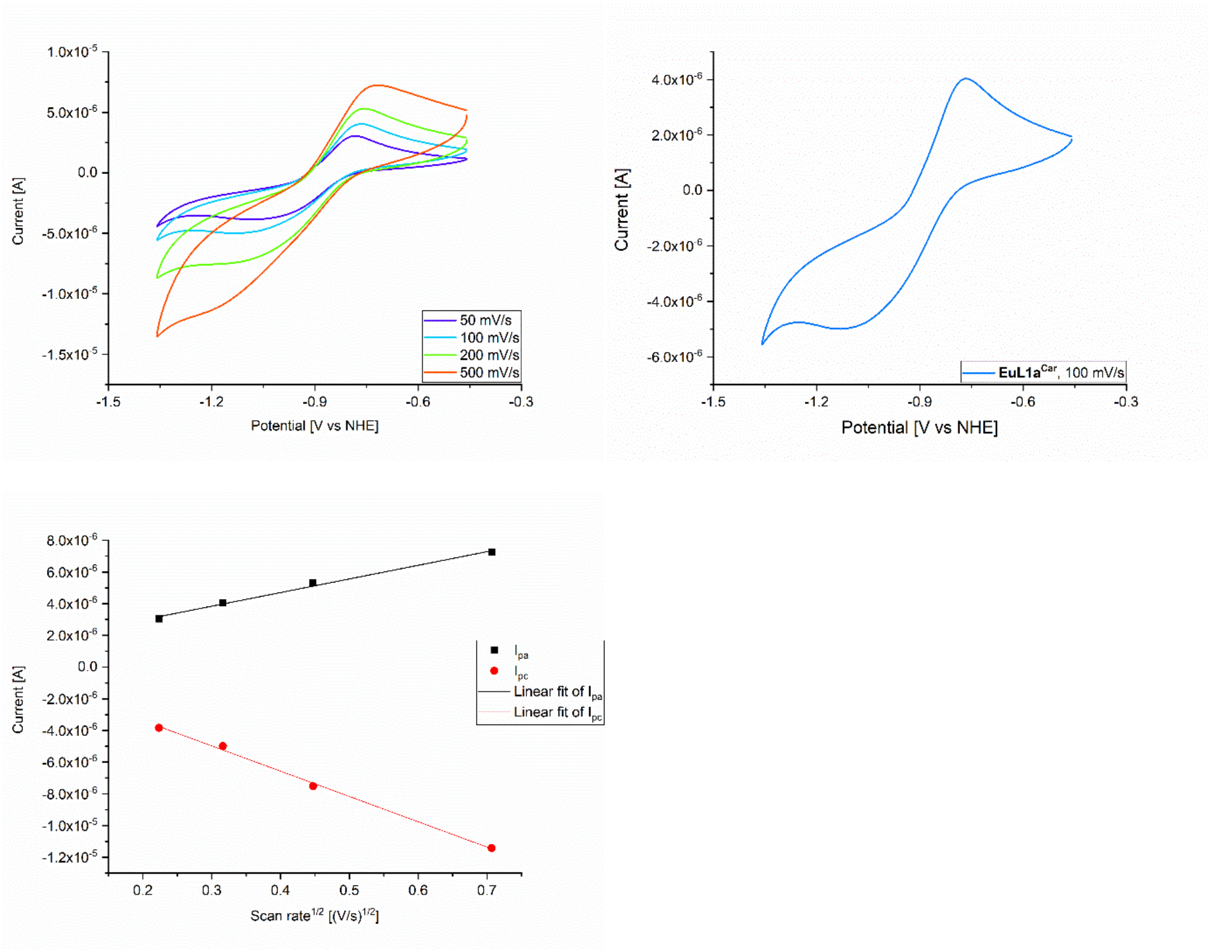

Figure S29. Cyclic voltammograms at various scan rates for **EuL1a$^{\mathbf{Car}}$** and plot of I_{pa} and I_{pc} vs. square root of scan rate.

Table S5. Values for linear fit of I_{pa} and I_{pc} *vs*. square root of scan rate for **EuL1a$^{\mathbf{Car}}$**.

**Equation: y = a*x + b**	$\mathbf{I_{pa}}$	$\mathbf{I_{pc}}$
Slope (a)	$0.86 \cdot 10^{-5} \pm 5.16 \cdot 10^{-7}$	$-1.60 \cdot 10^{-5} \pm 6.33 \cdot 10^{-7}$
Intercept (b)	$1.26 \cdot 10^{-6} \pm 2.38 \cdot 10^{-7}$	$-1.82 \cdot 10^{-7} \pm 2.92 \cdot 10^{-7}$
R-Square (COD)	0.99287	0.99687

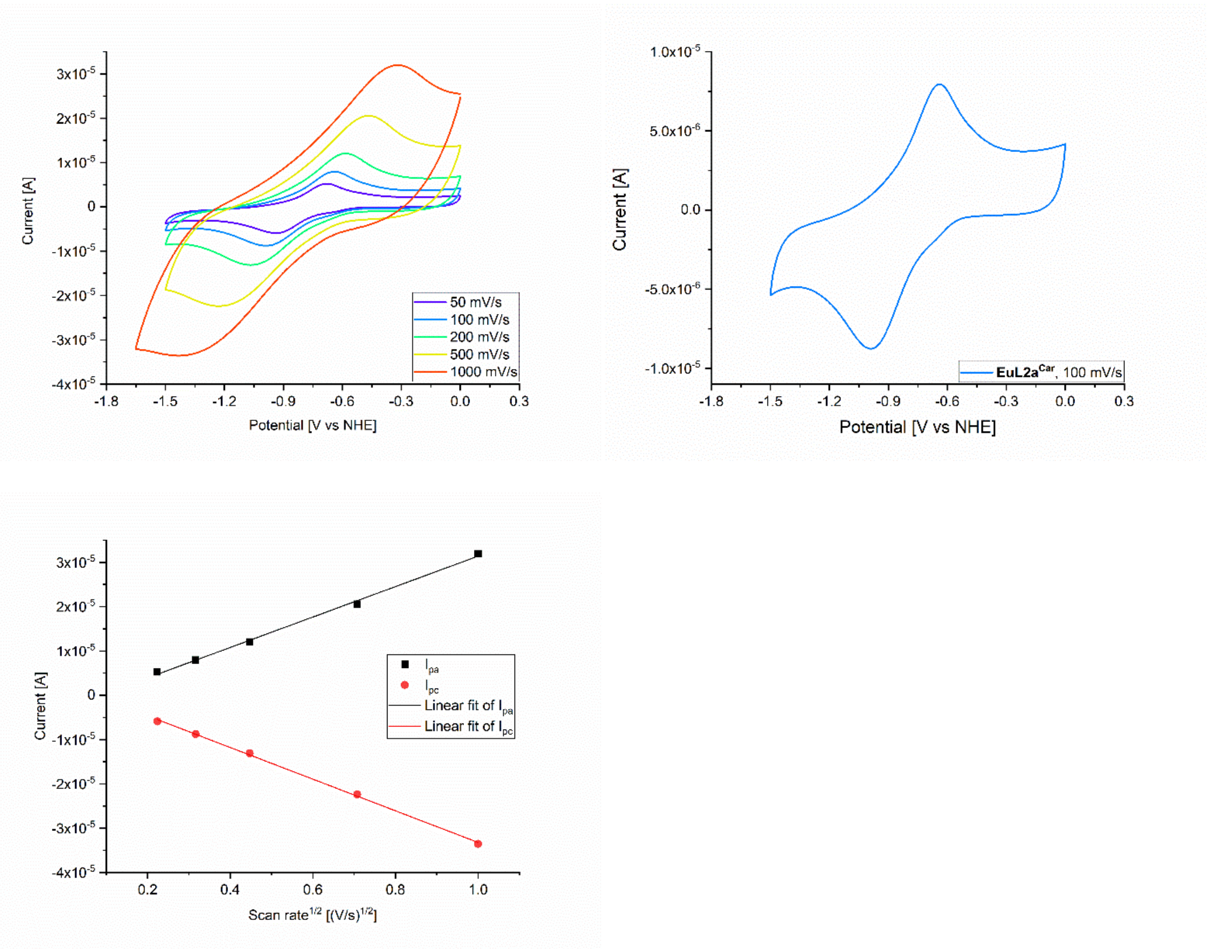

Figure S30. Cyclic voltammograms at various scan rates for **EuL2a$^{\mathbf{Car}}$** and plot of I_{pa} and I_{pc} vs. square root of scan rate.

Table S6. Values for linear fit of I_{pa} and I_{pc} *vs*. square root of scan rate for **EuL2a$^{\mathbf{Car}}$**.

**Equation: y = a*x + b**	$\mathbf{I_{pa}}$	$\mathbf{I_{pc}}$
Slope (a)	$3.43{\cdot}10^{-5} \pm 1.06{\cdot}10^{-6}$	$-3.57{\cdot}10^{-5} \pm 7.07{\cdot}10^{-7}$
Intercept (b)	$-2.95{\cdot}10^{-6} \pm 6.47{\cdot}10^{-7}$	$2.50{\cdot}10^{-6} \pm 4.30{\cdot}10^{-7}$
R-Square (COD)	0.99713	0.99883

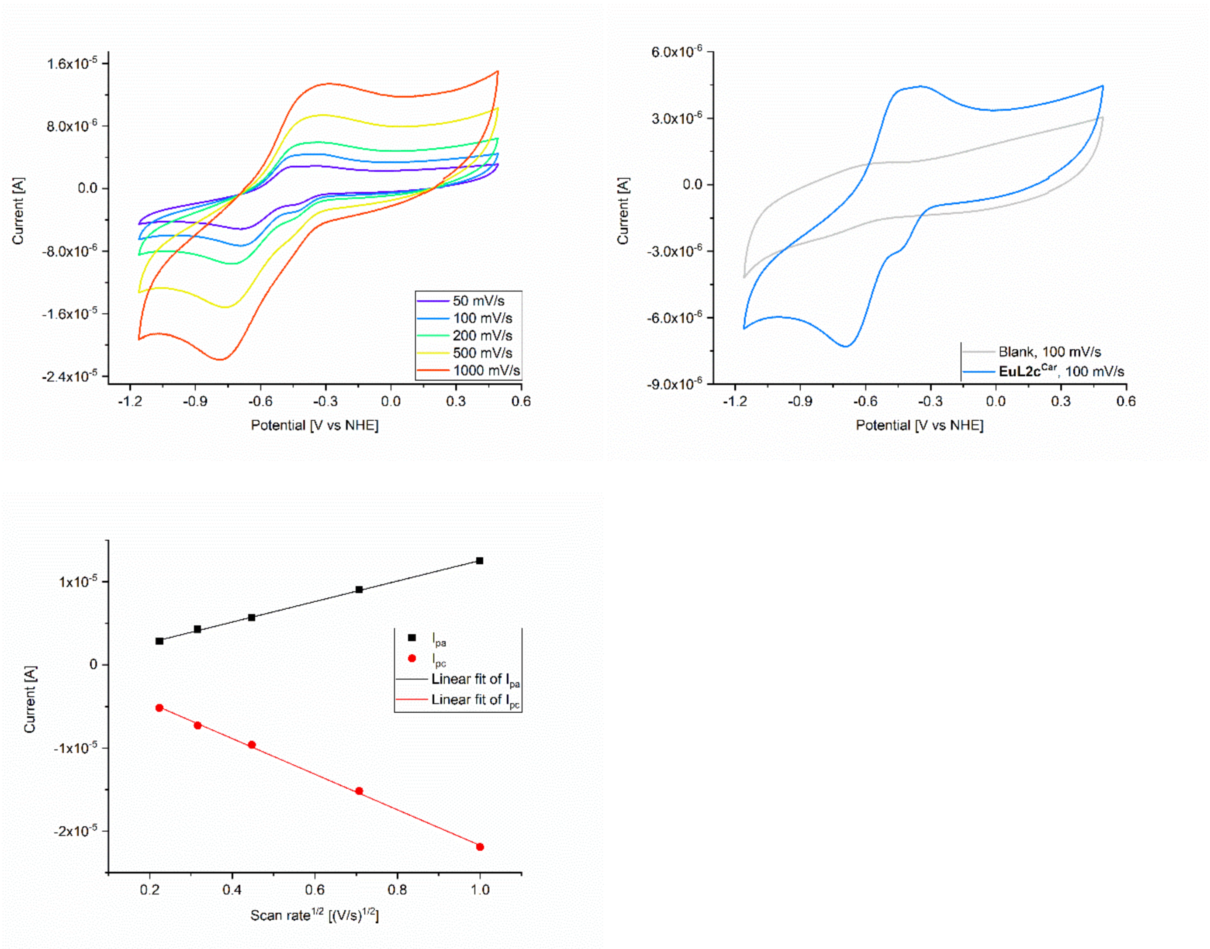

Figure S31. Cyclic voltammograms at various scan rates for **EuL2c$^{\mathbf{Car}}$** and plot of I_{pa} and I_{pc} vs. square root of scan rate.

Table S7. Values for linear fit of I_{pa} and I_{pc} *vs*. square root of scan rate for **EuL2c$^{\mathbf{Car}}$**.

**Equation: y = a*x + b**	$\mathbf{I_{pa}}$	$\mathbf{I_{pc}}$
Slope (a)	$1.23{\cdot}10^{-5} \pm 2.18{\cdot}10^{-7}$	$-2.14{\cdot}10^{-5} \pm 4.38{\cdot}10^{-7}$
Intercept (b)	$2.20{\cdot}10^{-7} \pm 1.33{\cdot}10^{-7}$	$-3.06{\cdot}10^{-7} \pm 2.66{\cdot}10^{-7}$
R-Square (COD)	0.99906	0.99875

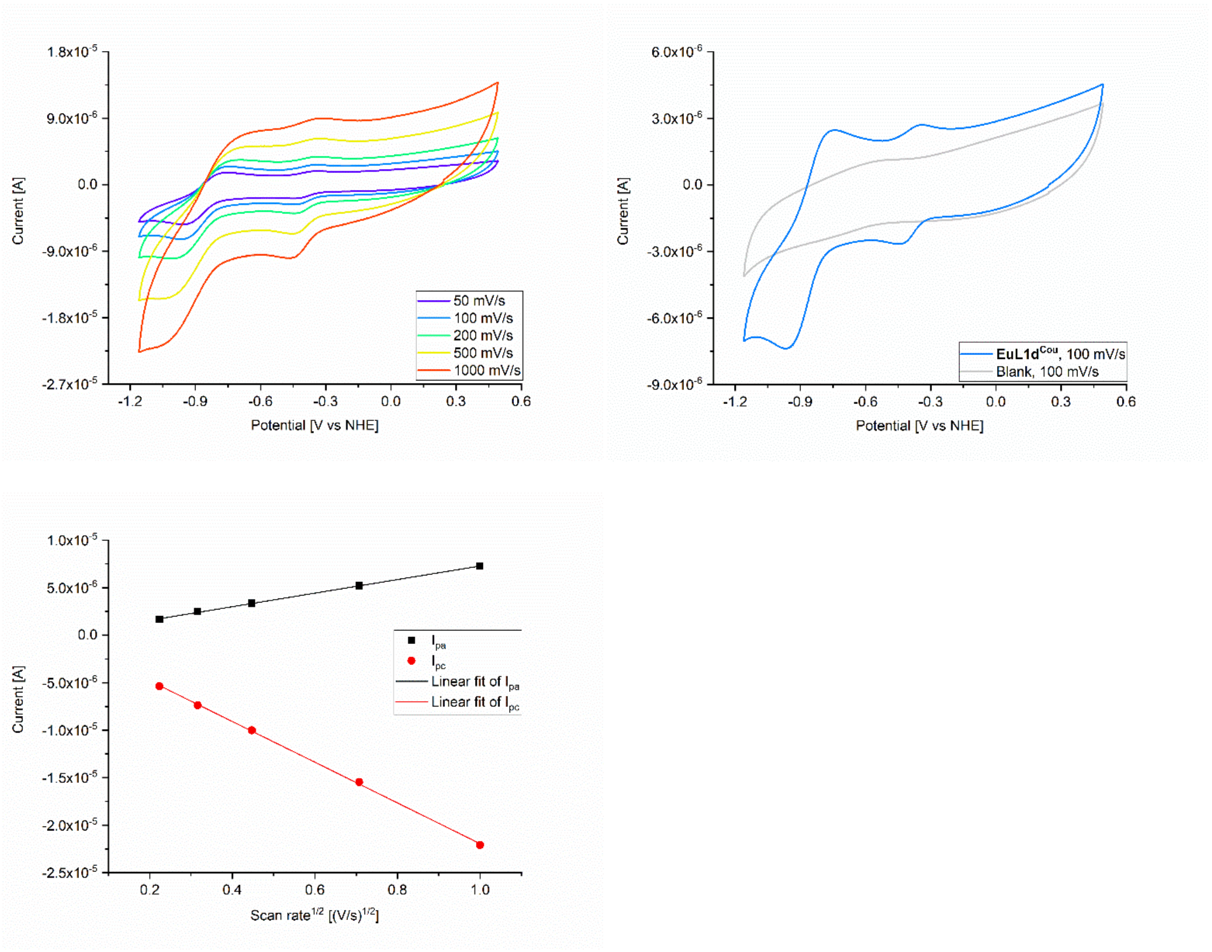

Figure S32. Cyclic voltammograms at various scan rates for **EuL1d^{Cou}** and plot of I_{pa} and I_{pc} vs. square root of scan rate.

Table S8. Values for linear fit of I_{pa} and I_{pc} *vs*. square root of scan rate for **EuL1d^{Cou}**.

**Equation: y = a*x + b**	**I_{pa}**	**I_{pc}**
Slope (a)	$0.72 \cdot 10^{-5} \pm 1.12 \cdot 10^{-7}$	$-2.14 \cdot 10^{-5} \pm 2.62 \cdot 10^{-7}$
Intercept (b)	$1.40 \cdot 10^{-7} \pm 6.80 \cdot 10^{-7}$	$-5.13 \cdot 10^{-7} \pm 1.60 \cdot 10^{-7}$
R-Square (COD)	0.99927	0.99955

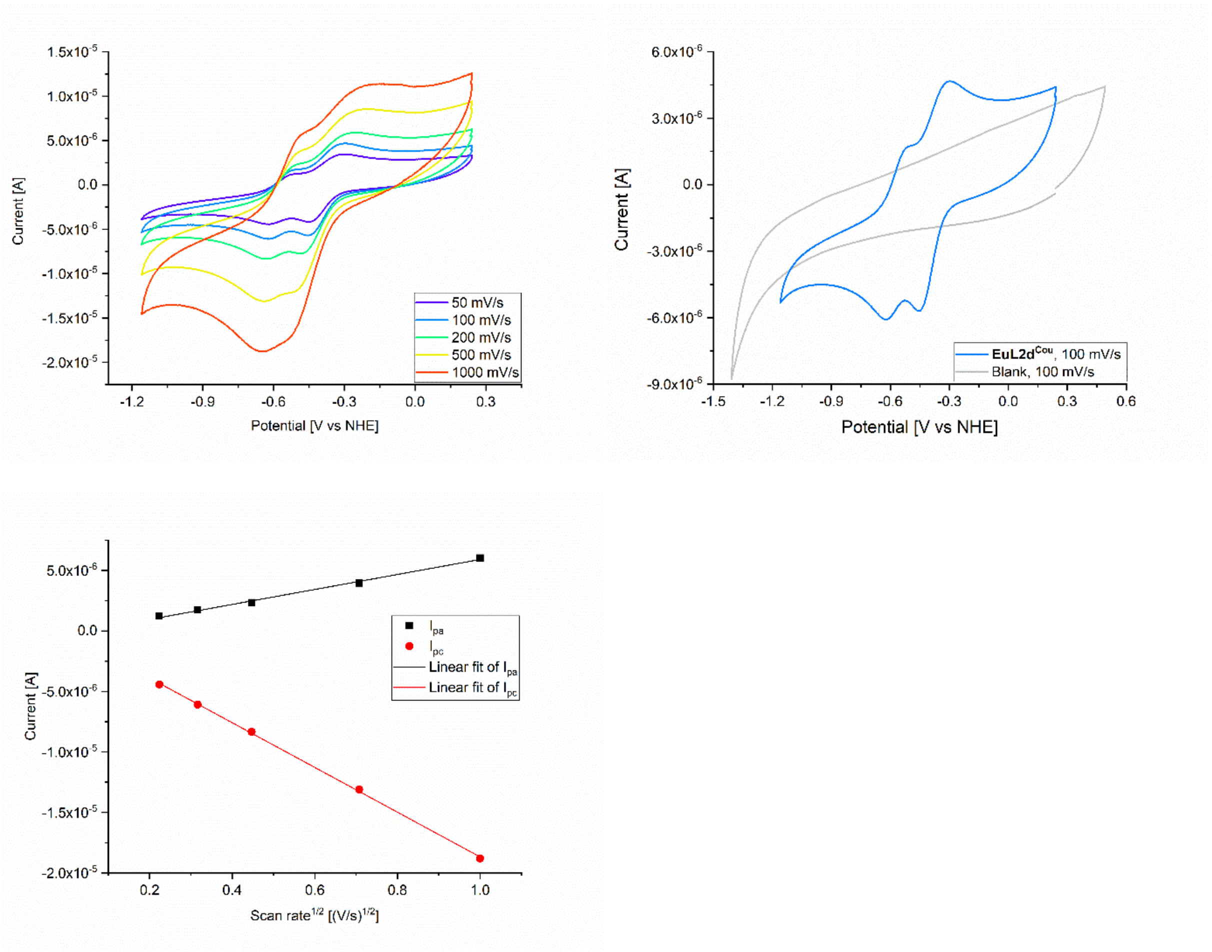

Figure S33. Cyclic voltammograms at various scan rates for **EuL2d^{Cou}** and plot of I_{pa} and I_{pc} vs. square root of scan rate.

Table S9. Values for linear fit of I_{pa} and I_{pc} *vs*. square root of scan rate for **EuL2d^{Cou}**.

**Equation: y = a*x + b**	**I_{pa}**	**I_{pc}**
Slope (a)	$0.62 \cdot 10^{-5} \pm 2.43 \cdot 10^{-7}$	$-1.85 \cdot 10^{-5} \pm 2.34 \cdot 10^{-7}$
Intercept (b)	$-2.86 \cdot 10^{-7} \pm 1.48 \cdot 10^{-7}$	$-1.92 \cdot 10^{-7} \pm 1.42 \cdot 10^{-7}$
R-Square (COD)	0.99542	0.99952

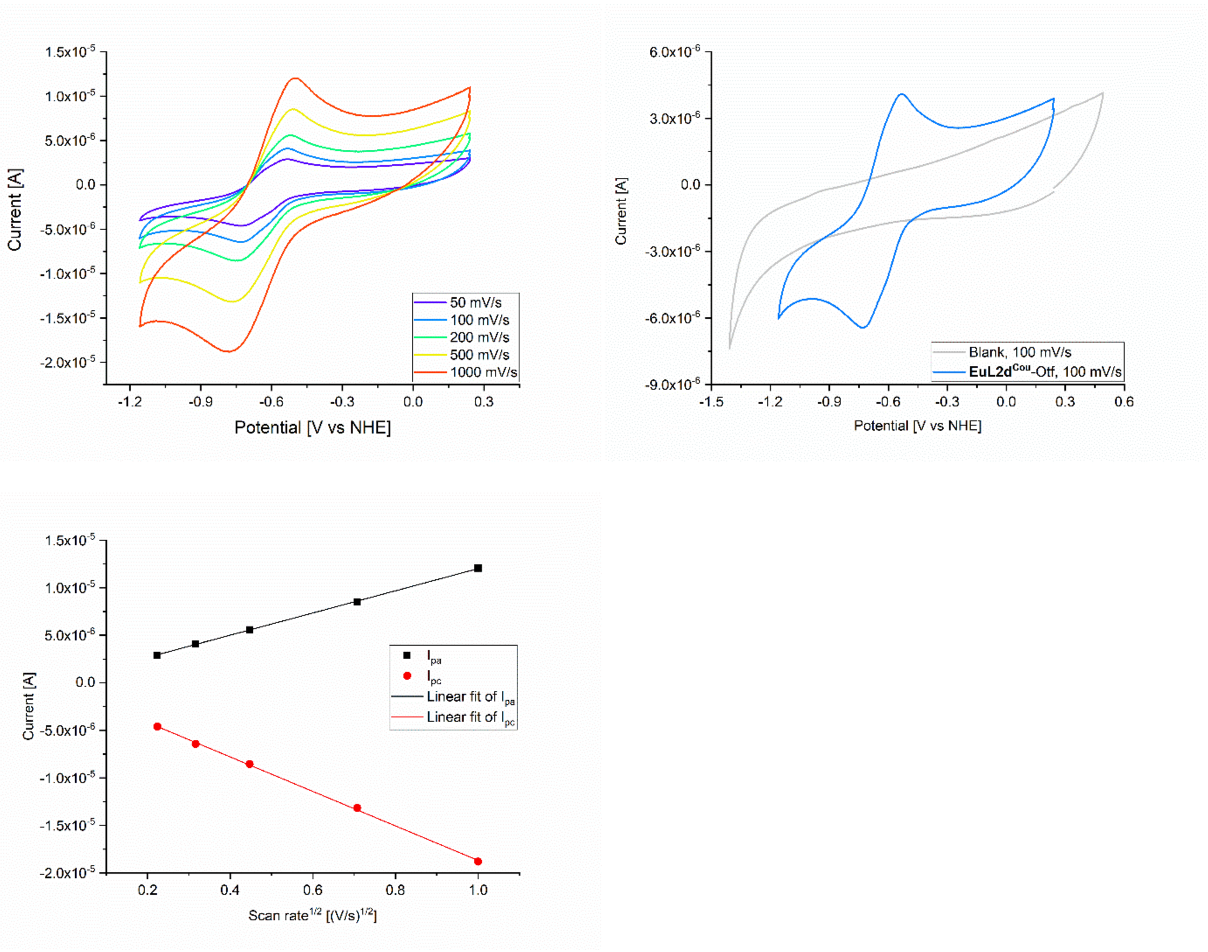

Figure S34. Cyclic voltammograms at various scan rates for **EuL2d**$^{\mathbf{Cou}}$-OTf and plot of I_{pa} and I_{pc} vs. square root of scan rate.

Table S10. Values for linear fit of I_{pa} and I_{pc} *vs*. square root of scan rate for **EuL2d**$^{\mathbf{Cou}}$-OTf.

**Equation: y = a*x + b**	$\mathbf{I_{pa}}$	$\mathbf{I_{pc}}$
Slope (a)	$1.17 \cdot 10^{-5} \pm 1.16 \cdot 10^{-7}$	$-1.81 \cdot 10^{-5} \pm 2.77 \cdot 10^{-7}$
Intercept (b)	$3.41 \cdot 10^{-7} \pm 7.08 \cdot 10^{-8}$	$-5.41 \cdot 10^{-7} \pm 1.68 \cdot 10^{-7}$
R-Square (COD)	0.9997	0.9993

Non-aqueous (DMF) solutions

Table S11. Cyclic voltammetry of Eu(III) complexes in non-aqueous (DMF) media.

Compound	$E_{1/2}$ [a]	E_{pa} [a]	E_{pc} [a]	ΔE [a]
$Eu(OTf)_3$	–433	–349	–518	169
**EuL1a^{Car}**	–1074	–948	–1200	252
**EuL1b^{Car}**	–1118	–1028	–1208	180
**EuL2a^{Car}** (Cl-counterions)	–508	–437	–579	142
**EuL2b^{Car}** (OTf-counterions)	–501	–460	–542	82
**EuL1d^{Cou}**	–1011	–881	–1141	260
**EuL2d^{Cou}** (OTf-counterions)	–750	–711	–789	78

$E_{1/2}$ is a half-wave potential, E_{pa} (E_{pc}) is anodic (cathodic) peak potential, ΔE is peaks separation. [a] Values are in mV vs. NHE. Measured in DMF ($TBAPF_6$ 0.1 M) with a sample concentration of 1 mM at a glassy carbon electrode using a SCE as a reference electrode and a Pt wire counter electrode with a scan rate of 100 mV/s. Ferrocene was added to each sample at the end of experiment and the potentials were adjusted according to reported values [3].

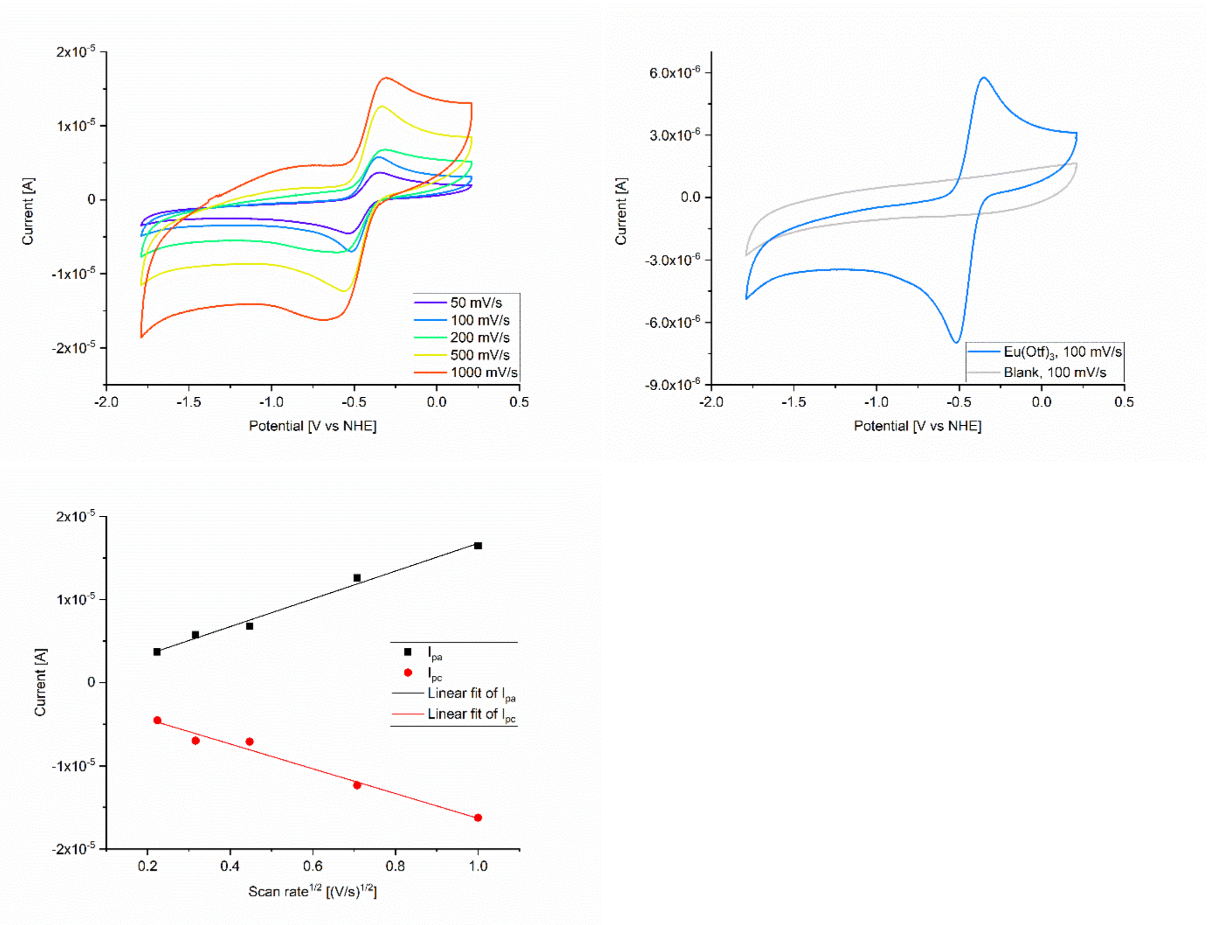

Figure S35. Cyclic voltammograms at various scan rates for $Eu(OTf)_3$ in DMF and plot of I_{pa} and I_{pc} vs. square root of scan rate.

Table S12. Values for linear fit of I_{pa} and I_{pc} *vs*. square root of scan rate for $Eu(OTf)_3$ in DMF.

**Equation: y = a*x + b**	I_{pa}	I_{pc}
Slope (a)	$1.67{\cdot}10^{-5} \pm 1.08{\cdot}10^{-6}$	$-1.49{\cdot}10^{-5} \pm 1.26{\cdot}10^{-6}$
Intercept (b)	$5.95{\cdot}10^{-8} \pm 6.55{\cdot}10^{-7}$	$-1.41{\cdot}10^{-6} \pm 7.65{\cdot}10^{-7}$
R-Square (COD)	0.98769	0.97909

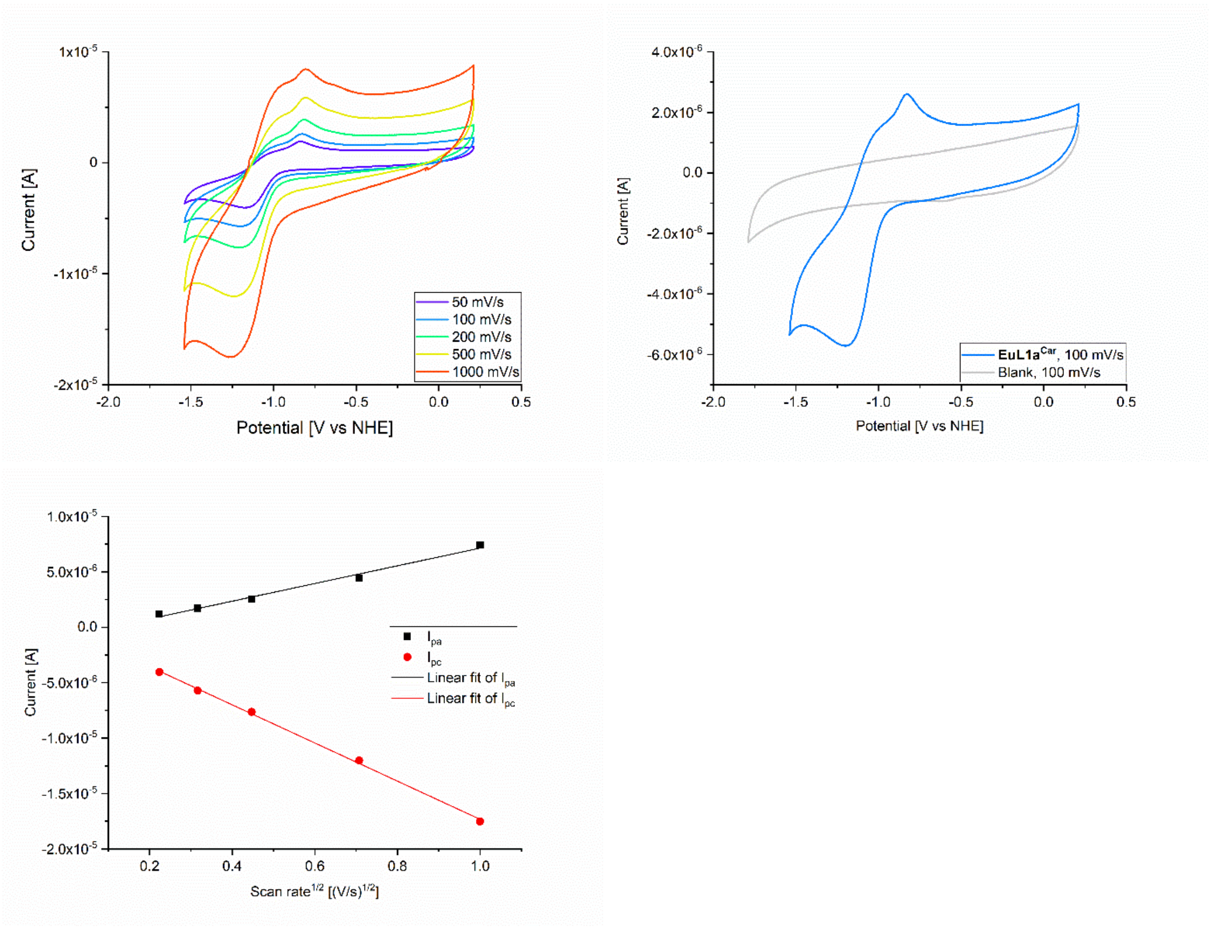

Figure S36. Cyclic voltammograms at various scan rates for **EuL1a**$^{\mathbf{Car}}$ in DMF and plot of I_{pa} and I_{pc} vs. square root of scan rate.

Table S13. Values for linear fit of I_{pa} and I_{pc} *vs*. square root of scan rate for **EuL1a**$^{\mathbf{Car}}$ in DMF.

**Equation: y = a*x + b**	$\mathbf{I_{pa}}$	$\mathbf{I_{pc}}$
Slope (a)	$0.80{\cdot}10^{-5} \pm 5.23{\cdot}10^{-7}$	$-1.72{\cdot}10^{-5} \pm 3.71{\cdot}10^{-6}$
Intercept (b)	$-8.43{\cdot}10^{-7} \pm 3.18{\cdot}10^{-7}$	$-1.12{\cdot}10^{-6} \pm 2.26{\cdot}10^{-7}$
R-Square (COD)	0.98732	0.99861

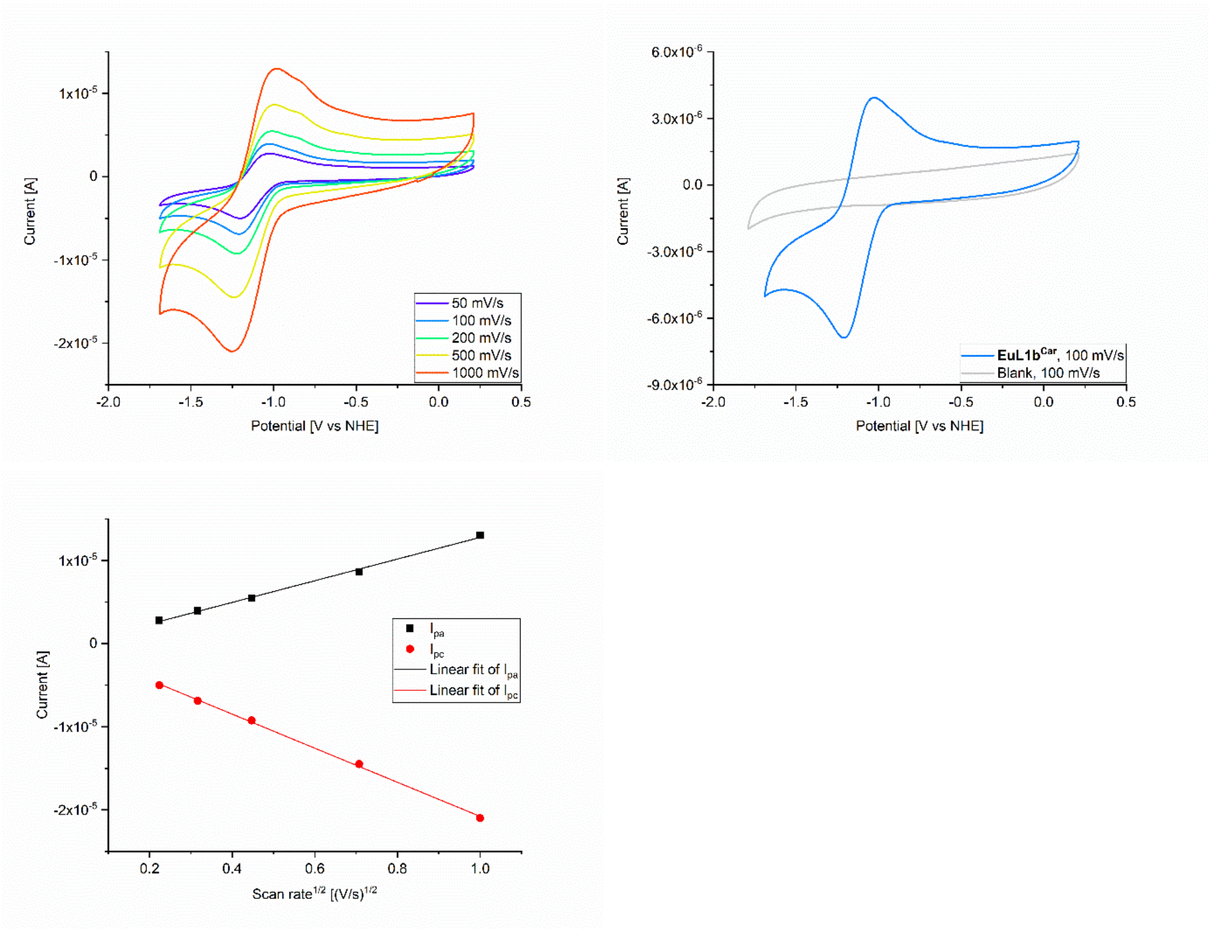

Figure S37. Cyclic voltammograms at various scan rates for **EuL1b^{Car}** in DMF and plot of I_{pa} and I_{pc} vs. square root of scan rate.

Table S14. Values for linear fit of I_{pa} and I_{pc} *vs*. square root of scan rate for **EuL1b^{Car}** in DMF.

**Equation: y = a*x + b**	**I_{pa}**	**I_{pc}**
Slope (a)	$1.30\cdot10^{-5} \pm 3.89\cdot10^{-7}$	$-2.05\cdot10^{-5} \pm 4.01\cdot10^{-7}$
Intercept (b)	$-2.56\cdot10^{-7} \pm 2.37\cdot10^{-7}$	$-2.84\cdot10^{-6} \pm 2.44\cdot10^{-7}$
R-Square (COD)	0.99733	0.99885

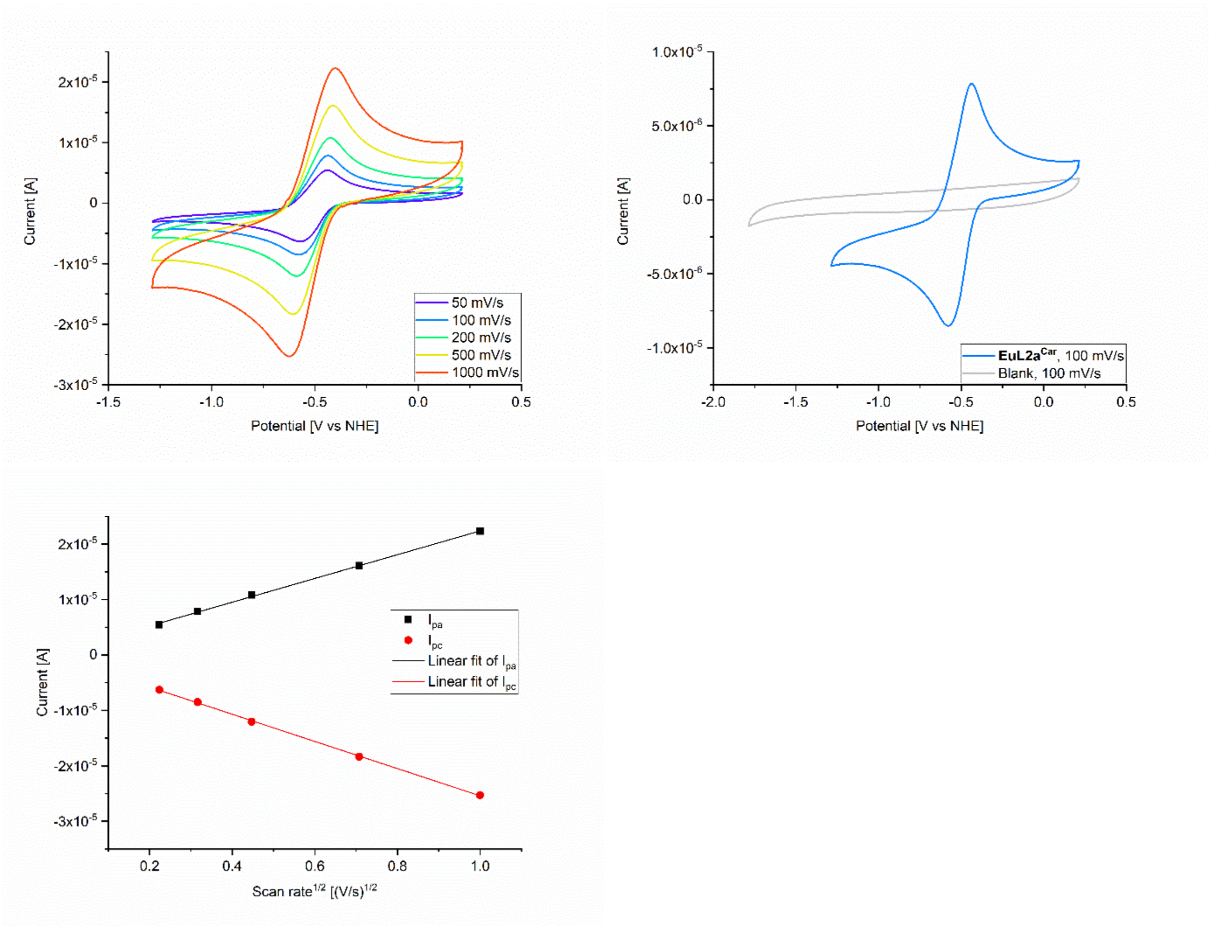

Figure S38. Cyclic voltammograms at various scan rates for **EuL2a^{Car}** in DMF and plot of I_{pa} and I_{pc} vs. square root of scan rate.

Table S15. Values for linear fit of I_{pa} and I_{pc} *vs*. square root of scan rate for **EuL2a^{Car}** in DMF.

**Equation: y = a*x + b**	$\mathbf{I_{pa}}$	$\mathbf{I_{pc}}$
Slope (a)	$2.15 \cdot 10^{-5} \pm 3.85 \cdot 10^{-7}$	$-2.45 \cdot 10^{-5} \pm 2.59 \cdot 10^{-7}$
Intercept (b)	$9.37 \cdot 10^{-7} \pm 2.34 \cdot 10^{-7}$	$-8.90 \cdot 10^{-7} \pm 1.58 \cdot 10^{-7}$
R-Square (COD)	0.99903	0.99967

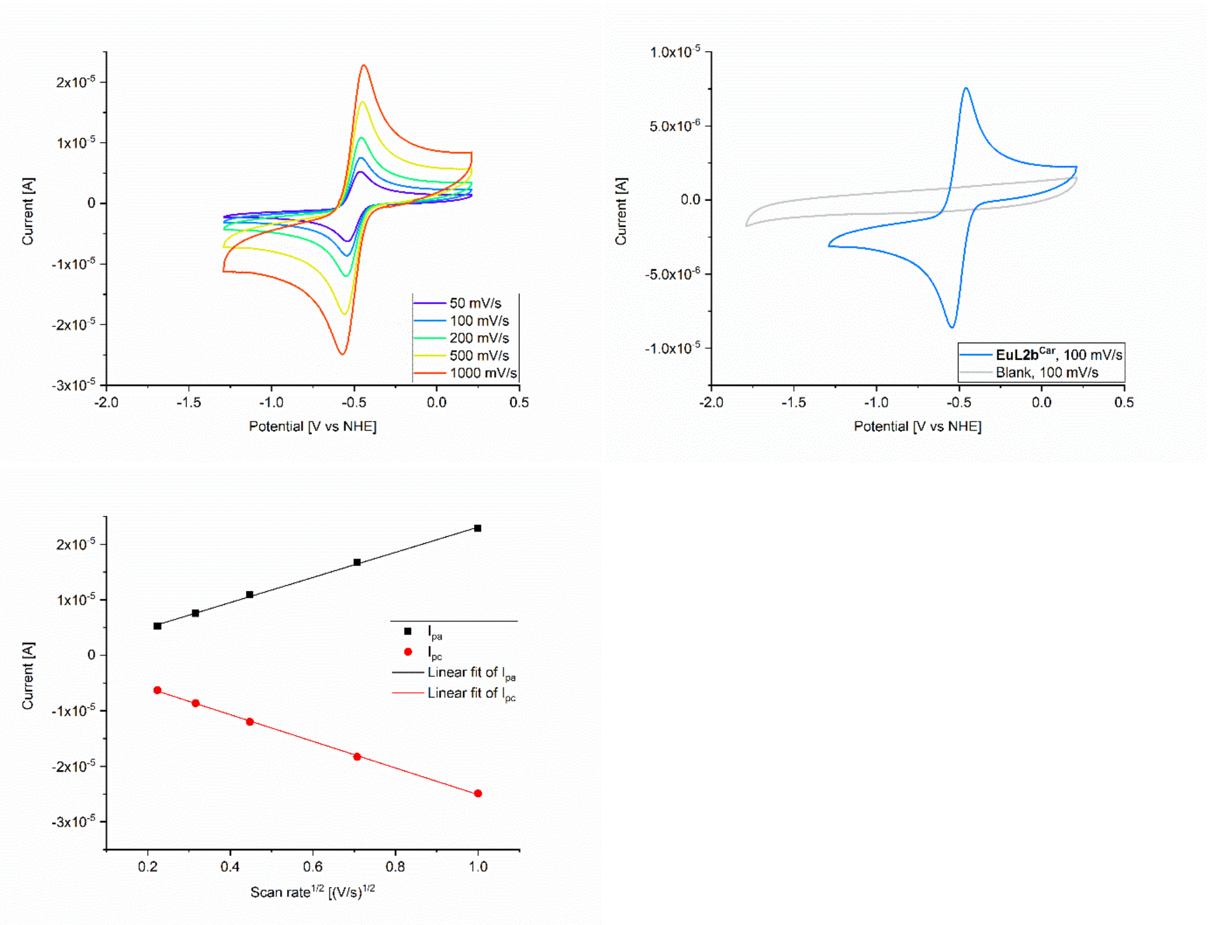

Figure S39. Cyclic voltammograms at various scan rates for **EuL2b$^{\mathbf{Car}}$** in DMF and plot of I_{pa} and I_{pc} vs. square root of scan rate.

Table S16. Values for linear fit of I_{pa} and I_{pc} *vs*. square root of scan rate for **EuL2b$^{\mathbf{Car}}$** in DMF.

**Equation: y = a*x + b**	$\mathbf{I_{pa}}$	$\mathbf{I_{pc}}$
Slope (a)	$2.26·10^{–5} ± 5.08·10^{–7}$	$–2.40·10^{–5} ± 3.64·10^{–7}$
Intercept (b)	$4.48·10^{–7} ± 3.09·10^{–7}$	$–1.09·10^{–6} ± 2.22·10^{–7}$
R-Square (COD)	0.99849	0.99931

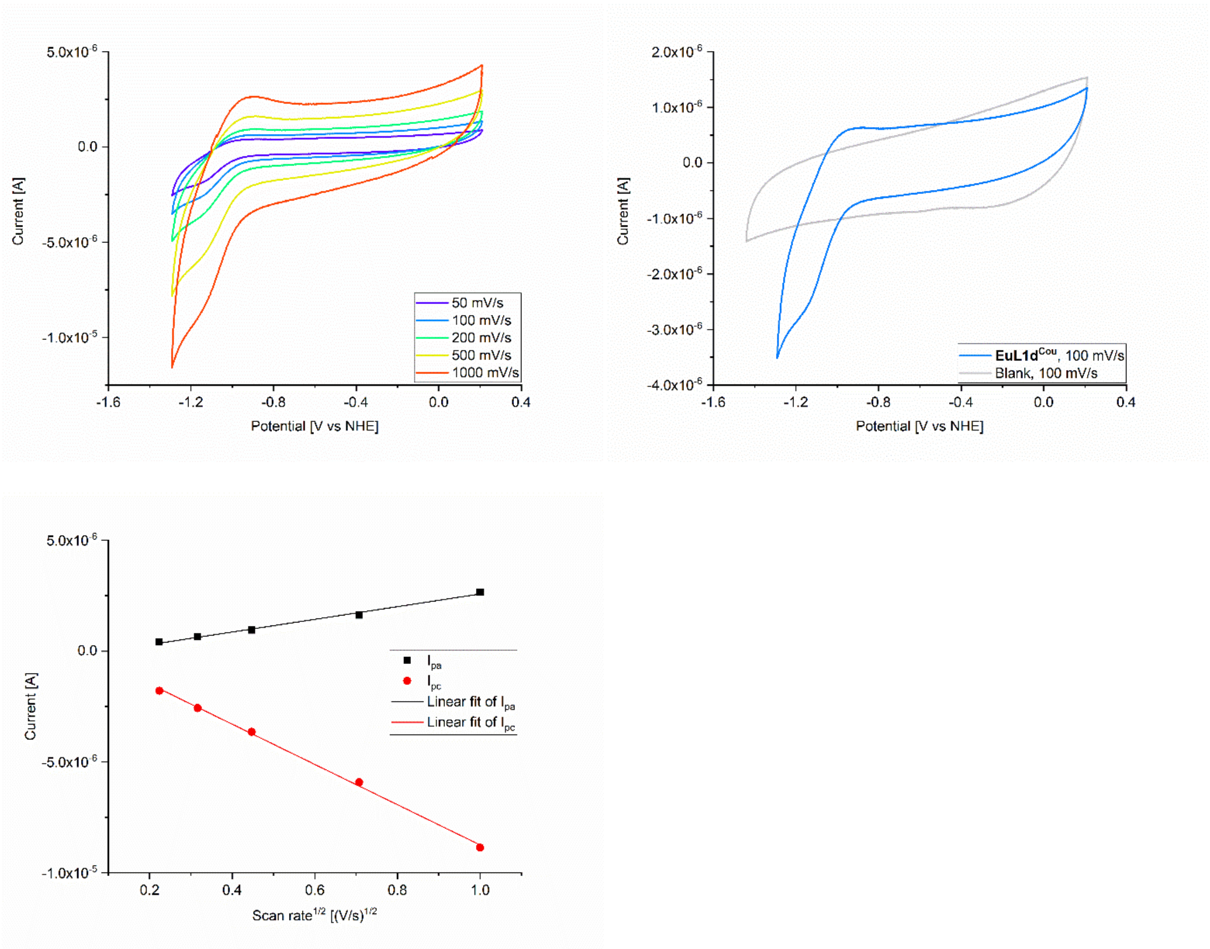

Figure S40. Cyclic voltammograms at various scan rates for **EuL1d$^{\mathbf{Cou}}$** in DMF and plot of I_{pa} and I_{pc} vs. square root of scan rate.

Table S17. Values for linear fit of I_{pa} and I_{pc} *vs.* square root of scan rate for **EuL1d$^{\mathbf{Cou}}$** in DMF.

**Equation: y = a*x + b**	$\mathbf{I_{pa}}$	$\mathbf{I_{pc}}$
Slope (a)	$2.86{\cdot}10^{-6} \pm 1.53{\cdot}10^{-7}$	$-9.08{\cdot}10^{-6} \pm 2.25{\cdot}10^{-7}$
Intercept (b)	$-2.83{\cdot}10^{-7} \pm 9.33{\cdot}10^{-8}$	$3.34{\cdot}10^{-7} \pm 1.37{\cdot}10^{-7}$
R-Square (COD)	0.99145	0.99816

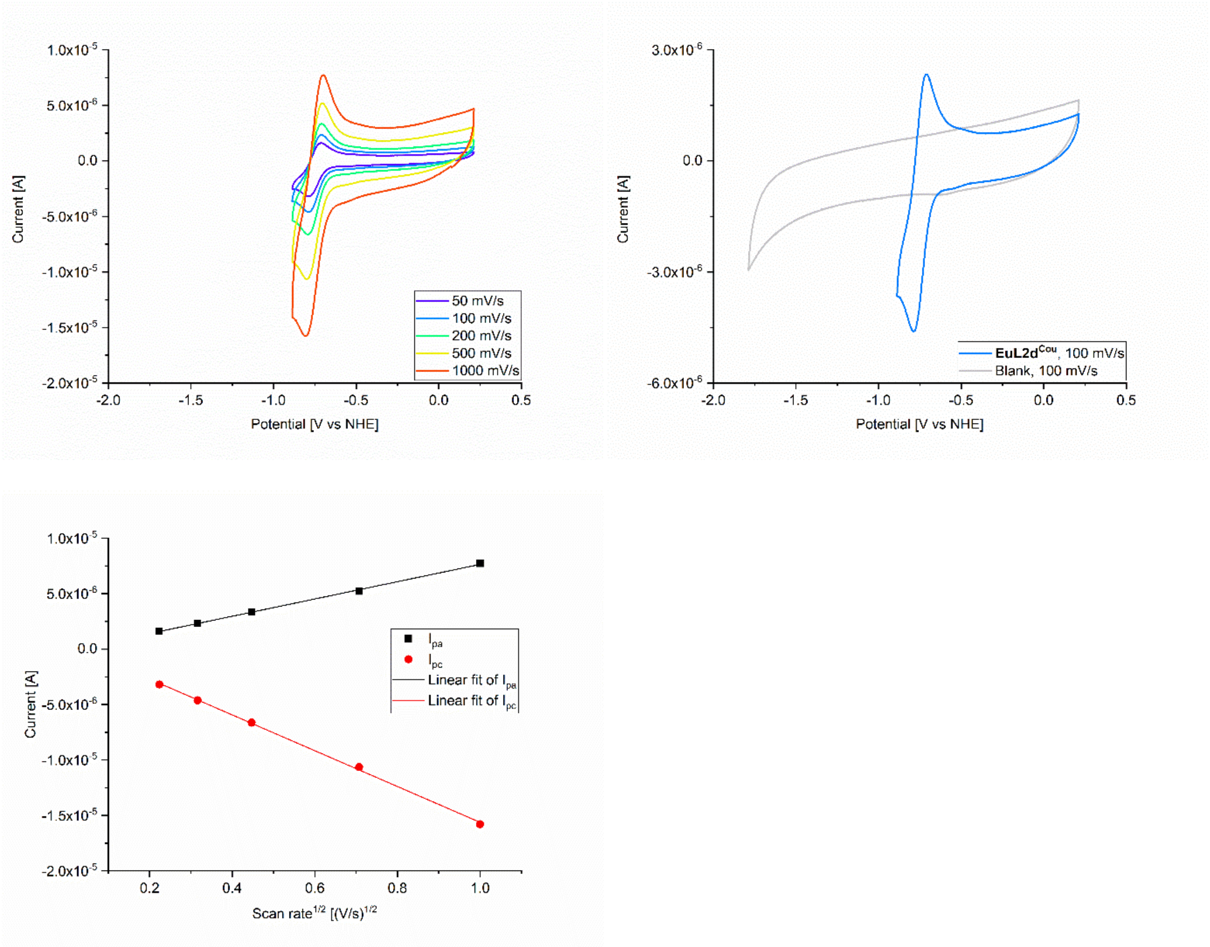

Figure S41. Cyclic voltammograms at various scan rates for **EuL2d^{Cou}** in DMF and plot of I_{pa} and I_{pc} vs. square root of scan rate.

Table S18. Values for linear fit of I_{pa} and I_{pc} *vs*. square root of scan rate for **EuL2d^{Cou}** in DMF.

**Equation: y = a*x + b**	**I_{pa}**	**I_{pc}**
Slope (a)	$7.80 \cdot 10^{-6} \pm 1.65 \cdot 10^{-7}$	$-1.61 \cdot 10^{-5} \pm 2.89 \cdot 10^{-7}$
Intercept (b)	$-1.55 \cdot 10^{-7} \pm 1.00 \cdot 10^{-7}$	$5.25 \cdot 10^{-7} \pm 1.76 \cdot 10^{-7}$
R-Square (COD)	0.99866	0.99904

Photophysical Characterization

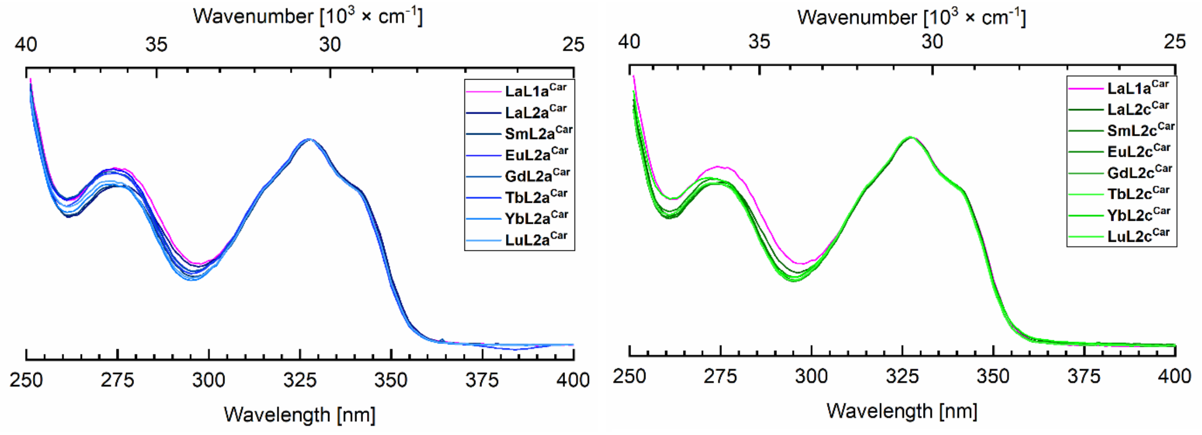

Figure S42. Superimposed UV-Vis absorption spectra of **LaL1a**$^{\mathbf{Car}}$ (magenta line) with **LnL2a**$^{\mathbf{Car}}$ (Ln = La, Sm, Eu, Gd, Tb, Yb, Lu) (from dark to light blue lines, left) and **LnL2c**$^{\mathbf{Car}}$ (Ln = La, Sm, Eu, Gd, Tb, Yb, Lu) (from dark to light green lines, right) complexes normalized at 328 nm. [**LnL**] = 10 μM and was measured in aqueous 10 mM PIPES buffer at pH 6.5 at 293 K.

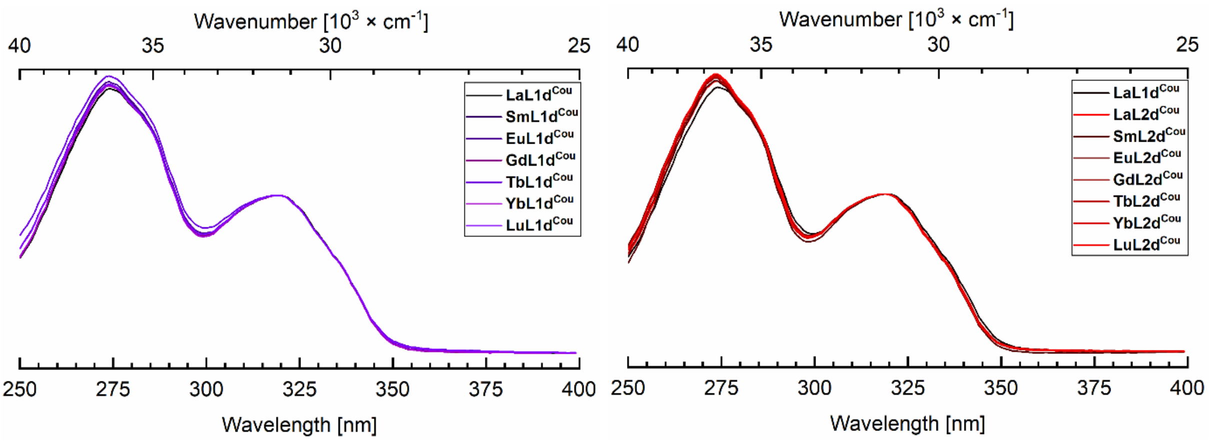

Figure S43. Superimposed UV-Vis absorption spectra of **LnL1d**$^{\mathbf{Cou}}$ (Ln = La, Sm, Eu, Gd, Tb, Yb, Lu) (from black to light purple lines, left) and **LaL1d**$^{\mathbf{Cou}}$ (black line) with **LnL2d**$^{\mathbf{Cou}}$ (Ln = La, Sm, Eu, Gd, Tb, Yb, Lu) (from dark to light red lines, right) complexes normalized at 319 nm. [**LnL**] = 10 μM and was measured in aqueous 10 mM PIPES buffer at pH 6.5 at 293 K.

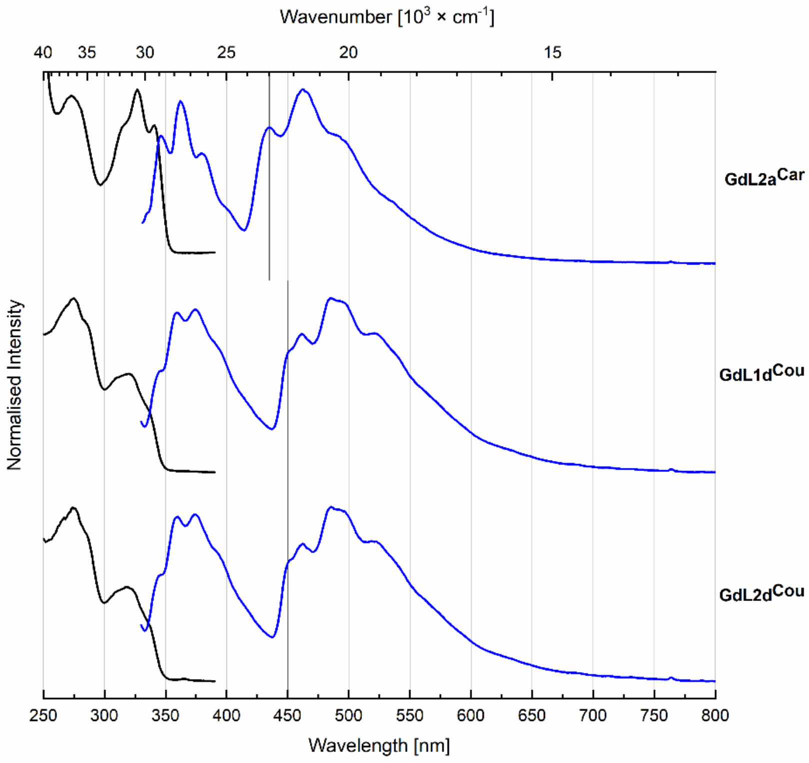

Figure S44. Excitation spectra of the ligand centered phosphorescence emissions of **GdL** complexes (black lines, λ_{em} = 435 nm (**GdL2a^{Car}**), 462 nm (**GdL1-2d^{Cou}**)), and their steady-state emission spectra (blue lines, λ_{ex} = 327 nm (**GdL2a^{Car}**), 315 nm (**GdL1-2d^{Cou}**)) at 77 K. [**GdL**] = 10 µM with 10% glycerol in 10 mM PIPES buffer aqueous solutions at pH 6.5. The dark grey lines are at the maxima of the first visible vibronic component of the phosphorescence spectra (λ_{em} = 435 nm (**GdL2a^{Car}**), 450 nm (**GdL1-2d^{Cou}**)).

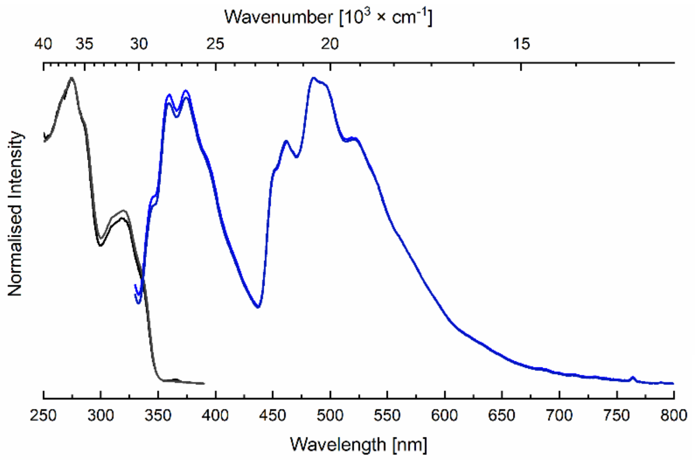

Figure S45. Superimposed excitation spectra of the ligand centered phosphorescence emissions of Gd complexes (black lines, λ_{em}(**GdL1-2d^{Cou}**) = 462 nm), and their steady-state emission spectra (blue lines, λ_{ex}(**GdL1-2d^{Cou}**) = 315 nm) at 77 K. [**GdL^{Cou}**] = 10 µM with 10% glycerol in 10 mM PIPES buffer aqueous solutions at pH 6.5.

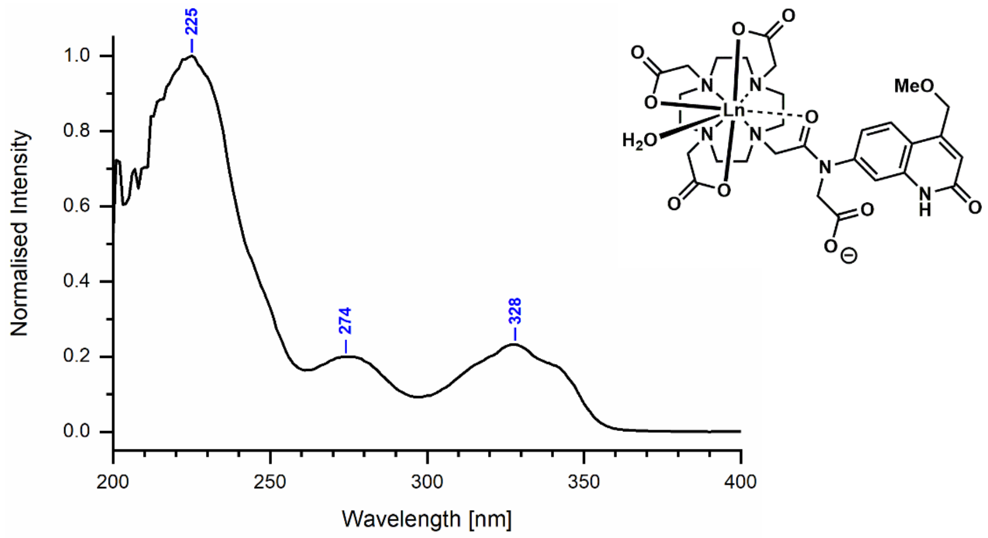

Figure S46. Full normalized UV-Vis absorption spectrum of **LaL1a^{Car}** (10 µM in 10 mM aqueous PIPES buffer at pH 6.5) at 293 K. Blue numbers are the local maxima.

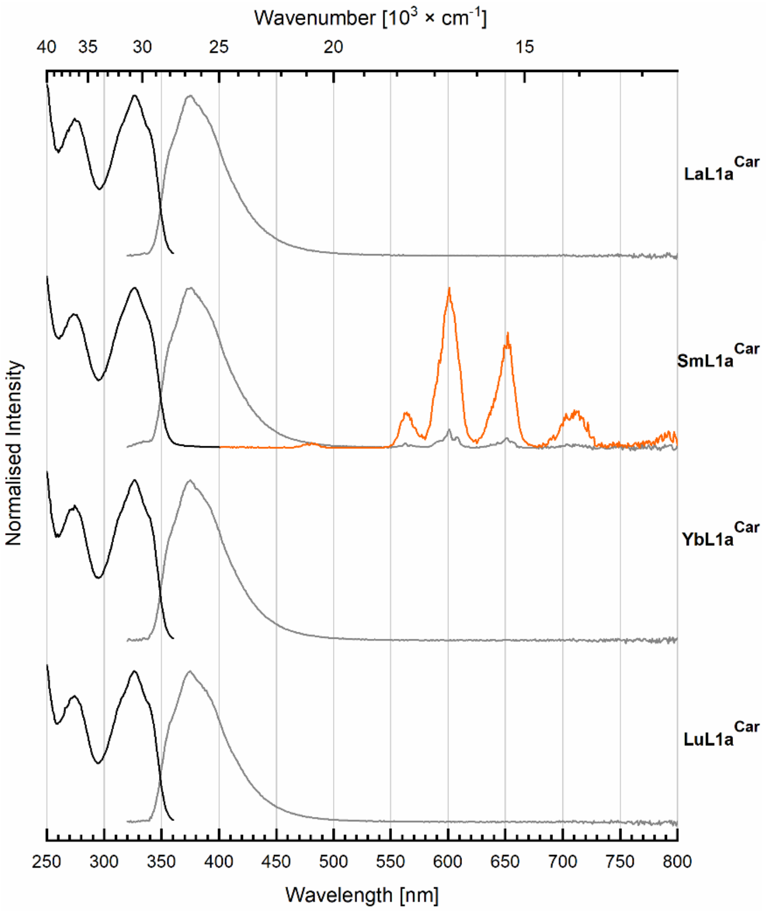

Figure S47. Excitation of the ligand-centred emissions (black, λ_{em} = 375 nm for La, Yb and Lu) and of the Ln(III) luminescence (λ_{em} = 601 nm for Sm), steady-state (gray) and time-resolved emission spectra (orange, Sm) of **LnL1a^{Car}** complexes at 293 K. [**LnL1a^{Car}**] = 10 µM in aqueous (or D_2O for time-resolved emission spectrum of **SmL1a^{Car}**) 10 mM PIPES buffer solutions at pH (pD) 6.5 with λ_{ex} = 327 nm.

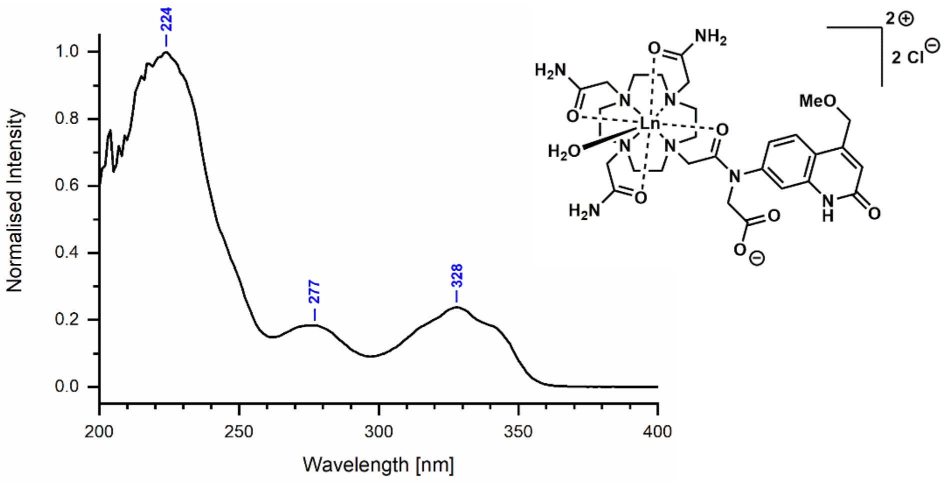

Figure S48. Full normalized UV-Vis absorption spectrum of **LaL2a^{Car}** (10 µM in 10 mM aqueous PIPES buffer at pH 6.5) at 293 K. Blue numbers are the local maxima.

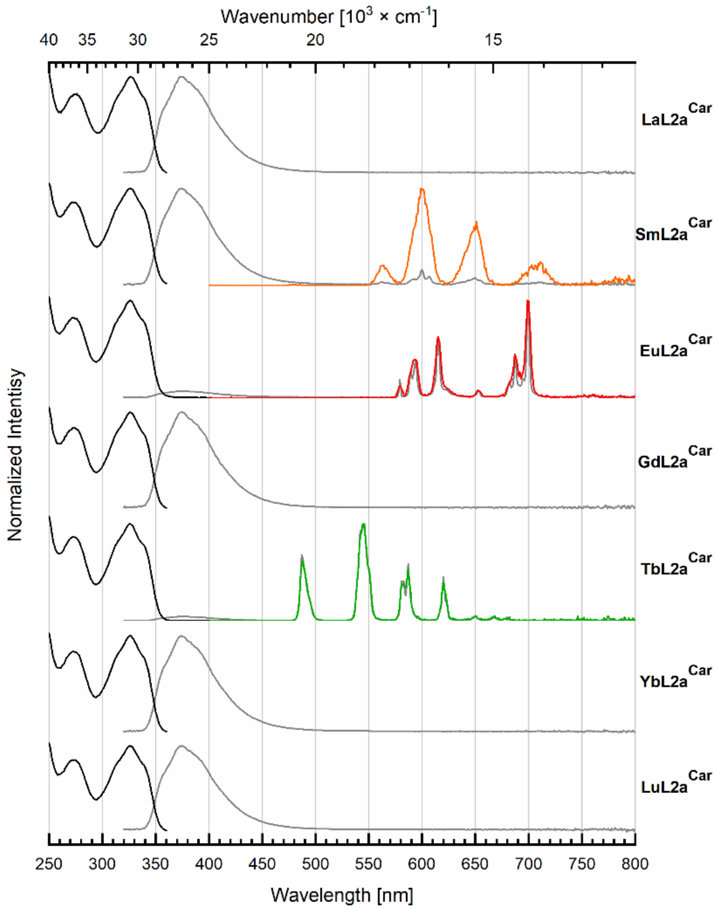

Figure S49. Excitation of the ligand-centred emissions (black, λ_{em} = 375 nm for La, Gd, Yb and Lu) and of the Ln(III) luminescence (λ_{em} = 600 nm for Sm, 615 nm for Eu, 545 nm for Tb), steady-state (gray) and time-resolved emission spectra (orange, Sm; red, Eu; green, Tb) of **LnL2a^{Car}** complexes at 293 K. [**LnL2a^{Car}**] = 10 µM in aqueous (or D_2O for time-resolved emission spectrum of **SmL2a^{Car}**) 10 mM PIPES buffer solutions at pH (pD) 6.5 with λ_{ex} = 327 nm.

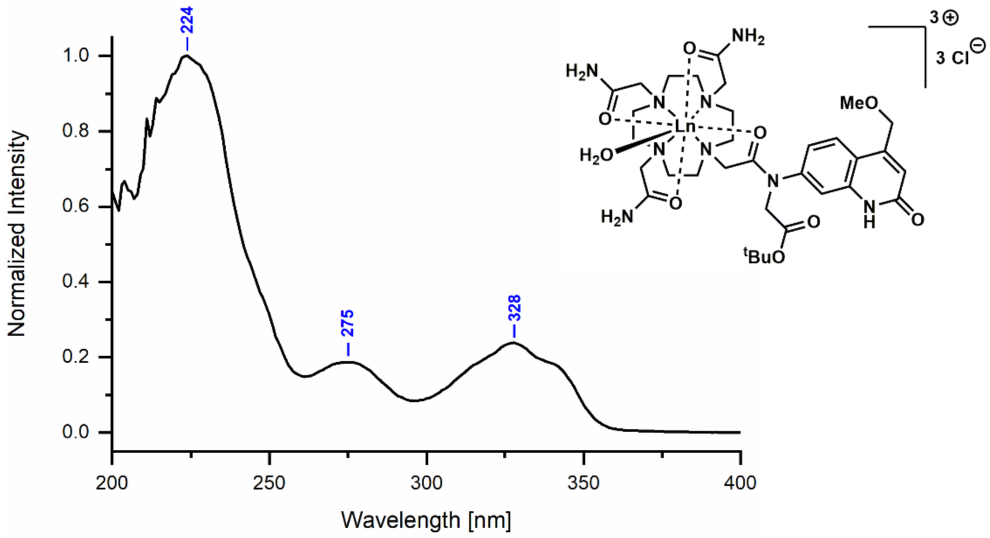

Figure S50. Full normalized UV-Vis absorption spectrum of **LaL2c^{Car}** (10 µM in 10 mM aqueous PIPES buffer at pH 6.5) at 293 K. Blue numbers are the local maxima.

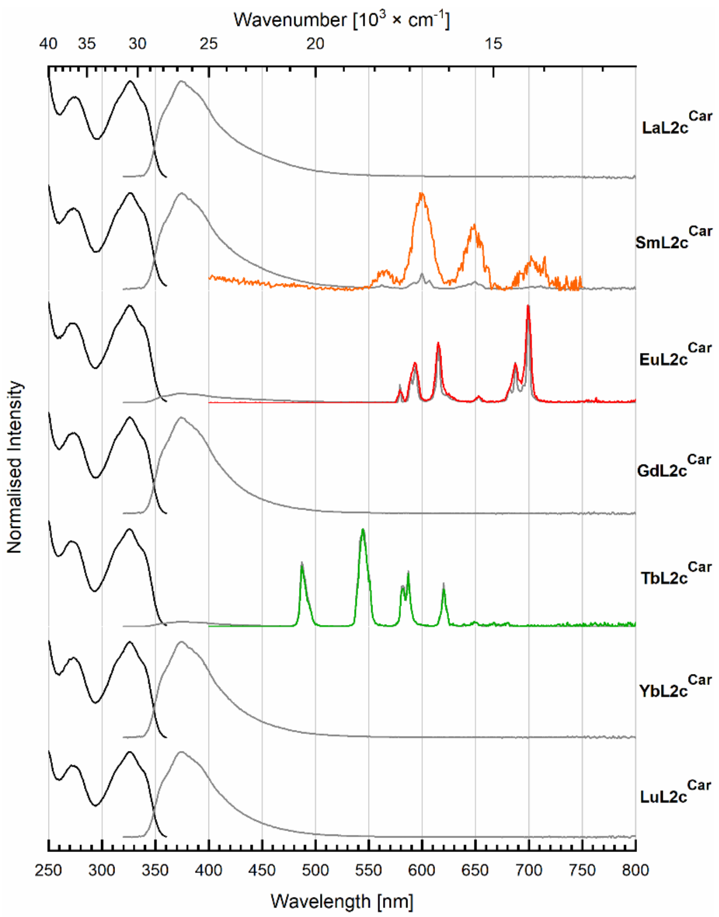

Figure S51. Excitation (black) of the ligand-centred emissions (λ_{em} = 375 nm for La, Gd, Yb and Lu) and of the Ln(III) luminescence (λ_{em} = 600 nm for Sm, 615 nm for Eu, 545 nm for Tb), steady-state (gray) and time-resolved emission spectra (orange, Sm; red, Eu; green, Tb) of **LnL2c^{Car}** complexes at 293 K. [**LnL2c^{Car}**] = 10 µM in aqueous 10 mM PIPES buffer solutions at pH 6.5 with λ_{ex} = 327 nm.

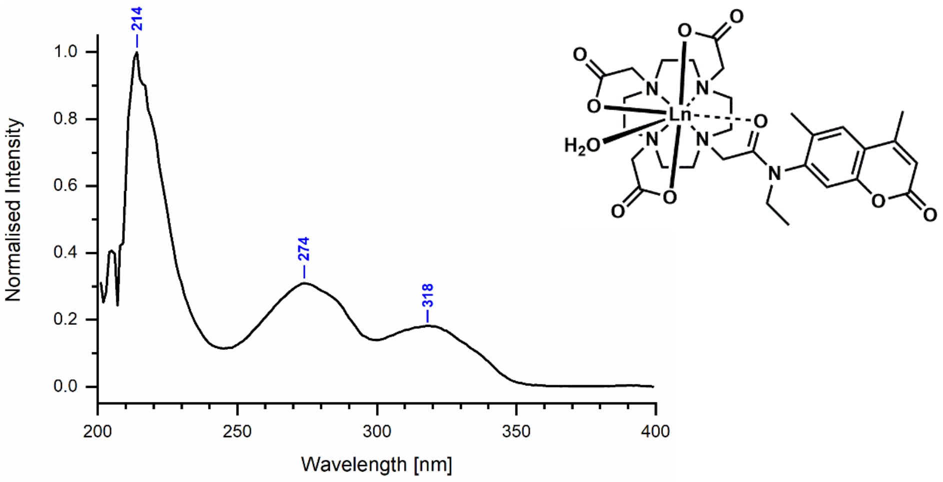

Figure S52. Full normalized UV-Vis absorption spectrum of **LaL1d**$^{\mathbf{Cou}}$ (10 µM in 10 mM aqueous PIPES buffer at pH 6.5) at 293 K. Blue numbers are the local maxima.

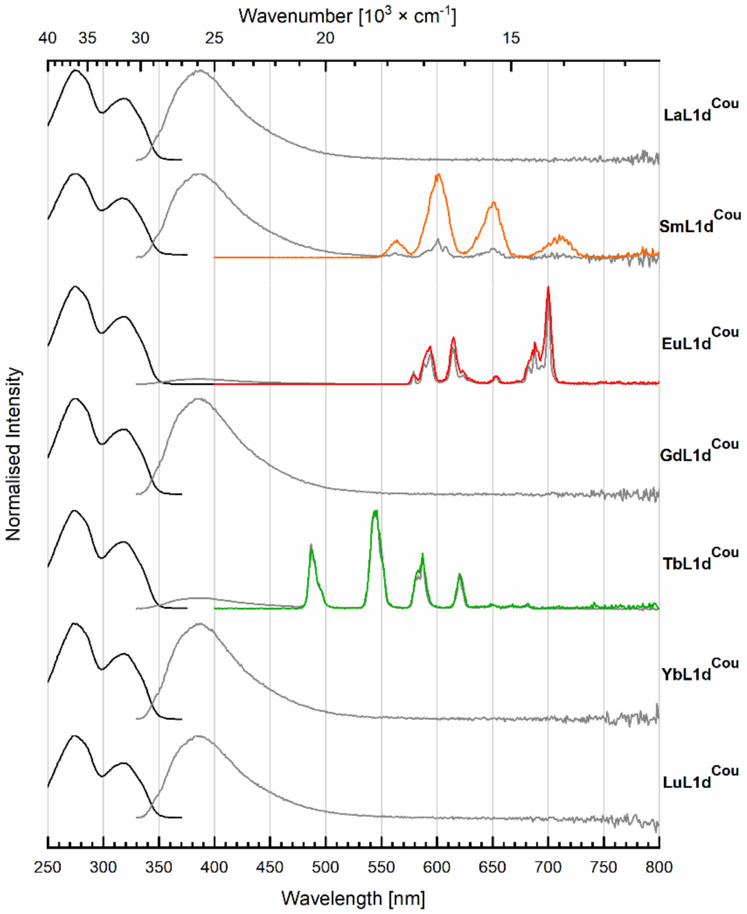

Figure S53. Excitation (black) of the ligand-centred emissions (λ_{em} = 385 nm for La, Gd, Yb and Lu) and of the Ln(III) luminescence (λ_{em} = 601 nm for Sm, 614 nm for Eu, 545 nm for Tb), steady-state (gray) and time-resolved emission spectra (orange, Sm; red, Eu; green, Tb) of **LnL1d**$^{\mathbf{Cou}}$ complexes at 293 K. [**LnL1d**$^{\mathbf{Cou}}$] = 10 µM in aqueous (or D_2O for time-resolved emission spectrum of **SmL1d**$^{\mathbf{Cou}}$) 10 mM PIPES buffer solutions at pH (pD) 6.5 with λ_{ex} = 315 nm.

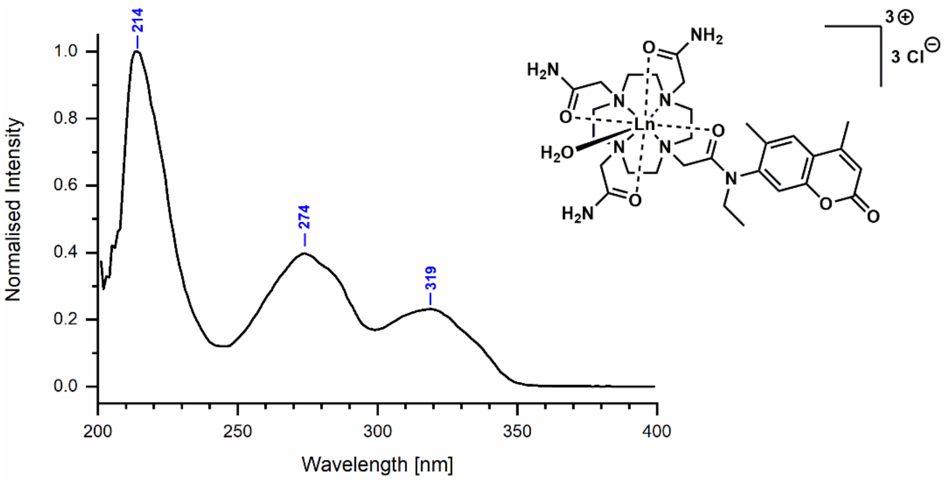

Figure S54. Full normalized UV-Vis absorption spectrum of **LaL2d^{Cou}** (10 µM in 10 mM aqueous PIPES buffer at pH 6.5) at 293 K. Blue numbers are the local maxima.

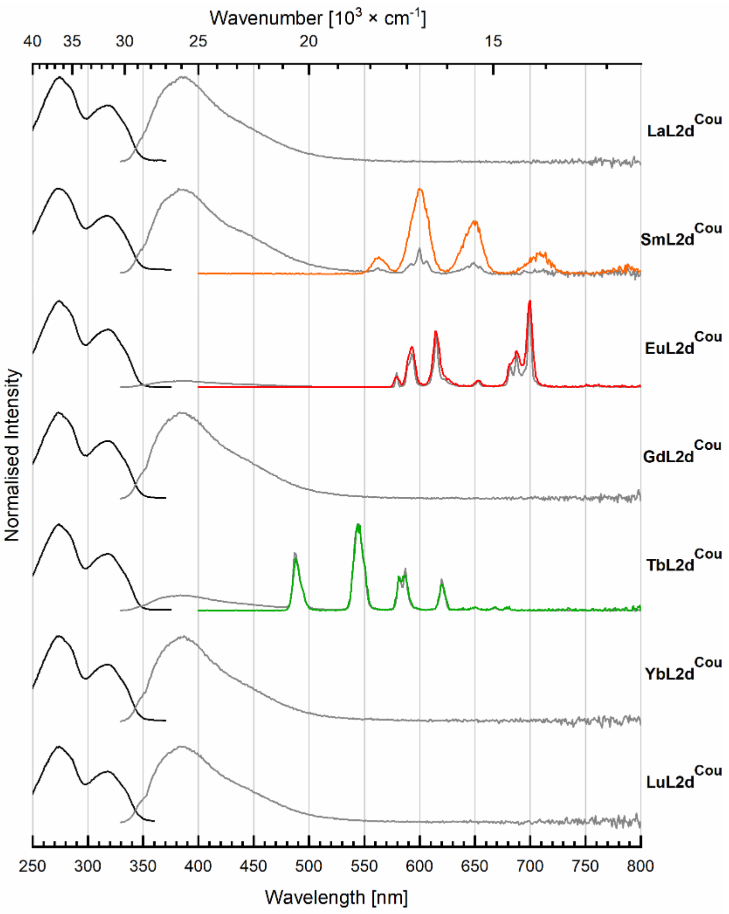

Figure S55. Excitation (black) of the ligand-centred emissions (λ_{em} = 385 nm for La, Gd, Yb and Lu) and of the Ln(III) luminescence (λ_{em} = 600 nm for Sm, 615 nm for Eu, 545 nm for Tb), steady-state (gray) and time-resolved emission spectra (orange, Sm; red, Eu; green, Tb) of **LnL2d^{Cou}** complexes at 293 K. [**LnL2d^{Cou}**] = 10 µM in aqueous (or D_2O for time-resolved emission spectrum of **SmL1d^{Cou}**) 10 mM PIPES buffer solutions at pH (pD) 6.5 with λ_{ex} = 315 nm.

Table S19. Antenna and Eu(III)-based luminescence quantum yields from several independent experiments of **EuL** complexes. Measurements were performed with [**EuL**] = 10 µM in 10 mM PIPES-buffered H_2O at pH 6.5.

Complex	Φ_L [a]	$\Phi_{L,av}$ [b]	Standard Deviation	Φ_{Ln} [a]	$\Phi_{Ln,av}$ [b]	Standard Deviation
**EuL2a^{Car}**	0.75<br>0.77<br>0.78	0.77	0.015	2.71<br>2.75<br>2.75	2.74	0.023
**EuL2c^{Car}**	1.09<br>1.26	1.18	0.120	2.51<br>2.49	2.5	0.014
**EuL1d^{Cou}**	0.61<br>0.59<br>0.65	0.62	0.031	2.12<br>2.08<br>2.32	2.17	0.129
**EuL2d^{Cou}**	0.59<br>0.56<br>0.58	0.58	0.015	1.51<br>1.39<br>1.51	1.47	0.069
**EuL1a^{Car}-F**	2.52<br>2.56	2.54	0.028	9.40<br>9.57	9.49	0.120
**EuL2a^{Car}-F**	2.06<br>2.12	2.09	0.042	12.2<br>12.3	12.25	0.071
**EuL1d^{Cou}-F**	0.63<br>0.65	0.64	0.014	2.30<br>2.37	2.34	0.050
**EuL2d^{Cou}-F**	0.64<br>0.655	0.65	0.011	3.27<br>3.40	3.34	0.092

[a] In %, relative to QS (Φ = 0.59) in H_2SO_4 (0.05 M) [4]. [b] In %, average quantum yield from two or three independent measurements.

Table S20. Antenna fluorescence quantum yields from several independent experiments of **GdL** complexes. Measurements were performed with [**GdL**] = 10 µM in 10 mM PIPES-buffered H_2O at pH 6.5.

Complex	Φ_L [a]	$\Phi_{L,av}$ [b]	Standard Deviation
**GdL2a^{Car}**	4.34<br>4.33	4.34	0.01
**GdL1d^{Cou}**	0.68<br>0.64	0.66	0.03
**GdL2d^{Cou}**	0.65<br>0.61	0.63	0.03

[a] In %, relative to QS (Φ = 0.59) in H_2SO_4 (0.05 M) [4]. [b] In %, average quantum yield from two independent measurements.

Table S21. Antenna and Sm(III)-based luminescence quantum yields from several independent experiments of **SmL** complexes. Measurements were performed with [**SmL**] = 10 µM in 10 mM PIPES-buffered H_2O at pH 6.5.

Complex	Φ_L [a]	$\Phi_{L,av}$ [b]	Standard Deviation	Φ_{Ln} [a]	$\Phi_{Ln,av}$ [b]	Standard Deviation
**SmL1a^{Car}**	3.60<br>3.66	3.63	0.042	0.198<br>0.176	0.187	0.016
**SmL2a^{Car}**	3.46<br>3.33	3.40	0.092	0.232<br>0.183	0.208	0.035
**SmL1d^{Cou}**	0.63<br>0.64	0.64	0.007	0.055<br>0.062	0.059	0.005
**SmL2d^{Cou}**	0.645<br>0.64	0.64	0.004	0.068<br>0.065	0.067	0.002

[a] In %, relative to QS (Φ = 0.59) in H_2SO_4 (0.05 M) [4]. [b] In %, average quantum yield from two or three independent measurements.

Table S22. Antenna and Sm(III)-based luminescence quantum yields of **SmL** in PIPES-buffered D_2O and changes relative to solutions in H_2O. Measurements were performed with [**SmL**] = 10 µM in 10 mM PIPES-buffered H_2O at pD 6.5.

Complex	Φ_L [a]	Φ_{Ln} [a]	$\frac{\Phi_{Ln,D2O}}{\Phi_{Ln,H2O}}$ [b]
**SmL1a^{Car}**	3.6	0.66	3.5
**SmL2a^{Car}**	3.3	0.60	2.9
**SmL1d^{Coum}**	0.63	0.18	4.7
**SmL2d^{Coum}**	0.64	0.17	3.6

[a] In %, relative to quinine sulfate (Φ = 0.59) in H_2SO_4 (0.05 M) [4]. [b] Fold increase relative to the solution in H_2O.

Table S23. Antenna and Ln(III)-based luminescence quantum yields, Ln(III) lifetimes and hydration states of **LnL2c^{Car}**.

Complex	Φ_L [%] [a]	Φ_{Ln} [%] [a]	τ_{H2O} [ms]	τ_{D2O} [ms]	q [b]
**LaL2c^{Car}**	4.7	-	-	-	-
**SmL2c^{Car}**	3.6	0.19	0.011	0.033	-
**EuL2c^{Car}**	1.2	2.5	0.52	1.98	0.9
**GdL2c^{Car}**	4.7	-	-	-	-
**TbL2c^{Car}**	4.0	30.2	1.46	2.65	1.2
**YbL2c^{Car}**	4.5	-	-	-	-
**LuL2c^{Car}**	4.7	-	-	-	-

Measurements were performed with [**LnL2c^{Car}**] = 10µM in 10 mM aqueous PIPES buffer solutions at pH (pD) 6.5. [a] Relative to quinine sulfate (Φ = 0.59) in H_2SO_4 (0.05 M) [4]. [b] Calculated using the equation $q = 5(1/\tau_{H2O} - 1/\tau_{D2O} - 0.06)$ for Tb, and $q = 1.2(1/\tau_{H2O} - 1/\tau_{D2O} - 0.25 - n \times 0.075)$, where n is the number of nearby N-H oscillators, for Eu [8].

Table S24. Antenna and Eu(III)-based luminescence quantum yields from several independent experiments of **EuL2c^{Car}** complexes. Measurements were performed with [**EuL2c^{Car}**] = 10 μM in 10 mM PIPES-buffered D_2O at pD 6.5.

Complex	Φ_L [a]	$\Phi_{L,av}$ [b]	Standard Deviation	Φ_{Ln} [a]	$\Phi_{Ln,av}$ [b]	Standard Deviation
**EuL2c^{Car}**	1.175<br>1.20	1.19	0.018	11.8<br>11.2	11.5	0.424
**EuL2c^{Car}–F**	2.54<br>2.65	2.6	0.078	30.2<br>32.3	31.3	1.485

[a] In %, relative to QS (Φ = 0.59) in H_2SO_4 (0.05 M) [4]. [b] In %, average quantum yield from two or three independent measurements.

Table S25. Decay rates (*k*) and the amount of excitation energy lost to X-H quenching (*Loss*) of **EuL** complexes. [a]

Complex	k_{H2O} [b]	k_{D2O} [b]	*Loss* (%) [c]
**EuL1a^{Car}**	1.52	0.461	70
**EuL2a^{Car}**	1.96	0.488	75
**EuL1d^{Cou}**	1.61	0.510	68
**EuL2d^{Cou}**	1.85	0.498	73

[a] [**EuL**] = 10 μM in 10 mM aqueous or D_2O PIPES buffer solutions at pH (pD) 6.5. [b] In ms^{-1}, calculated as $k = 1/\tau_{obs}$. [c] In ms, calculated as $(k_{H2O}–k_{D2O})/k_{H2O}$.

Table S26. Decay rates (*k*) and asymmetry values (*r*) of **EuL** complexes in H_2O and D_2O. [a]

Complex	$k_{rad,Ln}$ [b]	$k_{nr,Ln}$ [b]	r_{H2O} [c]	$k_{rad,Ln}(D_2O)$ [b]	$k_{nr,Ln}(D_2O)$ [b]	r_{D2O} [c]
**EuL1a^{Car}**	0.187	1.33	1.13	0.186	0.275	1.12
**EuL2a^{Car}**	0.196	1.77	1.39	0.194	0.294	1.41
**EuL1d^{Cou}**	0.190	1.42	1.18	0.188	0.322	1.19
**EuL2d^{Cou}**	0.199	1.65	1.42	0.196	0.302	1.41

[a] [**EuL**] = 10 μM in 10 mM aqueous or D_2O PIPES buffer solutions at pH (pD) 6.5. [b] In ms^{-1}. $k_{rad,Ln} = 1/\tau_{rad,Ln}$; $k_{nr,Ln} = 1/\tau_{obs} - k_{rad,Ln}$. [c] The ratio of $I_{J=2}/I_{J=1}$, where $I_{J=2}$ is integral of the $^5D_0 \rightarrow {}^7F_2$ emission band (604–640 nm) and $I_{J=1}$ is integral of the $^5D_0 \rightarrow {}^7F_1$ emission band (582–603 nm).

Table S27. Photophysical properties of **EuL2c^{Car}** in PIPES-buffered H_2O and D_2O. [a]

Complex	$\tau_{rad,Ln}$ [b, c]	$k_{rad,Ln}$ [d]	τ_{obs} [b]	$k_{nr,Ln}$ [d]	Φ_{Ln}^{Ln} [c, e]	η_{sens} [c, e]	r [f]
**EuL2c^{Car}**	5.22	0.192	0.52	1.73	9.9	25.4	1.37
**EuL2c^{Car}** (D_2O)	5.06	0.198	1.98	0.307	39.1	28.7	1.50
**EuL2c^{Car}–F** (D_2O)	4.87	0.205	2.38	0.215	48.9	61.6	2.21

[a] [**EuL2c^{Car}**] = 10 µM and was measured in 10 mM aqueous or D_2O PIPES buffer solutions at pH (pD) 6.5. [b] In ms. [c] Calculated using Eq. 1 and 2. [d] In ms^{-1}. $k_{rad,Ln} = 1/\tau_{rad,Ln}$; $k_{nr,Ln} = 1/\tau_{obs} - k_{rad,Ln}$. [e] In %. [f] The ratio of $I_{J=2}/I_{J=1}$, where $I_{J=2}$ is integral of the $^5D_0 \rightarrow {}^7F_2$ emission band (604–640 nm) and $I_{J=1}$ is integral of the $^5D_0 \rightarrow {}^7F_1$ emission band (582–603 nm).

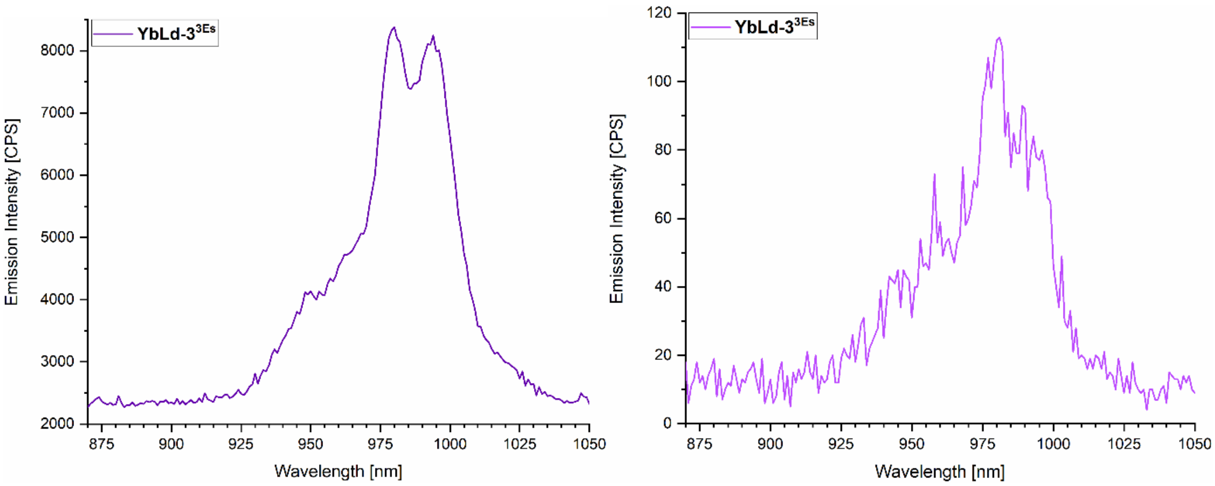

Figure S56. NIR emission spectra of fresh samples of **YbLd-3^{3Es}** from [2] (left) and measured after 4 years on the same instrument (right) showing relative emission intensities under similar sample absorbance. [**YbLd-3^{3Es}**] = 10 µM in 10 mM aqueous PIPES buffer, λ_{ex} = 344 nm, front slits: 14.7 nm, exit slits: 14.7 nm.

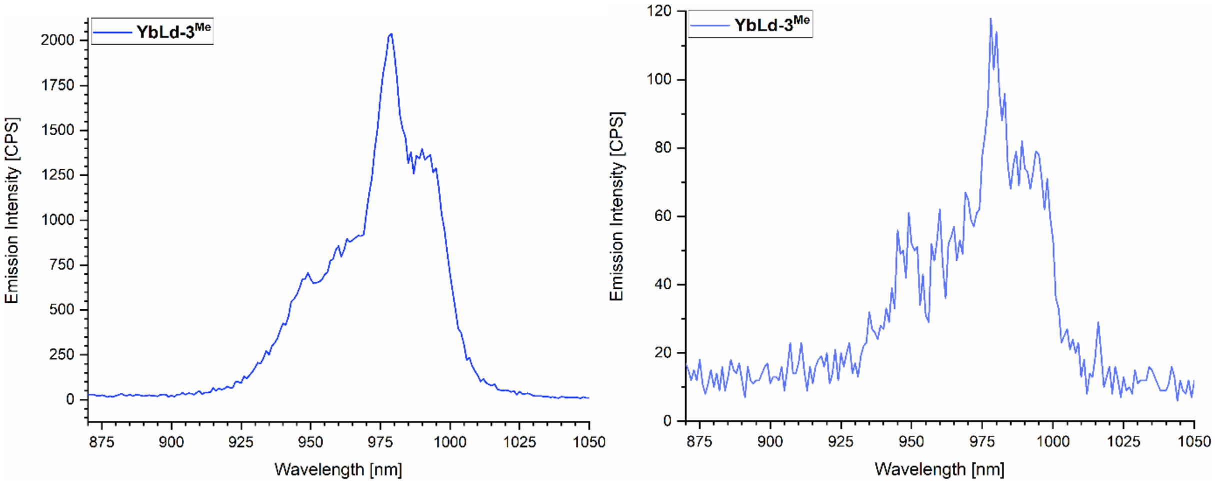

Figure S57. NIR emission spectra of fresh sample of **YbLd-3^{Me}** from [2] (left) and measured after 4 years on the same instrument (right) showing relative emission intensities under similar sample absorbance. [**YbLd-3^{Me}**] = 10 µM in 10 mM aqueous PIPES buffer, λ_{ex} = 342 nm, front slits: 14.7 nm, exit slits: 14.7 nm.

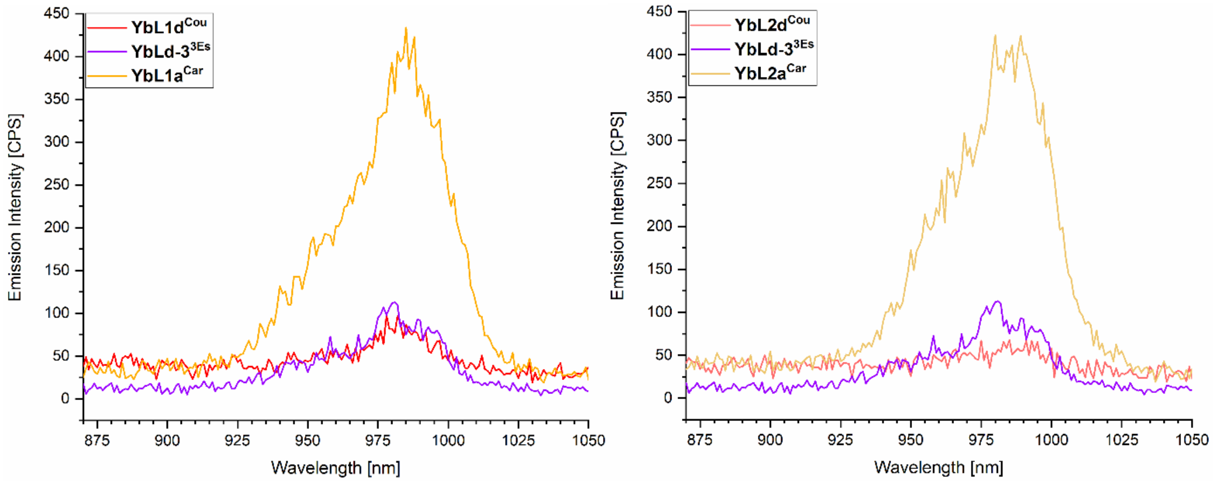

Figure S58. NIR emission spectra of **YbLd-3^{3Es}**, **YbL1** (left) and **YbL2** (right) complexes showing relative emission intensities under similar sample absorbance. [**YbL**] = 10 µM in 10 mM aqueous PIPES buffer, λ_{ex} = 344 (**YbLd-3^{3Es}**), 328 (**YbL1-2a^{Car}**), 319–318 (**YbL1-YbL2d^{Cou}**) nm, front slits: 14.7 nm, exit slits: 14.7 nm.

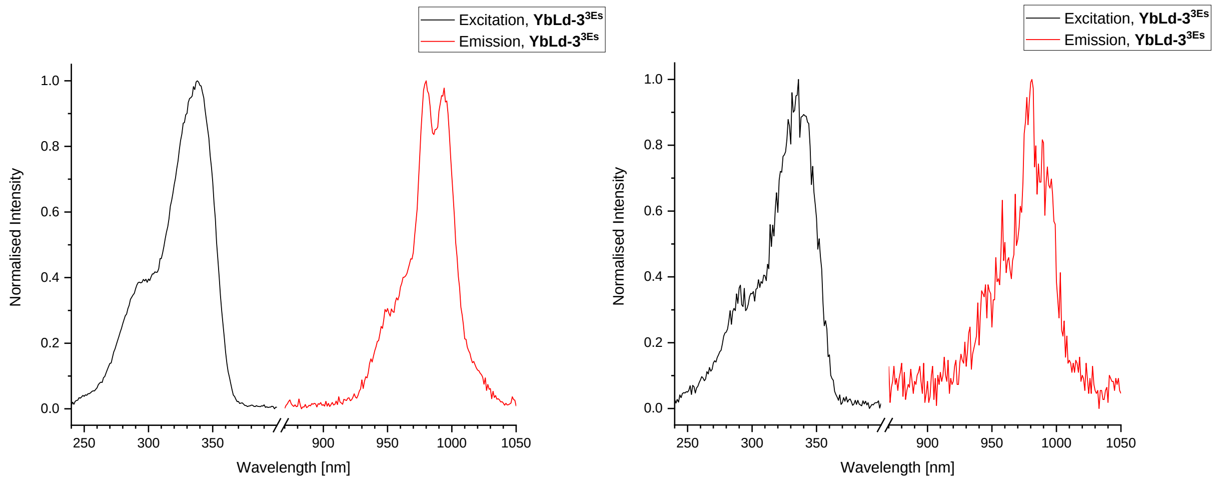

Figure S59. Normalised excitation (black) and emission (red) spectra of fresh sample of **YbLd-3^{3Es}** from [2] (left) and measured after 4 years (right). [**YbLd-3^{3Es}**] = 10 µM in 10 mM aqueous PIPES buffer, λ_{em} = 980 nm, λ_{ex} = 344 nm, front slits: 14.7 nm, exit slits: 14.7 nm.

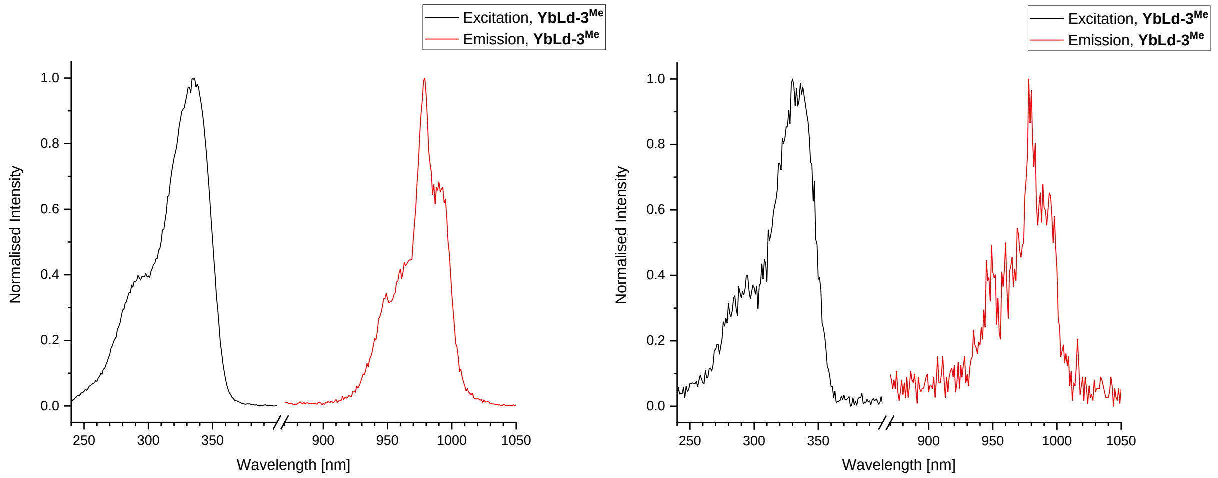

Figure S60. Normalised excitation (black) and emission (red) spectra of fresh samples of **YbLd-3^{Me}** from [2] (left) and measured after 4 years (right). [**YbLd-3^{Me}**] = 10 µM in 10 mM aqueous PIPES buffer, λ_{em} = 979 nm, λ_{ex} = 342 nm, front slits: 14.7 nm, exit slits: 14.7 nm.

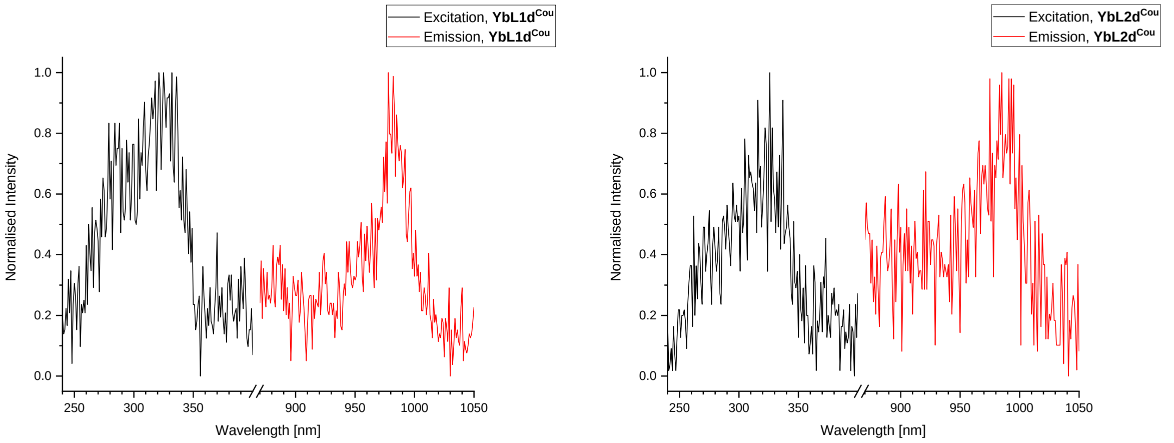

Figure S61. Normalised excitation (black) and emission (red) spectra of **YbL1d$^{\mathbf{Cou}}$** (left) and **YbL2d$^{\mathbf{Cou}}$** (right). [**YbL$^{\mathbf{Cou}}$**] = 10 µM in 10 mM aqueous PIPES buffer, λ_{em} = 978 (**YbL1d$^{\mathbf{Cou}}$**) and 985 (**YbL2d$^{\mathbf{Cou}}$**) nm, λ_{ex} = 319 (**YbL1d$^{\mathbf{Cou}}$**) and 318 (**YbL2d$^{\mathbf{Cou}}$**) nm, front slits: 14.7 nm, exit slits: 14.7 nm.

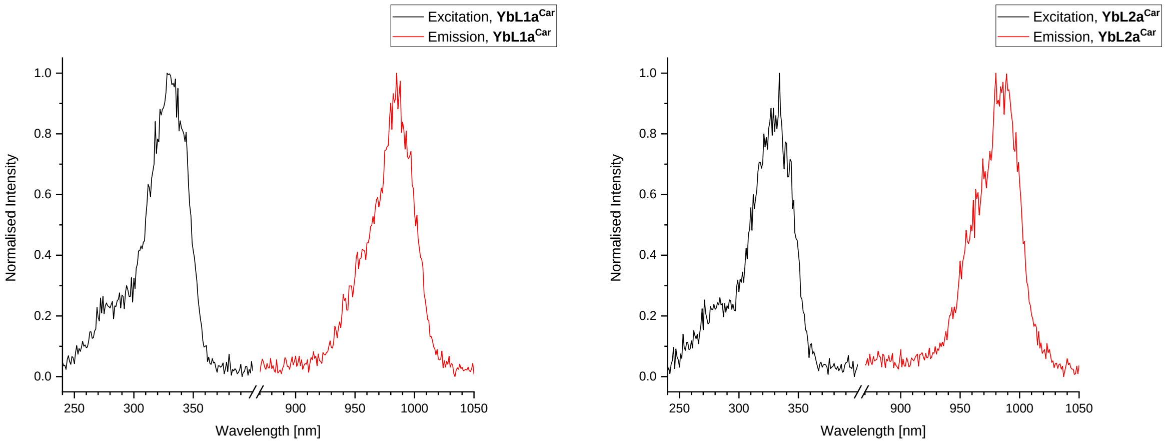

Figure S62. Normalised excitation (black) and emission (red) spectra of **YbL1a$^{\mathbf{Car}}$** (left) and **YbL2a$^{\mathbf{Car}}$** (right). [**YbL$^{\mathbf{Car}}$**] = 10 µM in 10 mM aqueous PIPES buffer, λ_{em} = 985 (**YbL1a$^{\mathbf{Car}}$**) and 980 (**YbL2a$^{\mathbf{Car}}$**) nm, λ_{ex} = 328 nm, front slits: 14.7 nm, exit slits: 14.7 nm.

Table S28. Decay rates (*k*), hydration states (*q*) of **EuL-F** complexes and the amount of excitation energy lost to X-H quenching (*Loss*). [a]

Complex	$k_{\mathrm{H2O,F}}$ [b]	$k_{\mathrm{D2O,F}}$ [b]	*Loss* (%) [c]	q [d]
**EuL1a^{Car}-F**	1.47	0.437	70	0.9
**EuL2a^{Car}-F**	0.99	0.417	58	0
**EuL1d^{Cou}-F**	1.45	0.444	69	0.9
**EuL2d^{Cou}-F**	0.99	0.418	58	0

[a] Formed by the addition of excess KF (0.1 M, 10^4-fold excess) to a solution of **EuL**. [**EuL**] = 10 μM in 10 mM aqueous or D_2O PIPES buffer solutions at pH (pD) 6.5. [b] In ms^{-1}, calculated as $k = 1/\tau_{obs}$. [c] In ms, calculated as $(k_{H2O,F}–k_{D2O,F})/k_{H2O,F}$. [d] Calculated using the equation $q = 5(1/\tau_{H2O} – 1/\tau_{D2O} – 0.06)$ for Tb, and $q = 1.2(1/\tau_{H2O} – 1/\tau_{D2O} – 0.25 – n \times 0.075)$, where n is the number of nearby N-H oscillators, for Eu [8].

Table S29. Decay rates (*k*) and asymmetry values (*r*) of **EuL-F** complexes in H_2O and D_2O. [a]

Complex	$k_{\mathrm{rad,Ln}}$ [b]	$k_{\mathrm{nr,Ln}}$ [b]	r_{H2O} [c]	$k_{\mathrm{rad,Ln}}(D_2O)$ [b]	$k_{\mathrm{nr,Ln}}(D_2O)$ [b]	r_{D2O} [c]
**EuL1a^{Car}-F**	0.188	1.28	1.18	0.186	0.251	1.15
**EuL2a^{Car}-F**	0.206	0.78	2.23	0.204	0.213	2.19
**EuL1d^{Cou}-F**	0.193	1.26	1.27	0.189	0.255	1.23
**EuL2d^{Cou}-F**	0.208	0.78	2.27	0.205	0.214	2.24

[a] [**EuL**] = 10 μM in 10 mM aqueous or D_2O PIPES buffer solutions at pH (pD) 6.5. [b] In ms^{-1}. $k_{rad,Ln} = 1/\tau_{rad,Ln}$; $k_{nr,Ln} = 1/\tau_{obs} – k_{rad,Ln}$. [c] The ratio of $I_{J=2}/I_{J=1}$, where $I_{J=2}$ is integral of the $^5D_0 \rightarrow {}^7F_2$ emission band (604–640 nm) and $I_{J=1}$ is integral of the $^5D_0 \rightarrow {}^7F_1$ emission band (582–603 nm).

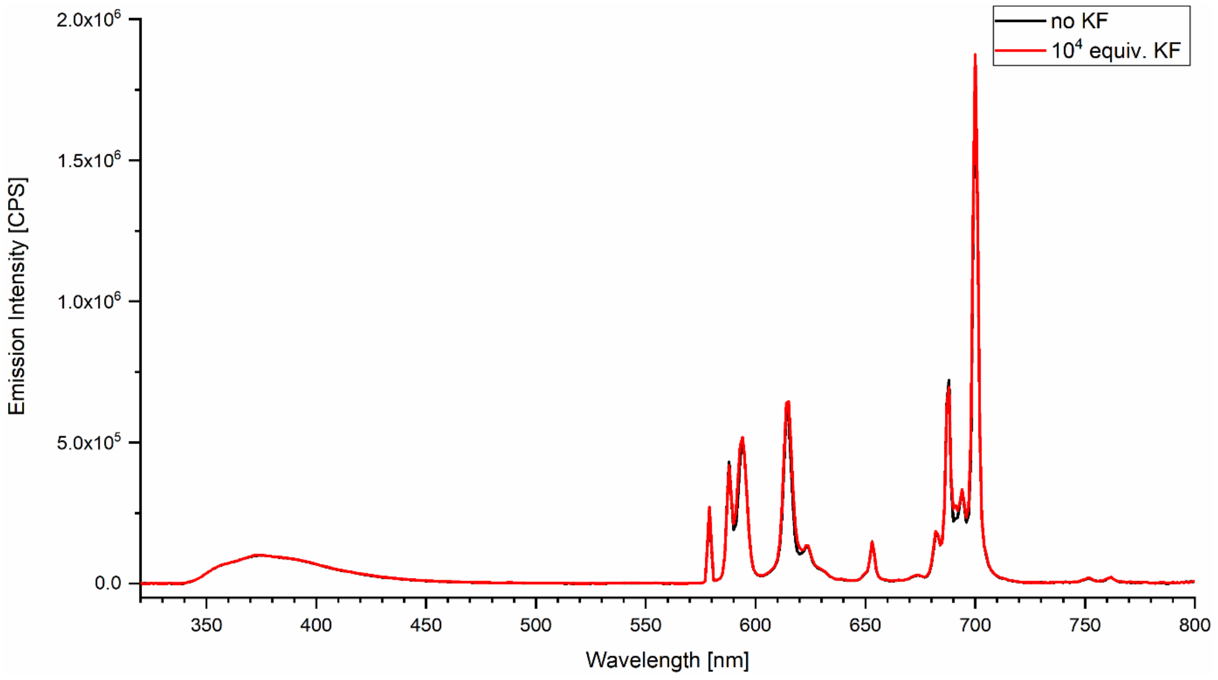

Figure S63. Steady-state emission spectra of **EuL1a^{Car}** with and without added excess of KF showing relative emission intensities under similar sample absorbance. [**EuL1a^{Car}**] = 10 µM in 10 mM aqueous PIPES buffer at pH 6.5, λ_{ex} = 327 nm, front slits: 2 nm, exit slits: 1.5 nm.

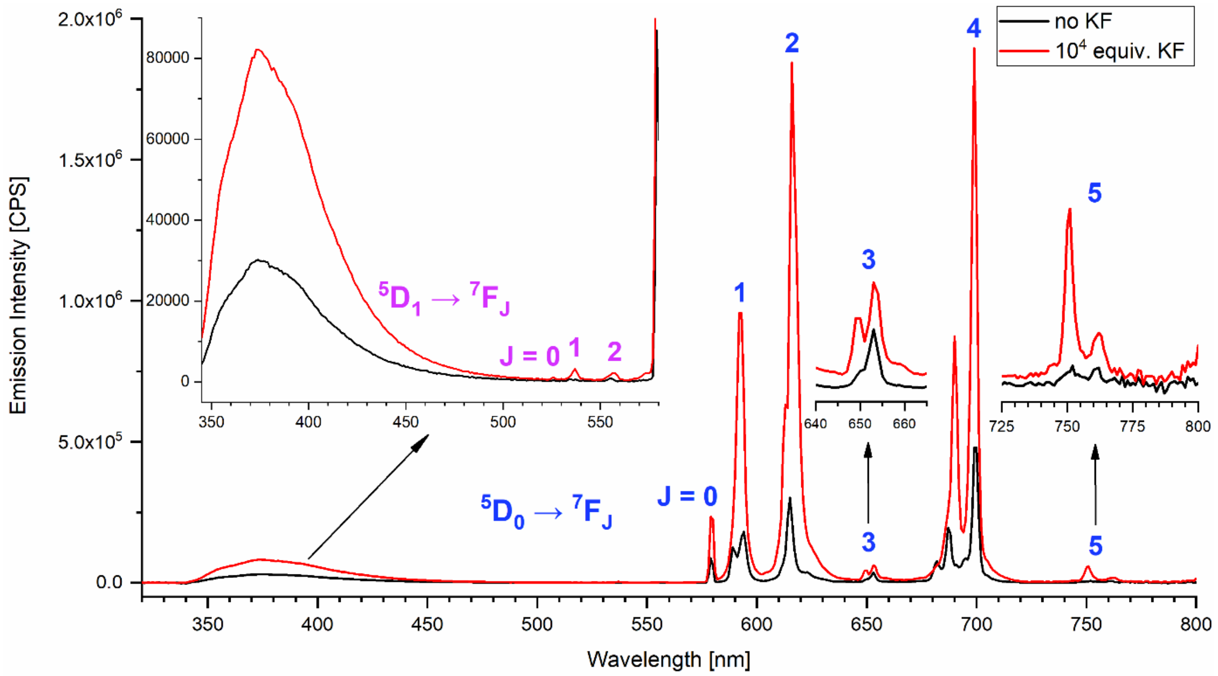

Figure S64. Steady-state emission spectra of **EuL2a^{Car}** with and without added excess of KF showing relative emission intensities under similar sample absorbance. [**EuL2a^{Car}**] = 10 µM in 10 mM aqueous PIPES buffer at pH 6.5, λ_{ex} = 327 nm, front slits: 2 nm, exit slits: 1.5 nm.

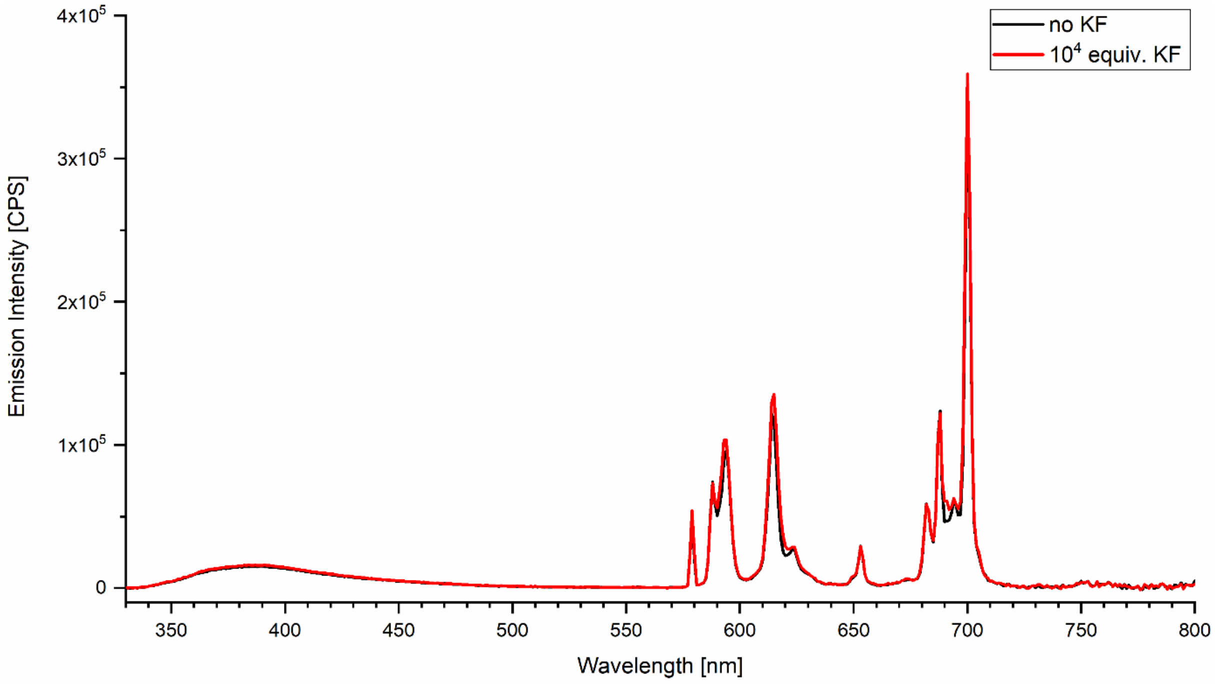

Figure S65. Steady-state emission spectra of **EuL1d^{Cou}** with and without added excess of KF showing relative emission intensities under similar sample absorbance. [**EuL1d^{Cou}**] = 10 µM in 10 mM aqueous PIPES buffer at pH 6.5, λ_{ex} = 315 nm, front slits: 2 nm, exit slits: 1.5 nm.

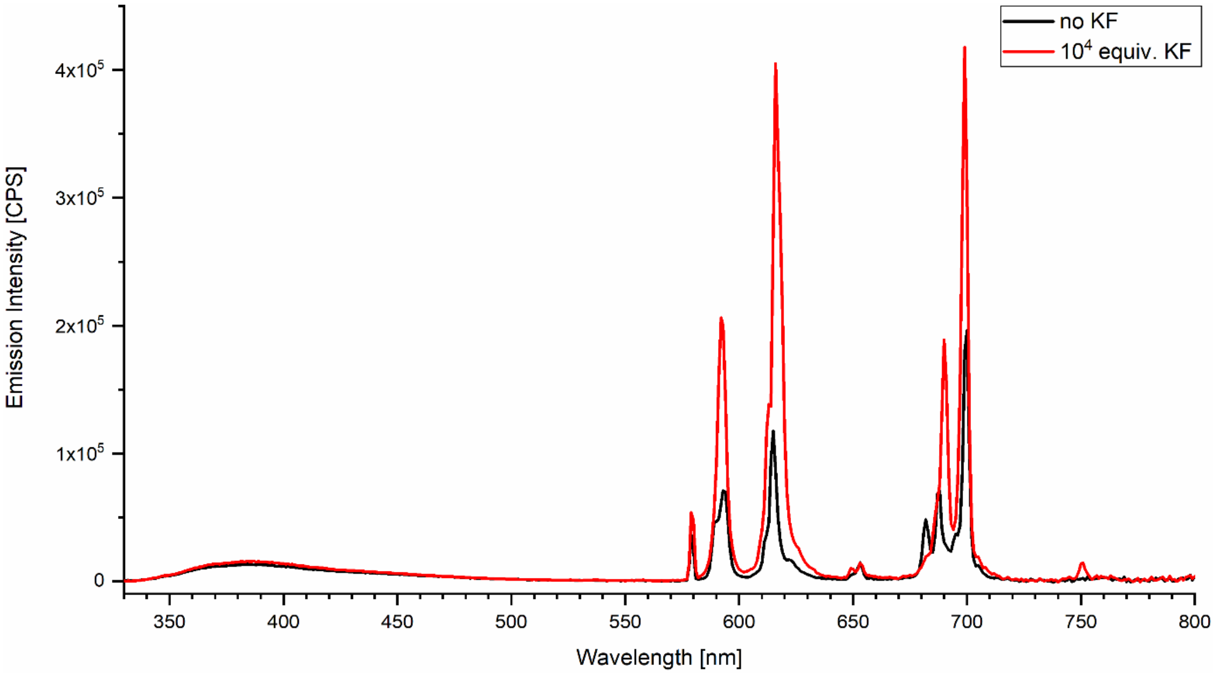

Figure S66. Steady-state emission spectra of **EuL2d^{Cou}** with and without added excess of KF showing relative emission intensities under similar sample absorbance. [**EuL2d^{Cou}**] = 10 µM in 10 mM aqueous PIPES buffer at pH 6.5, λ_{ex} = 315 nm, front slits: 2 nm, exit slits: 1.5 nm.

Table S30. Antenna and Sm(III)-based luminescence quantum yields and Sm(III) lifetimes of **SmL-F**. In parentheses fold increase compared to **SmL** without added KF. [a]

Complex	Φ_L [%] [b]	Φ_{Ln} [%] [b]	τ_{H2O} [ms]	τ_{D2O} [ms]
**SmL1a^{Car}–F**	3.6	0.19	0.010	0.032
**SmL2a^{Car}–F**	3.5	0.23 (×1.1)	0.014	0.041
**SmL1d^{Cou}–F**	0.62	0.045	0.010	0.032
**SmL2d^{Cou}–F**	0.66	0.074 (×1.1)	0.014	0.041

[a] Formed by the addition of excess KF (0.1 M, 10^4-fold excess) to a solution of **SmL**. [**SmL**] = 10 µM in 10 mM aqueous PIPES buffer solutions at pH 6.5. [b] Relative to quinine sulfate (Φ = 0.59) in H_2SO_4 (0.05 M) [4].

Table 31. Antenna and Ln(III)-based luminescence quantum yields of **SmL–F** in PIPES-buffered D_2O and changes relative to solutions in H_2O. [a]

Complex	Φ_L [%] [b]	Φ_{Ln} [%] [b]	$\frac{\Phi_{Ln,D2O}}{\Phi_{Ln,H2O}}$ [c]
**SmL1a^{Car}–F**	3.6	0.65	3.4
**SmL2a^{Car}–F**	3.5	0.77	3.3
**SmL1d^{Cou}–F**	0.67	0.19	4.2
**SmL2d^{Cou}–F**	0.73	0.22	3.0

[a] Formed by the addition of excess KF (0.1 M, 10^4-fold excess) to a solution of **SmL**. [**SmL**] = 10 µM in 10 mM PIPES buffer solutions in D_2O at pD 6.5 at nominally 10 µM concentrations. [b] Relative to quinine sulfate (Φ = 0.59) in H_2SO_4 (0.05 M) [4]. [c] Fold increase relative to the solution in H_2O.

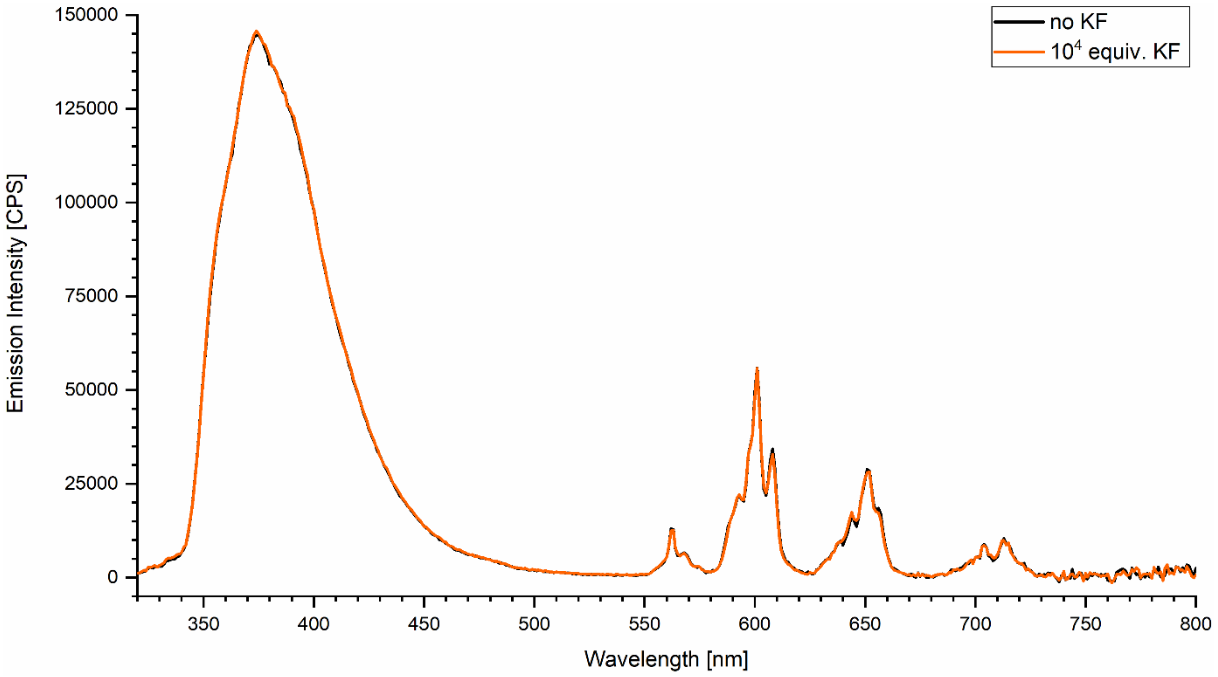

Figure S67. Steady-state emission spectra of **SmL1a^{Car}** with and without added excess of KF showing relative emission intensities under similar sample absorbance. [**SmL1a^{Car}**] = 10 µM in 10 mM D_2O PIPES buffer at pD 6.5, λ_{ex} = 327 nm, front slits: 2 nm, exit slits: 1.5 nm.

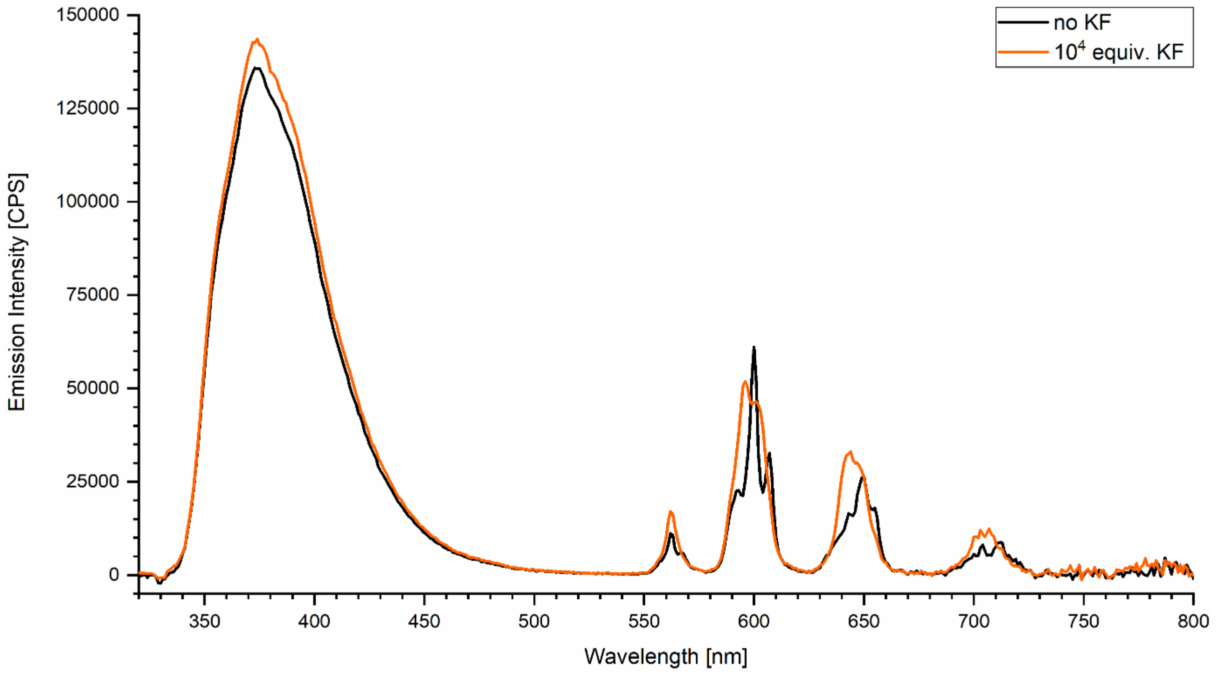

Figure S68. Steady-state emission spectra of **SmL2a^{Car}** with and without added excess of KF showing relative emission intensities under similar sample absorbance. [**SmL2a^{Car}**] = 10 µM in 10 mM D_2O PIPES buffer at pD 6.5, λ_{ex} = 327 nm, front slits: 2 nm, exit slits: 1.5 nm.

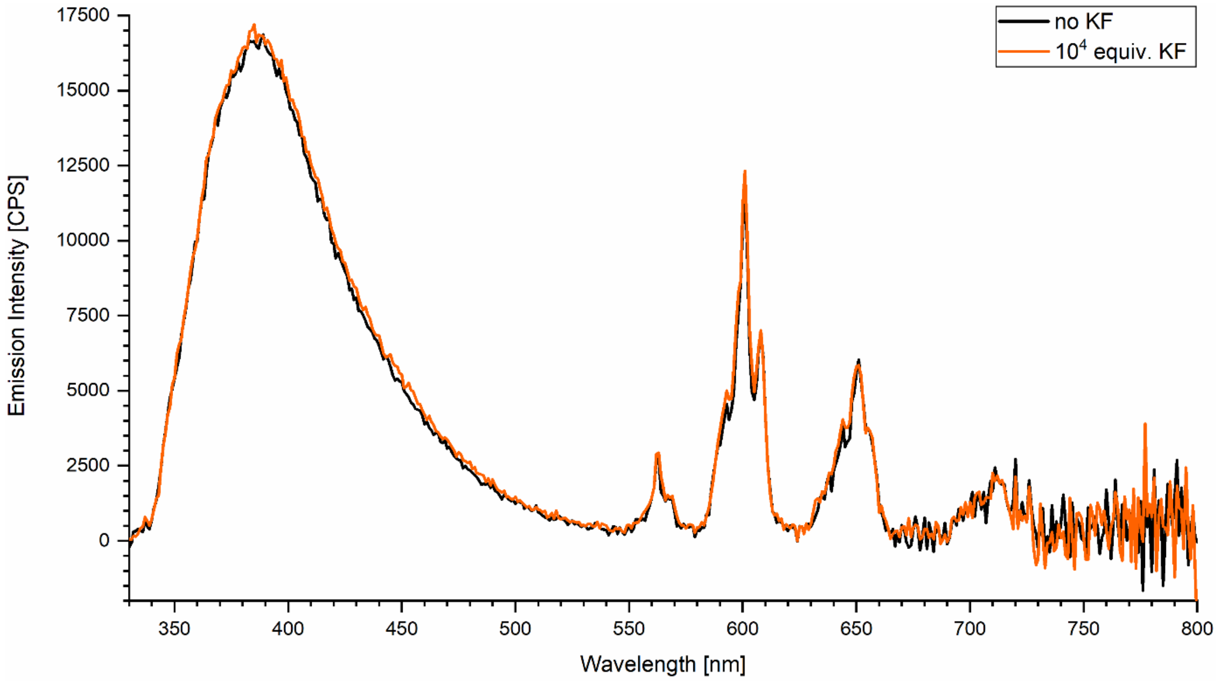

Figure S69. Steady-state emission spectra of **SmL1d**$^{\mathbf{Cou}}$ with and without added excess of KF showing relative emission intensities under similar sample absorbance. [**SmL1d**$^{\mathbf{Cou}}$] = 10 µM in 10 mM D_2O PIPES buffer at pD 6.5, λ_{ex} = 315 nm, front slits: 2 nm, exit slits: 1.5 nm.

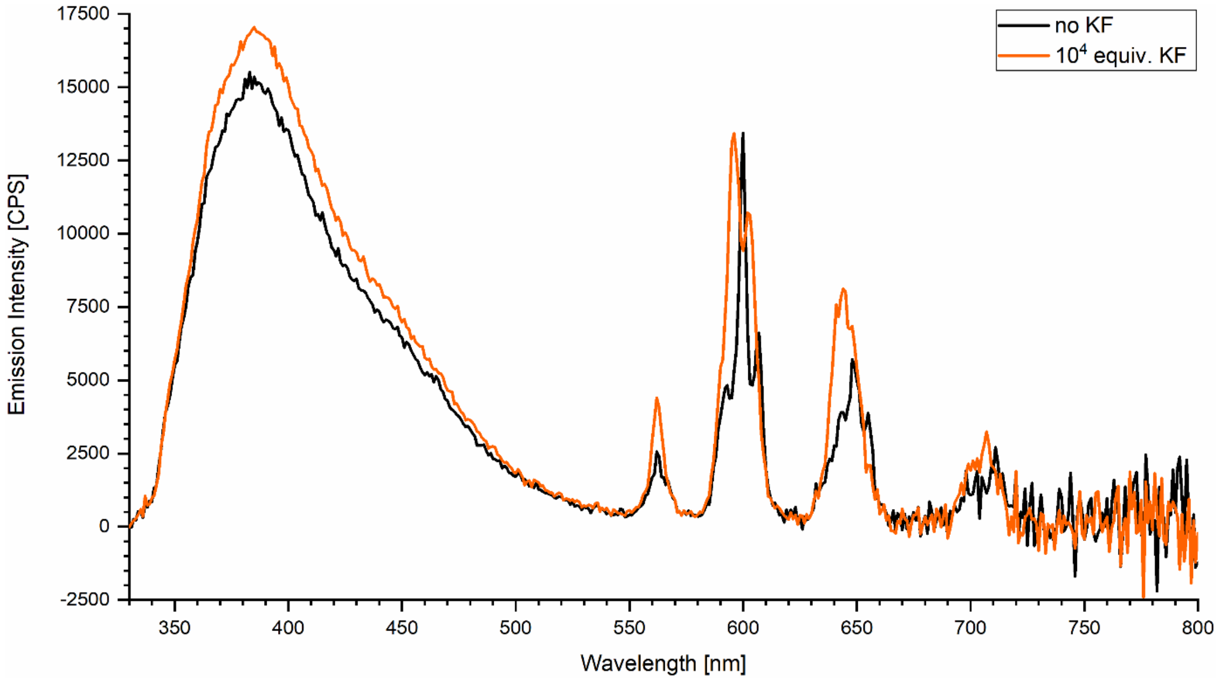

Figure S70. Steady-state emission spectra of **SmL2d**$^{\mathbf{Cou}}$ with and without added excess of KF showing relative emission intensities under similar sample absorbance. [**SmL2d**$^{\mathbf{Cou}}$] = 10 µM in 10 mM D_2O PIPES buffer at pD 6.5, λ_{ex} = 315 nm, front slits: 2 nm, exit slits: 1.5 nm.

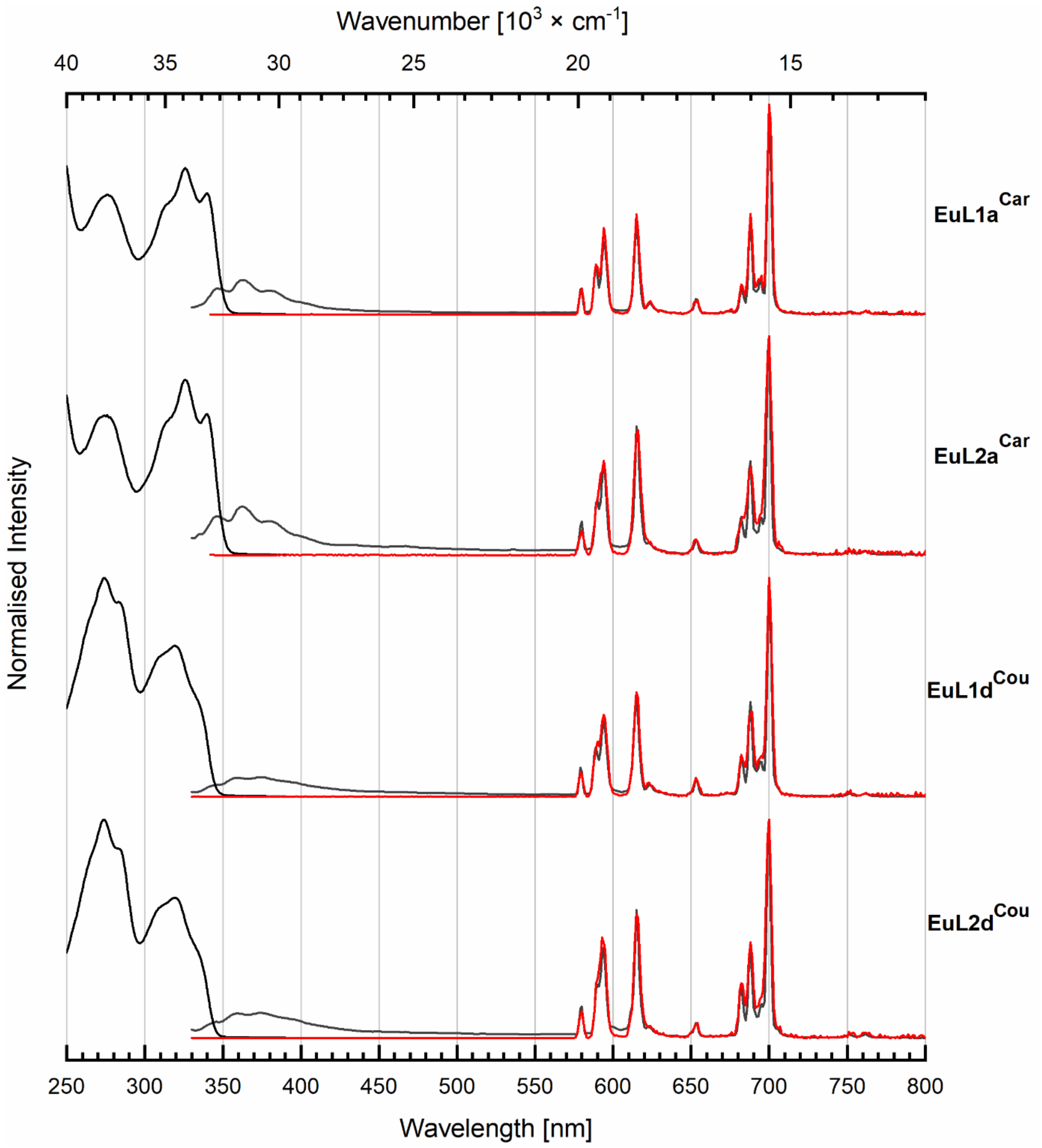

Figure S71. Excitation of Eu(III) luminescence (black, λ_{em} = 615 nm), steady-state (dark gray) and time-resolved (red) emission spectra of **EuL** complexes at 77 K. [**EuL**] = 10 µM with 10% glycerol in aqueous 10 mM PIPES buffer solutions at pH 6.5 with λ_{ex} = 315 nm (**EuL^{Cou}**) and 327 nm (**EuL^{Car}**).

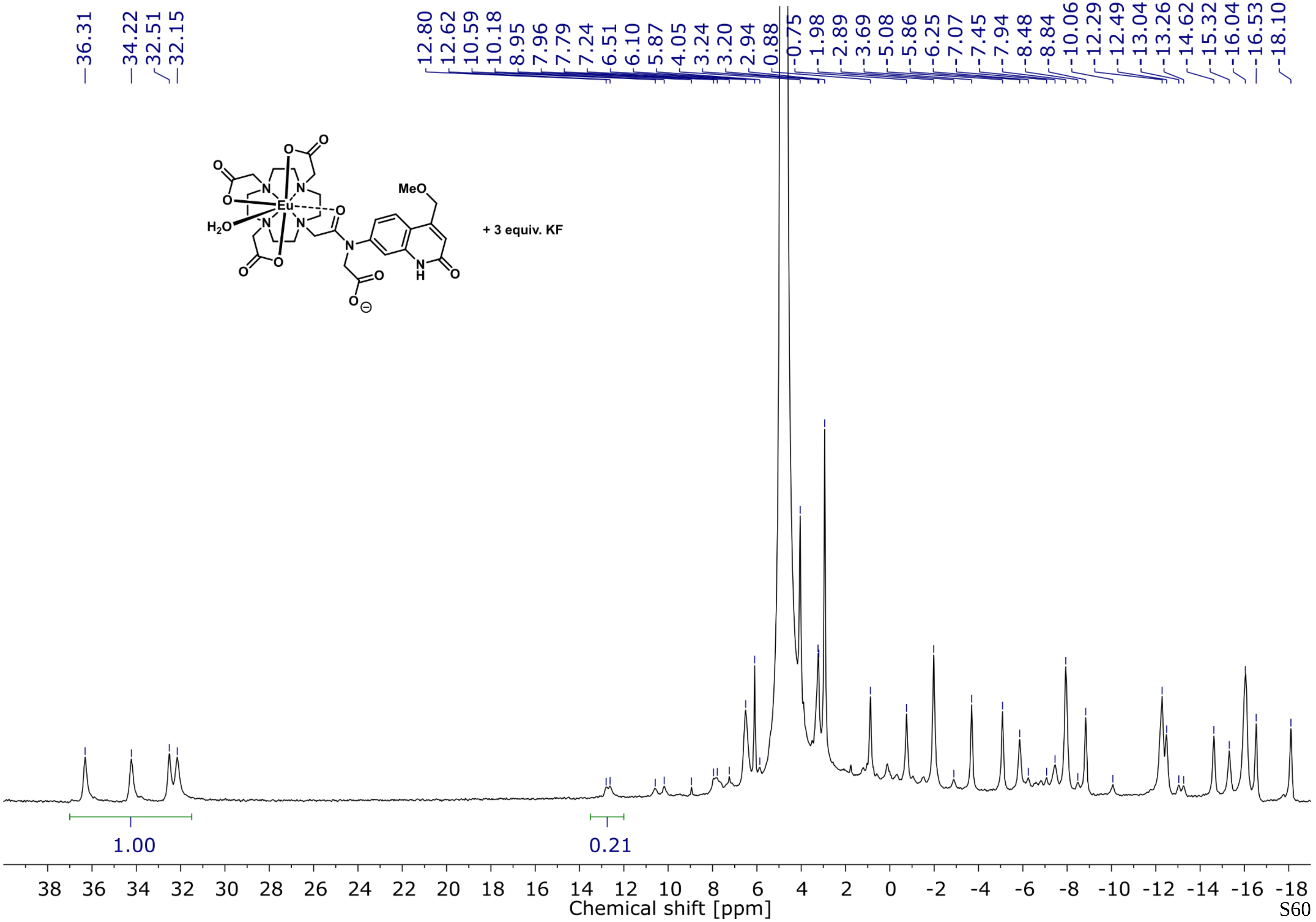
36.31
34.22
32.51
32.15
12.80
12.62
10.59
10.18
8.95
7.96
7.79
7.24
6.51
6.10
5.87
4.05
3.24
3.20
2.94
0.88
-0.75
-1.98
-2.89
-3.69
-5.08
-5.86
-6.25
-7.07
-7.45
-7.94
-8.48
-8.84
-10.06
-12.29
-12.49
-13.04
-13.26
-14.62
-15.32
-16.04
-16.53
-18.10
+ 3 equiv. KF
1.00
0.21
38 36 34 32 30 28 26 24 22 20 18 16 14 12 10 8 6 4 2 0 -2 -4 -6 -8 -10 -12 -14 -16 -18
Chemical shift [ppm]

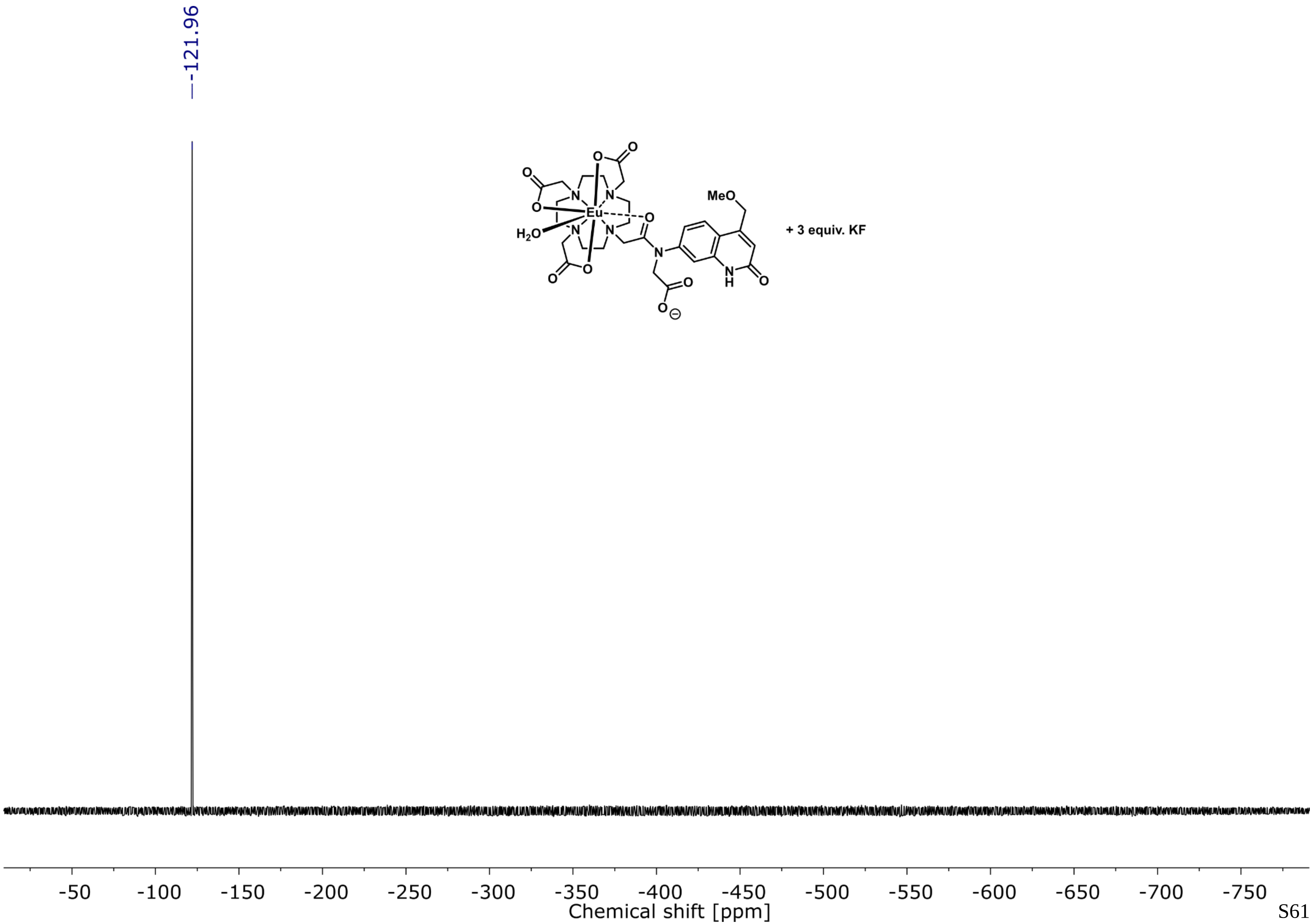
-121.96
+ 3 equiv. KF
MeO
Eu
H_2O
-50
-100
-150
-200
-250
-300
-350
-400
-450
-500
-550
-600
-650
-700
-750
Chemical shift [ppm]

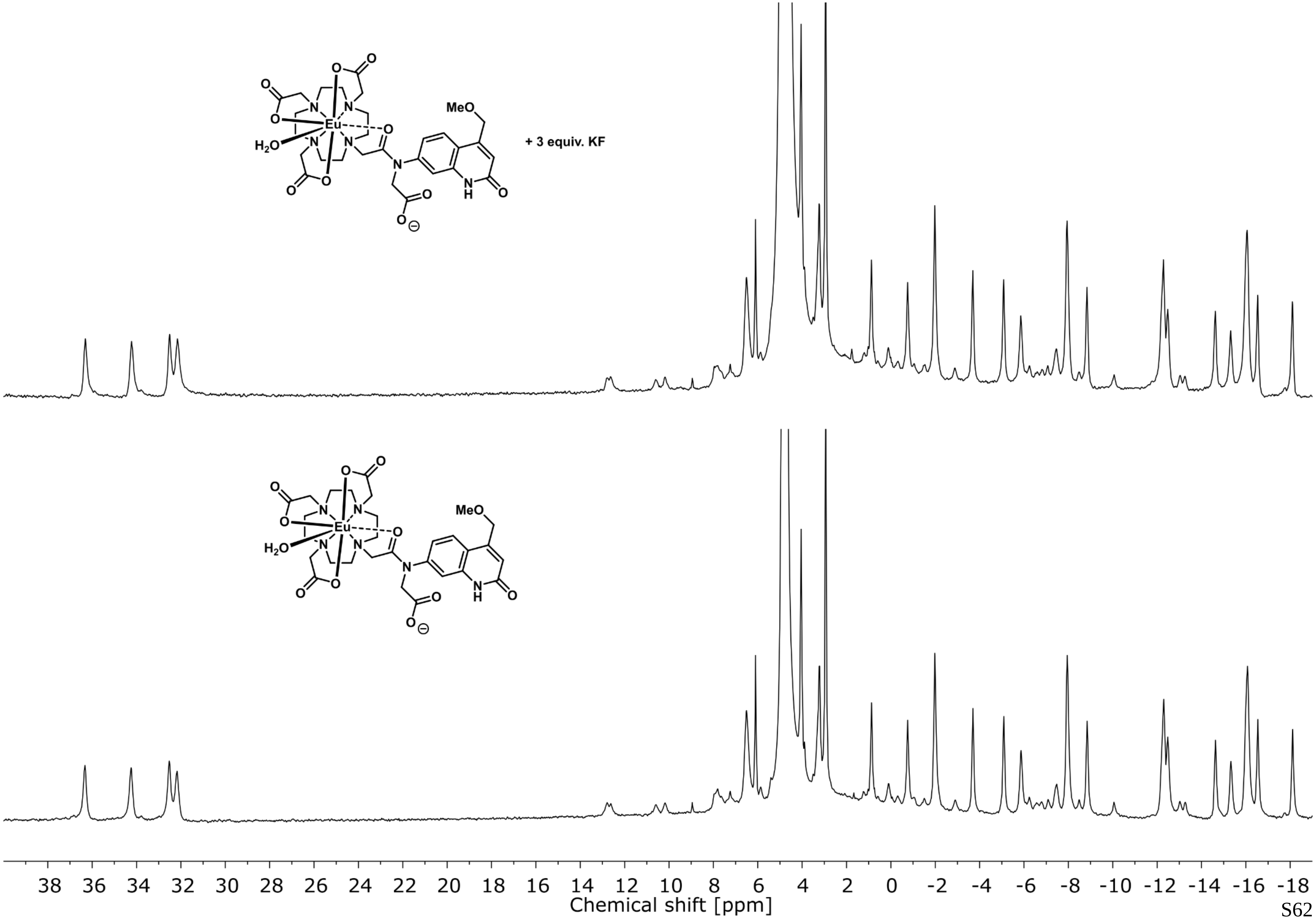

MeO
Eu
H2O
N
H
+ 3 equiv. KF
MeO
Eu
H2O
N
H
38 36 34 32 30 28 26 24 22 20 18 16 14 12 10 8 6 4 2 0 -2 -4 -6 -8 -10 -12 -14 -16 -18
Chemical shift [ppm]

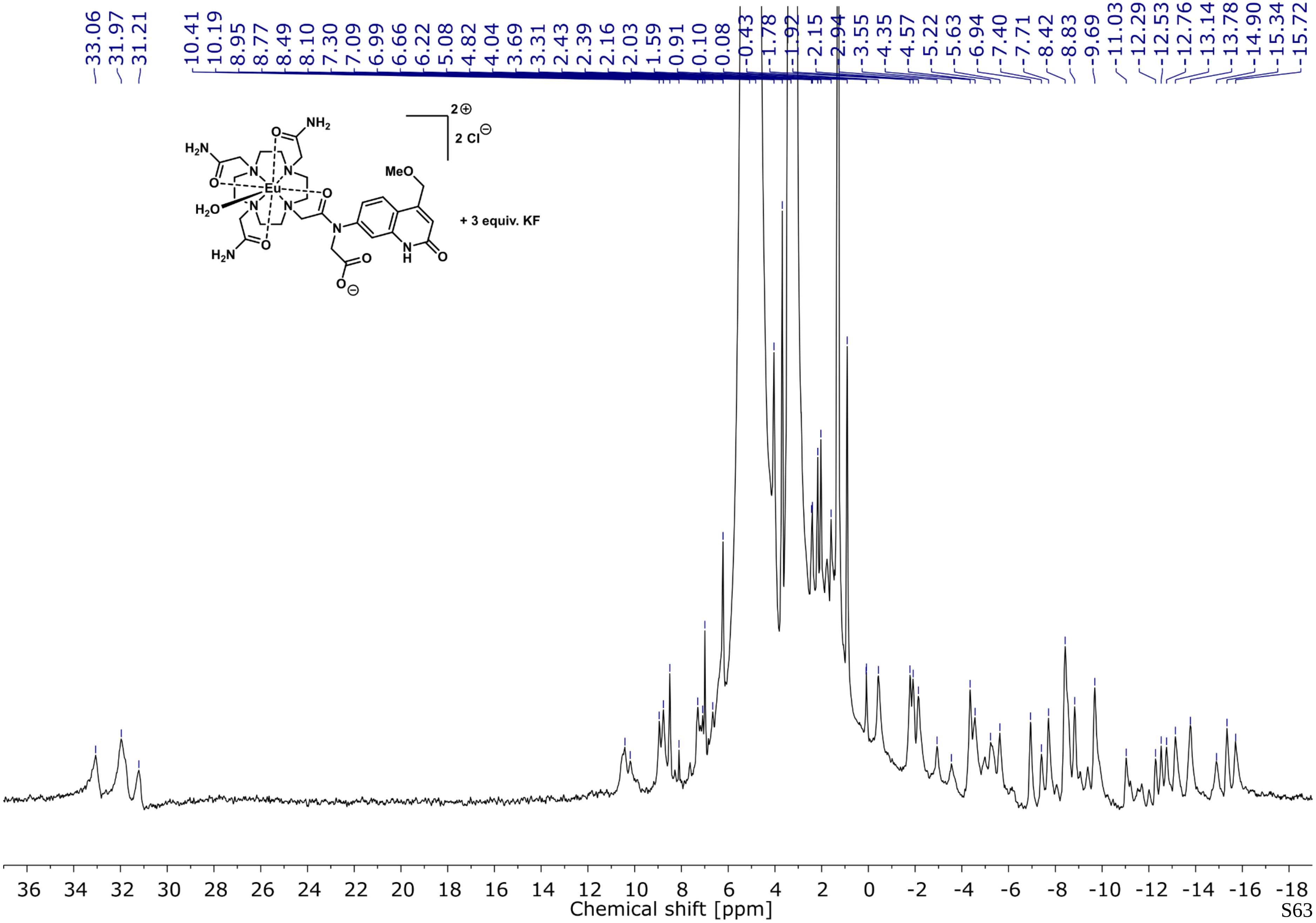
33.06
31.97
31.21
10.41
10.19
8.95
8.77
8.49
8.10
7.30
7.09
6.99
6.66
6.22
5.08
4.82
4.04
3.69
3.31
2.43
2.39
2.16
2.03
1.59
0.91
0.10
0.08
-0.43
-1.78
-1.92
-2.15
-2.94
-3.55
-4.35
-4.57
-5.22
-5.63
-6.94
-7.40
-7.71
-8.42
-8.83
-9.69
-11.03
-12.29
-12.53
-12.76
-13.14
-13.78
-14.90
-15.34
-15.72
2⊕
2 Cl⊖
+ 3 equiv. KF
Chemical shift [ppm]

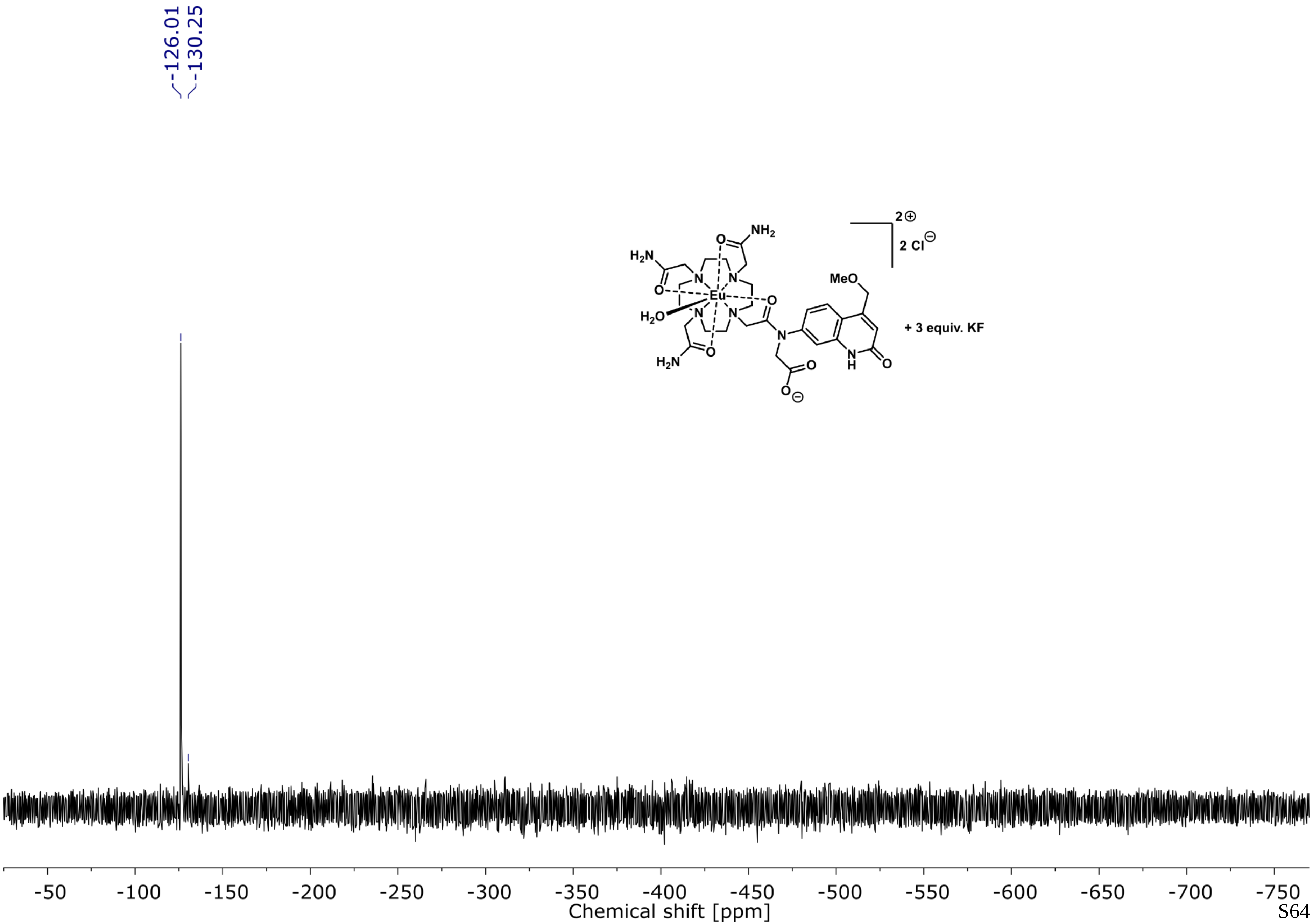
-126.01
-130.25
2+
2 Cl−
+ 3 equiv. KF
-50
-100
-150
-200
-250
-300
-350
-400
-450
-500
-550
-600
-650
-700
-750
Chemical shift [ppm]

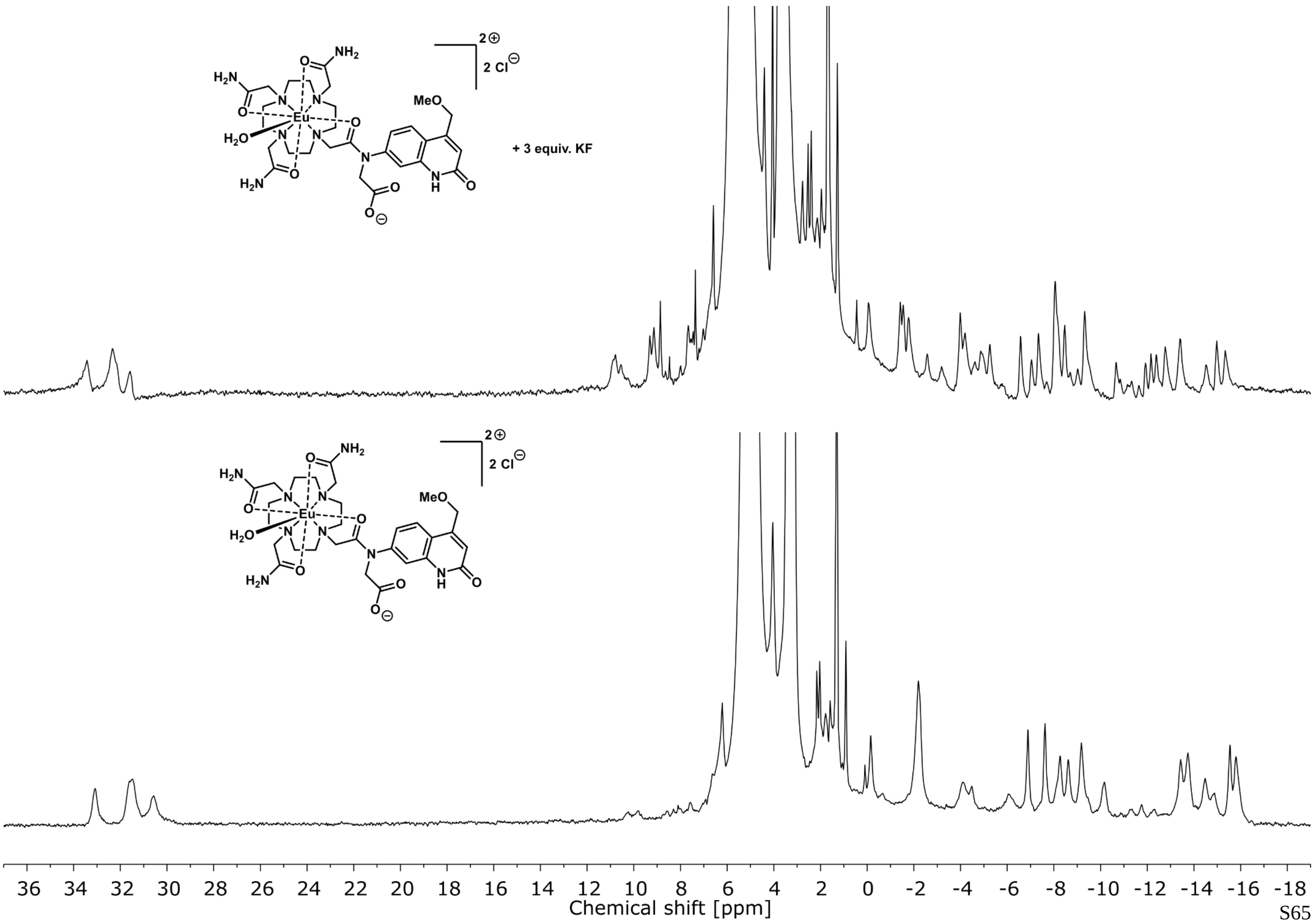

2⊕
2 Cl⊖
MeO
+ 3 equiv. KF
36 34 32 30 28 26 24 22 20 18 16 14 12 10 8 6 4 2 0 -2 -4 -6 -8 -10 -12 -14 -16 -18
Chemical shift [ppm]

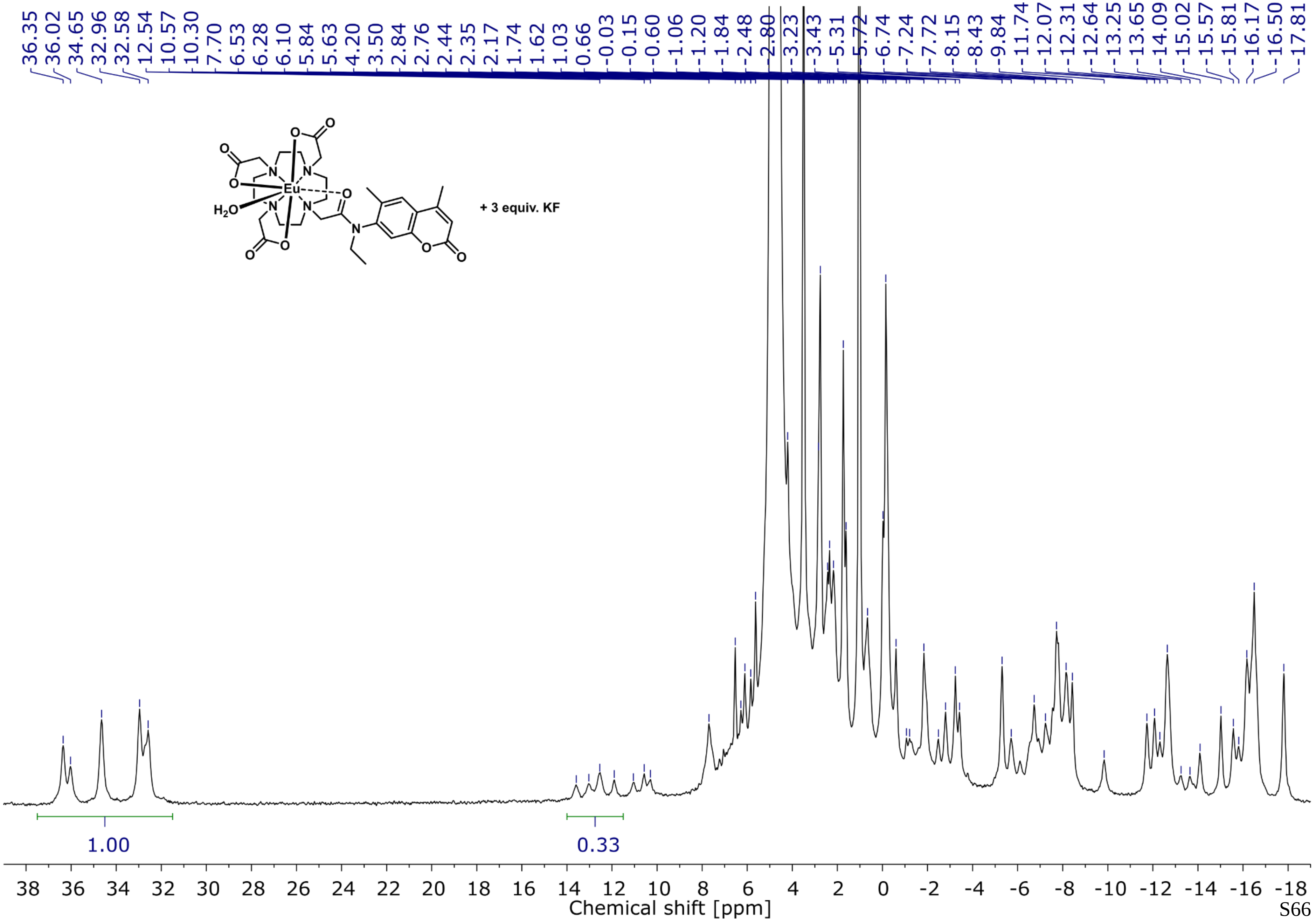
36.35
36.02
34.65
32.96
32.58
12.54
10.57
10.30
7.70
6.53
6.28
6.10
5.84
5.63
4.20
3.50
2.84
2.76
2.44
2.35
2.17
1.74
1.62
1.03
0.66
-0.03
-0.15
-0.60
-1.06
-1.20
-1.84
-2.48
-2.80
-3.23
-3.43
-5.31
-5.72
-6.74
-7.24
-7.72
-8.15
-8.43
-9.84
-11.74
-12.07
-12.31
-12.64
-13.25
-13.65
-14.09
-15.02
-15.57
-15.81
-16.17
-16.50
-17.81
+ 3 equiv. KF
1.00
0.33
Chemical shift [ppm]

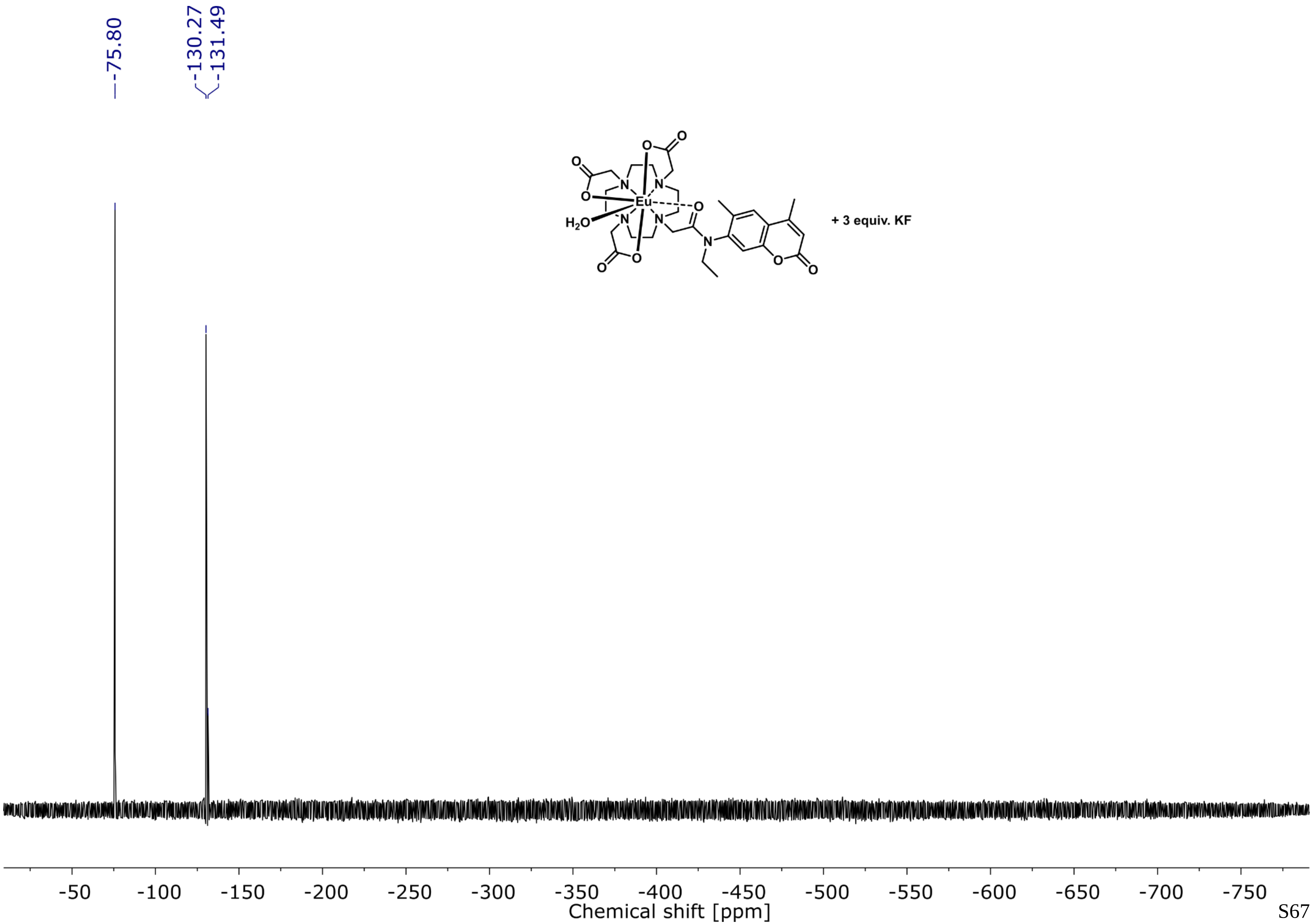
-75.80
-130.27
-131.49
+ 3 equiv. KF
Chemical shift [ppm]

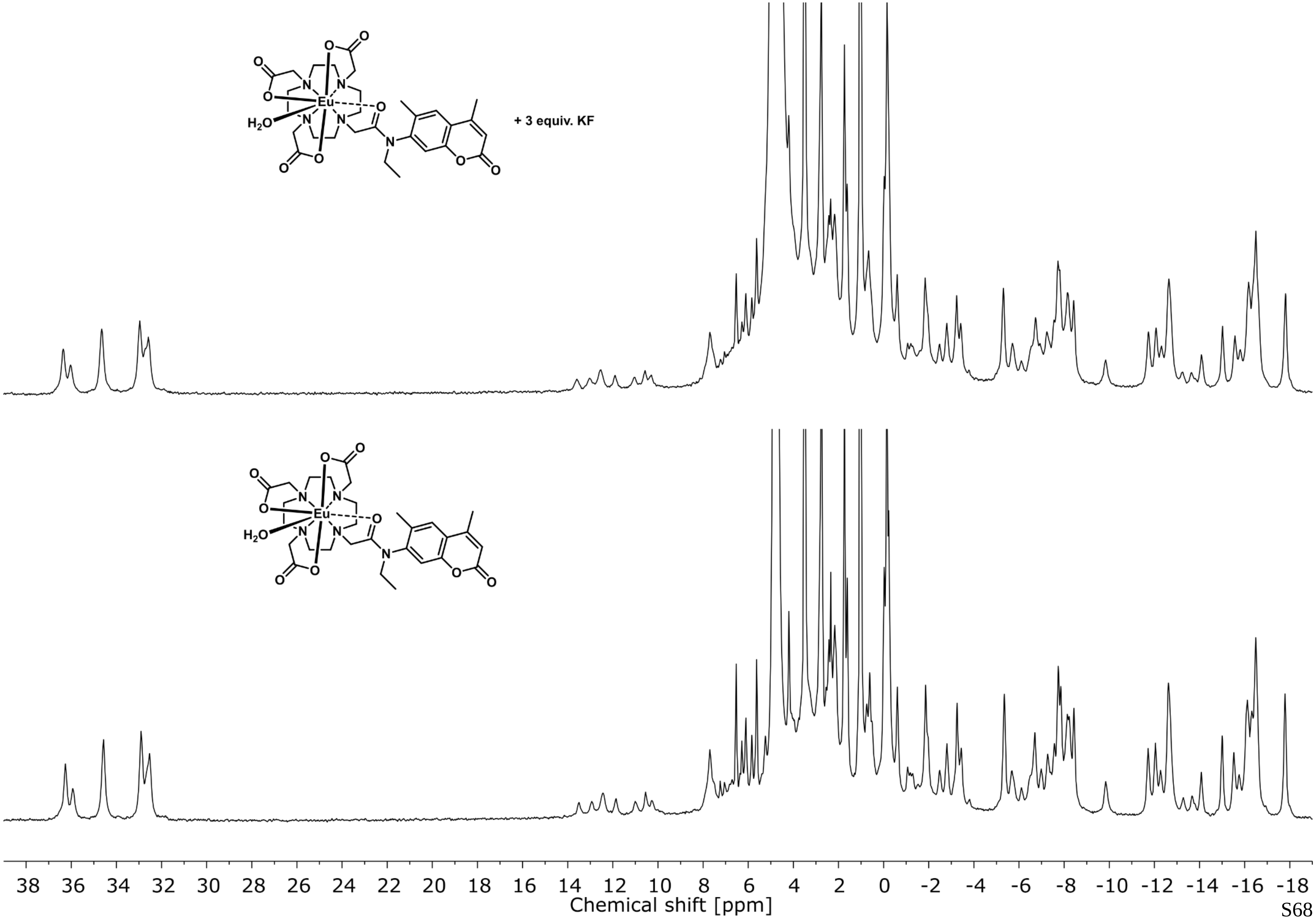
+ 3 equiv. KF
38 36 34 32 30 28 26 24 22 20 18 16 14 12 10 8 6 4 2 0 -2 -4 -6 -8 -10 -12 -14 -16 -18
Chemical shift [ppm]

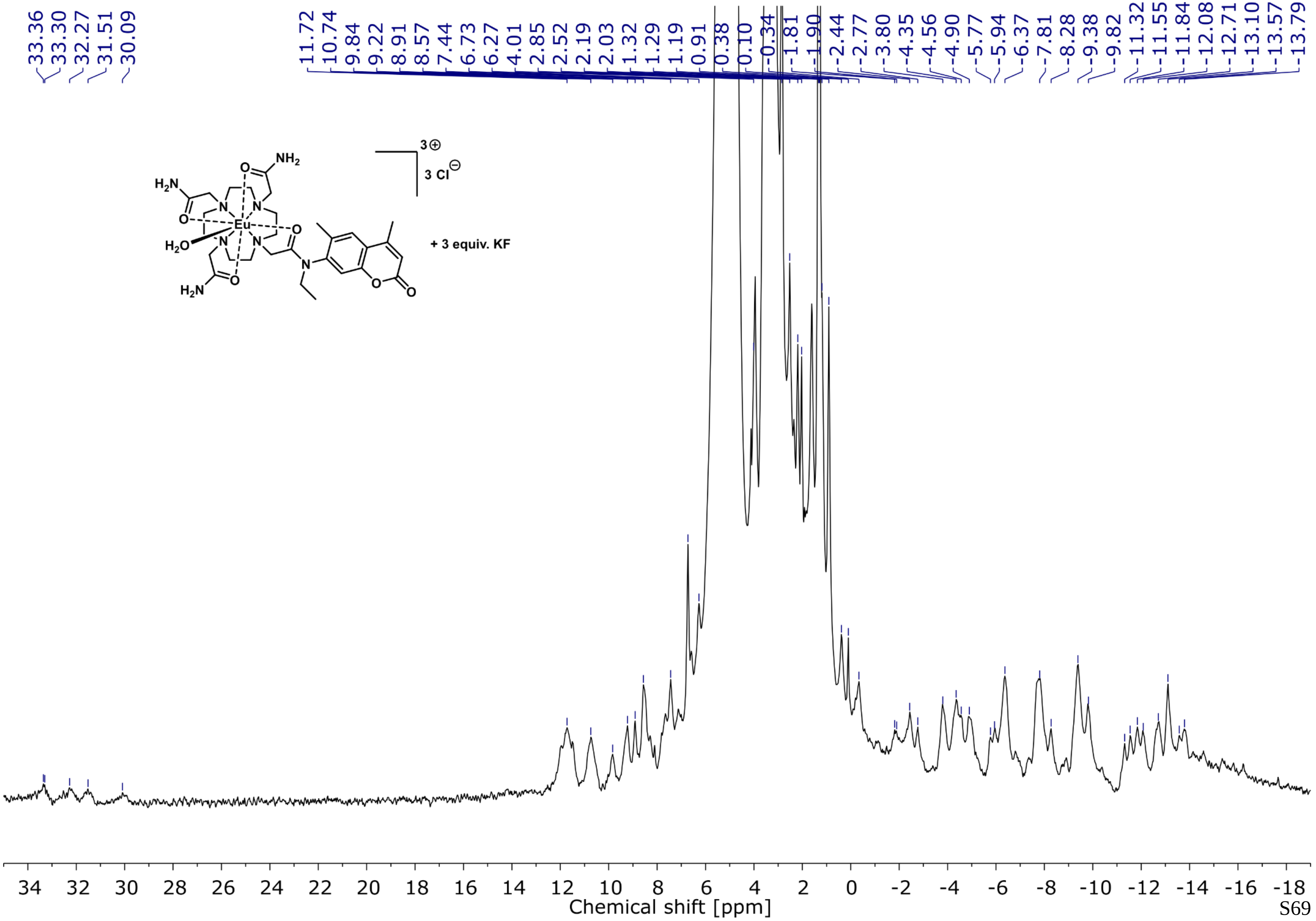
33.36
33.30
32.27
31.51
30.09
11.72
10.74
9.84
9.22
8.91
8.57
7.44
6.73
6.27
4.01
2.85
2.52
2.19
2.03
1.32
1.29
1.19
0.91
0.38
0.10
-0.34
-1.81
-1.90
-2.44
-2.77
-3.80
-4.35
-4.56
-4.90
-5.77
-5.94
-6.37
-7.81
-8.28
-9.38
-9.82
-11.32
-11.55
-11.84
-12.08
-12.71
-13.10
-13.57
-13.79
3⊕
3 Cl⊖
+ 3 equiv. KF
Chemical shift [ppm]

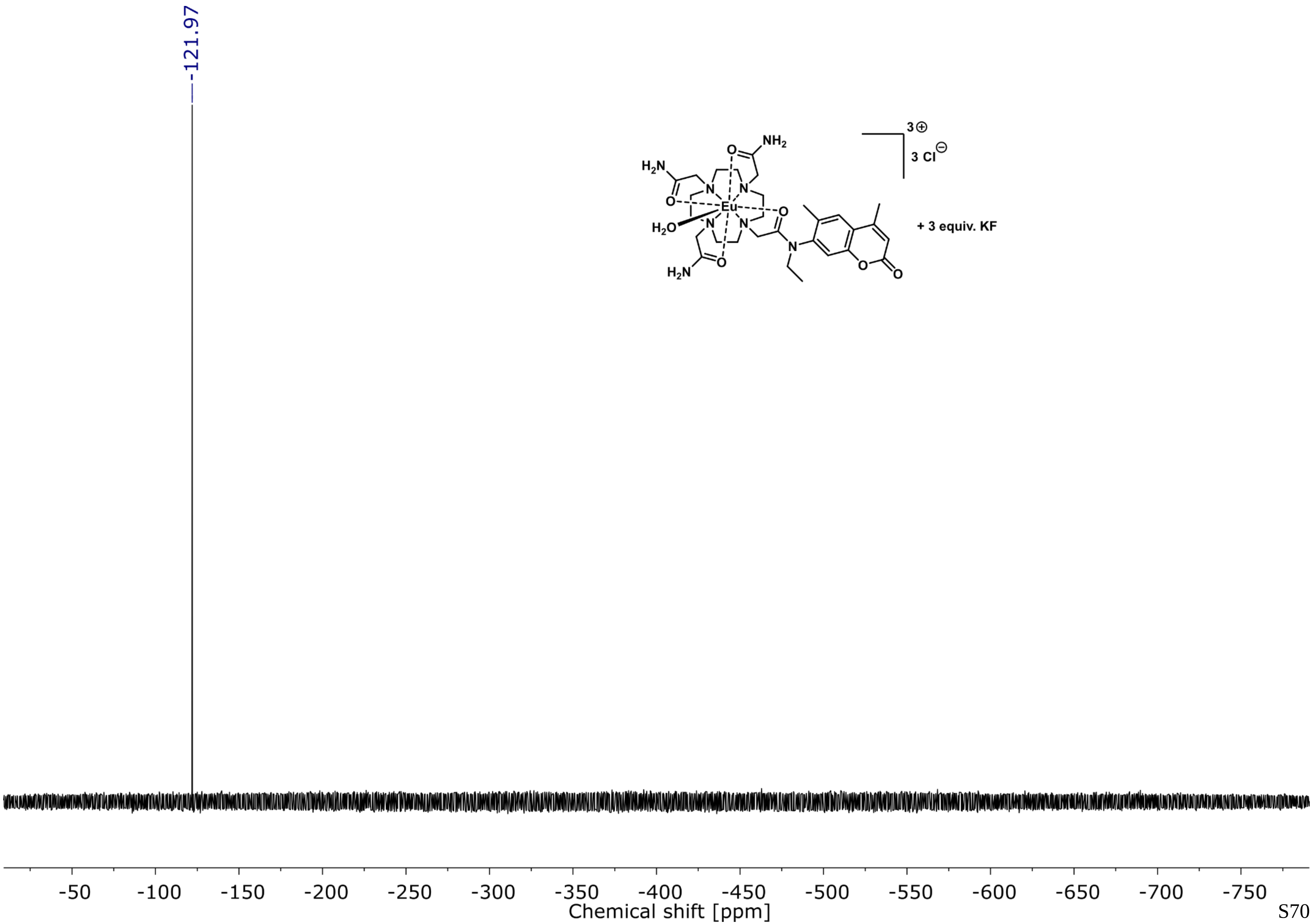
-121.97
3 Cl
+ 3 equiv. KF
-50 -100 -150 -200 -250 -300 -350 -400 -450 -500 -550 -600 -650 -700 -750
Chemical shift [ppm]

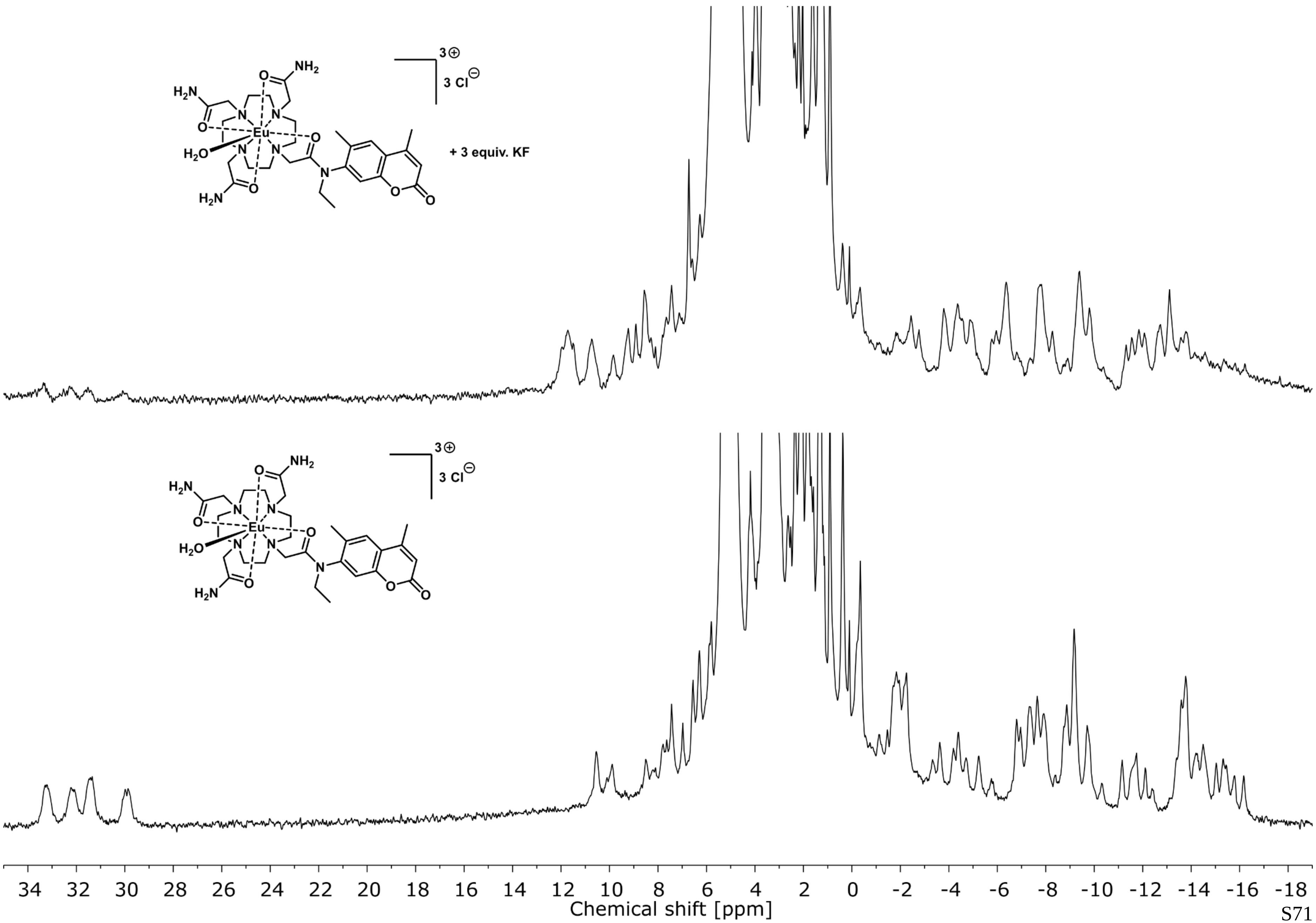
+ 3 equiv. KF
3 Cl
34 32 30 28 26 24 22 20 18 16 14 12 10 8 6 4 2 0 -2 -4 -6 -8 -10 -12 -14 -16 -18
Chemical shift [ppm]

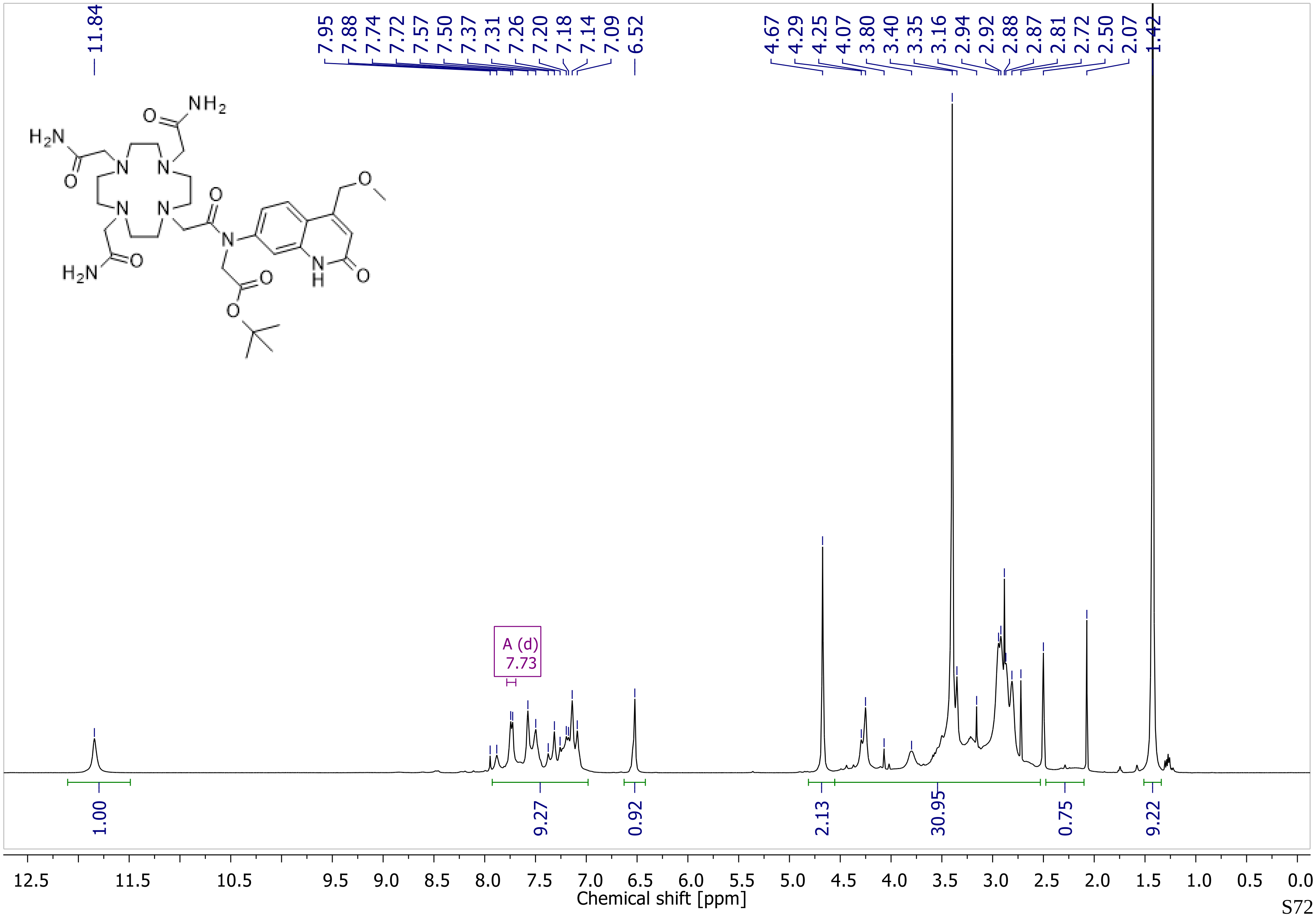

11.84
7.95 7.88 7.74 7.72 7.57 7.50 7.37 7.31 7.26 7.20 7.18 7.14 7.09
6.52
4.67 4.29 4.25 4.07 3.80 3.40 3.35 3.16 2.94 2.92 2.88 2.87 2.81 2.72 2.50 2.07
1.42
NH2
H2N
O
N
O
H
A (d)
7.73
1.00
9.27
0.92
2.13
30.95
0.75
9.22
12.5 11.5 10.5 9.5 9.0 8.5 8.0 7.5 7.0 6.5 6.0 5.5 5.0 4.5 4.0 3.5 3.0 2.5 2.0 1.5 1.0 0.5 0.0
Chemical shift [ppm]

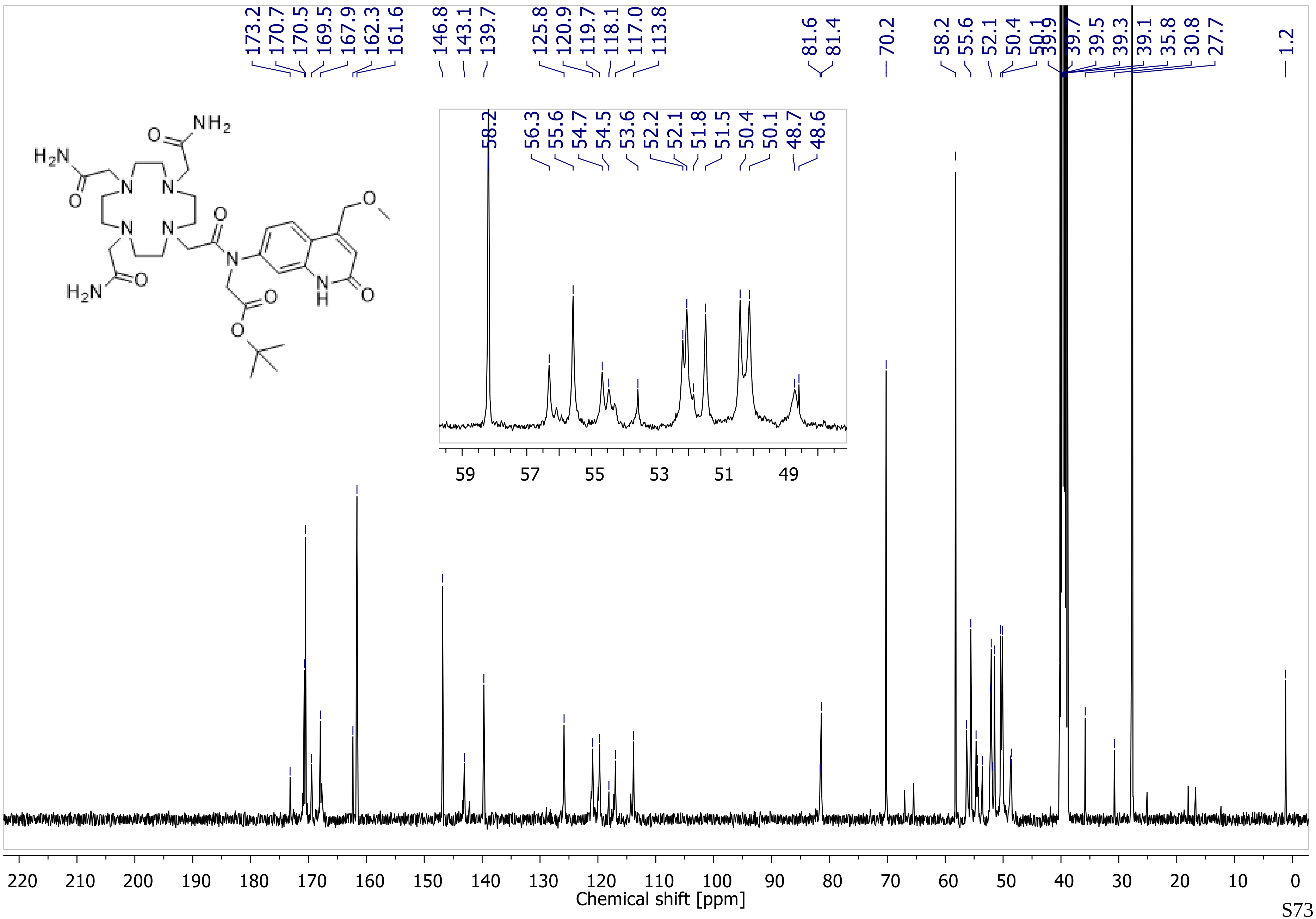
173.2
170.7
170.5
169.5
167.9
162.3
161.6
146.8
143.1
139.7
125.8
120.9
119.7
118.1
117.0
113.8
81.6
81.4
70.2
58.2
55.6
52.1
50.4
50.1
39.9
39.7
39.5
39.3
39.1
35.8
30.8
27.7
1.2
58.2
56.3
55.6
54.7
54.5
53.6
52.2
52.1
51.8
51.5
50.4
50.1
48.7
48.6
59 57 55 53 51 49
220 210 200 190 180 170 160 150 140 130 120 110 100 90 80 70 60 50 40 30 20 10 0
Chemical shift [ppm]

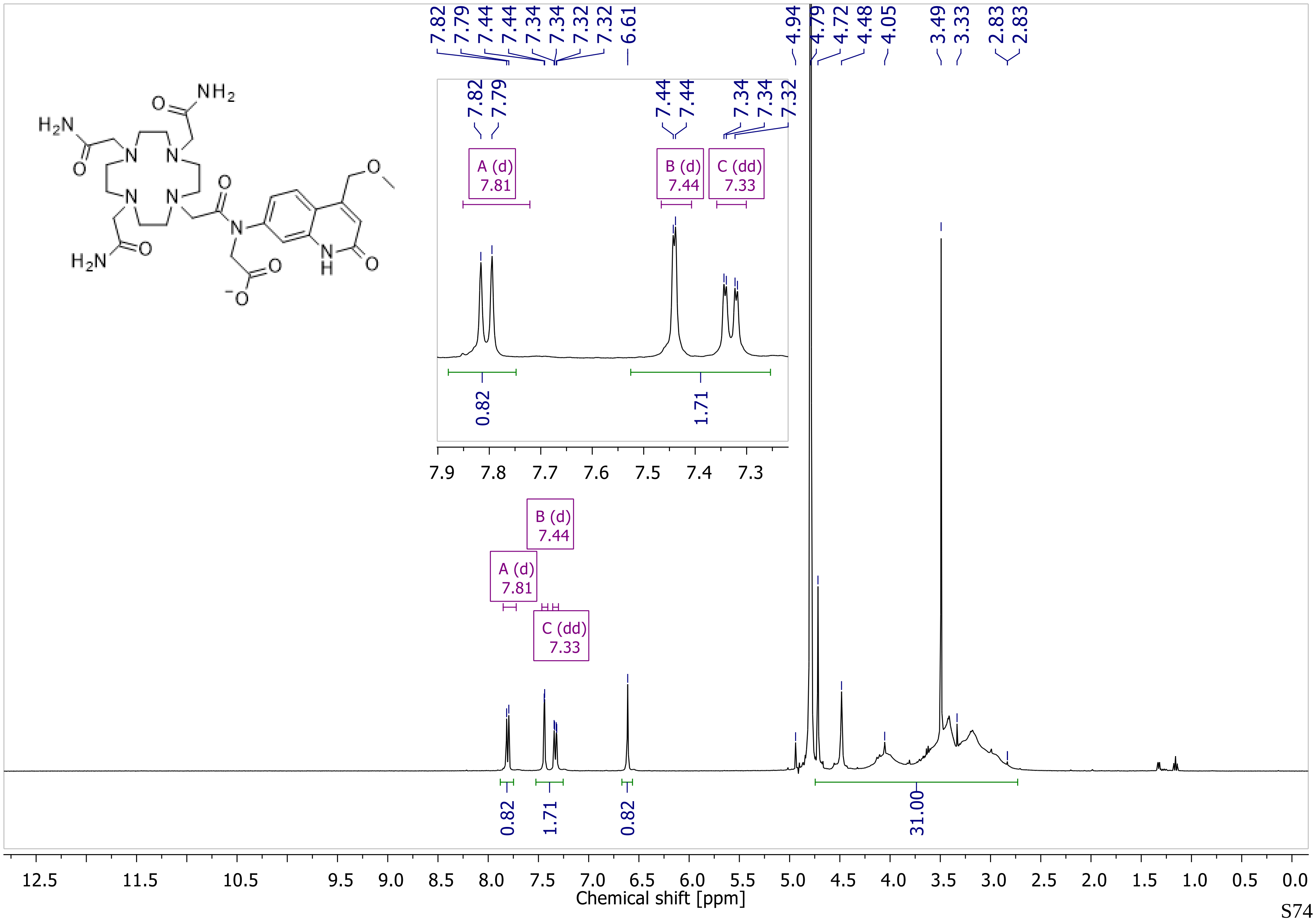

NH2
H2N
O
N
N
N
N
O
O
O
H2N
N
N
H
O
O
-O
7.82
7.79
7.44
7.44
7.34
7.34
7.32
7.32
6.61
4.94
4.79
4.72
4.48
4.05
3.49
3.33
2.83
2.83
A (d) 7.81
B (d) 7.44
C (dd) 7.33
0.82
1.71
0.82
31.00
7.9 7.8 7.7 7.6 7.5 7.4 7.3
12.5 11.5 10.5 9.5 9.0 8.5 8.0 7.5 7.0 6.5 6.0 5.5 5.0 4.5 4.0 3.5 3.0 2.5 2.0 1.5 1.0 0.5 0.0
Chemical shift [ppm]

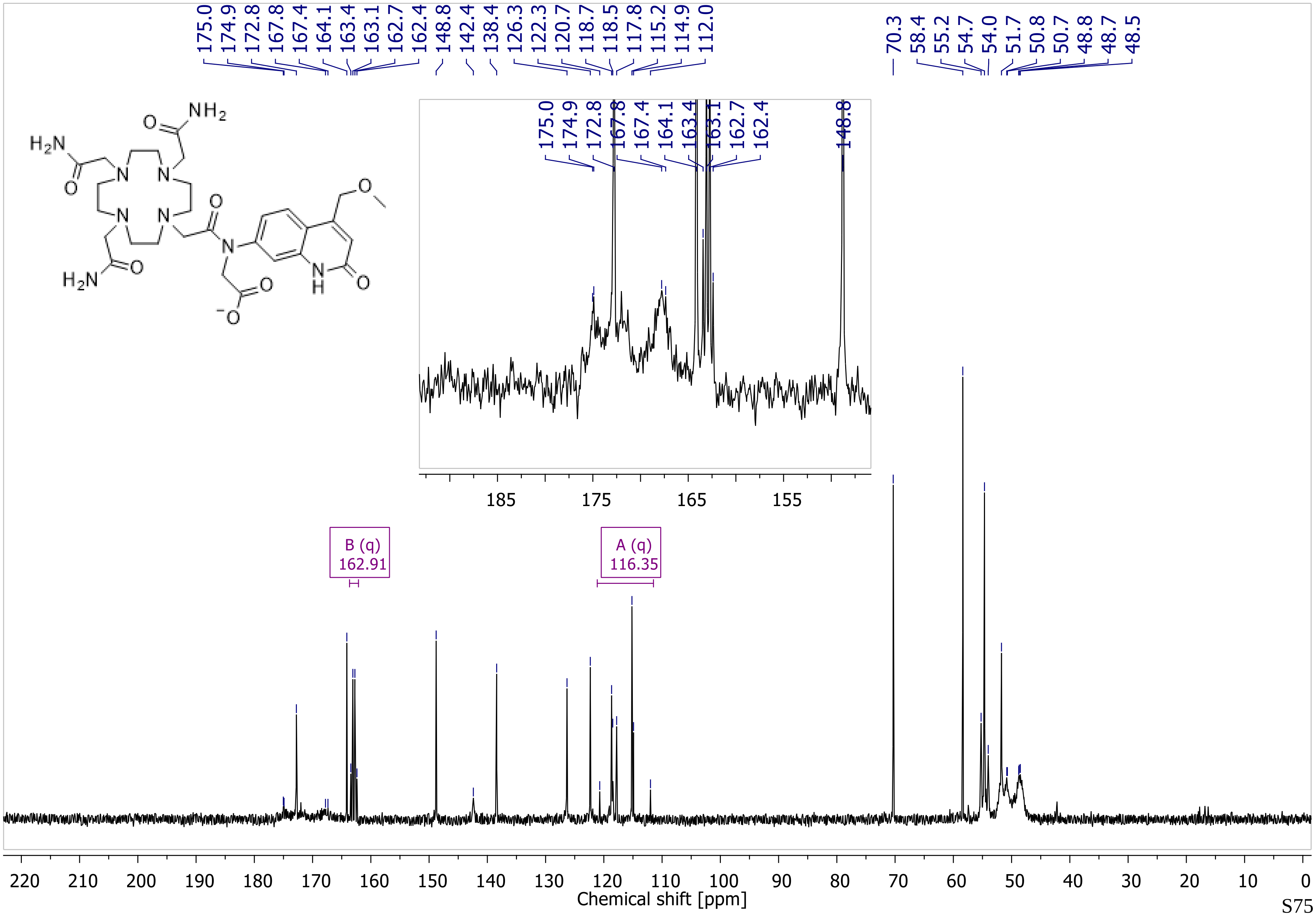

175.0 174.9 172.8 167.8 167.4 164.1 163.4 163.1 162.7 162.4 148.8 142.4 138.4 126.3 122.3 120.7 118.7 118.5 117.8 115.2 114.9 112.0
70.3 58.4 55.2 54.7 54.0 51.7 50.8 50.7 48.8 48.7 48.5
B (q) 162.91
A (q) 116.35
185 175 165 155
220 210 200 190 180 170 160 150 140 130 120 110 100 90 80 70 60 50 40 30 20 10 0
Chemical shift [ppm]

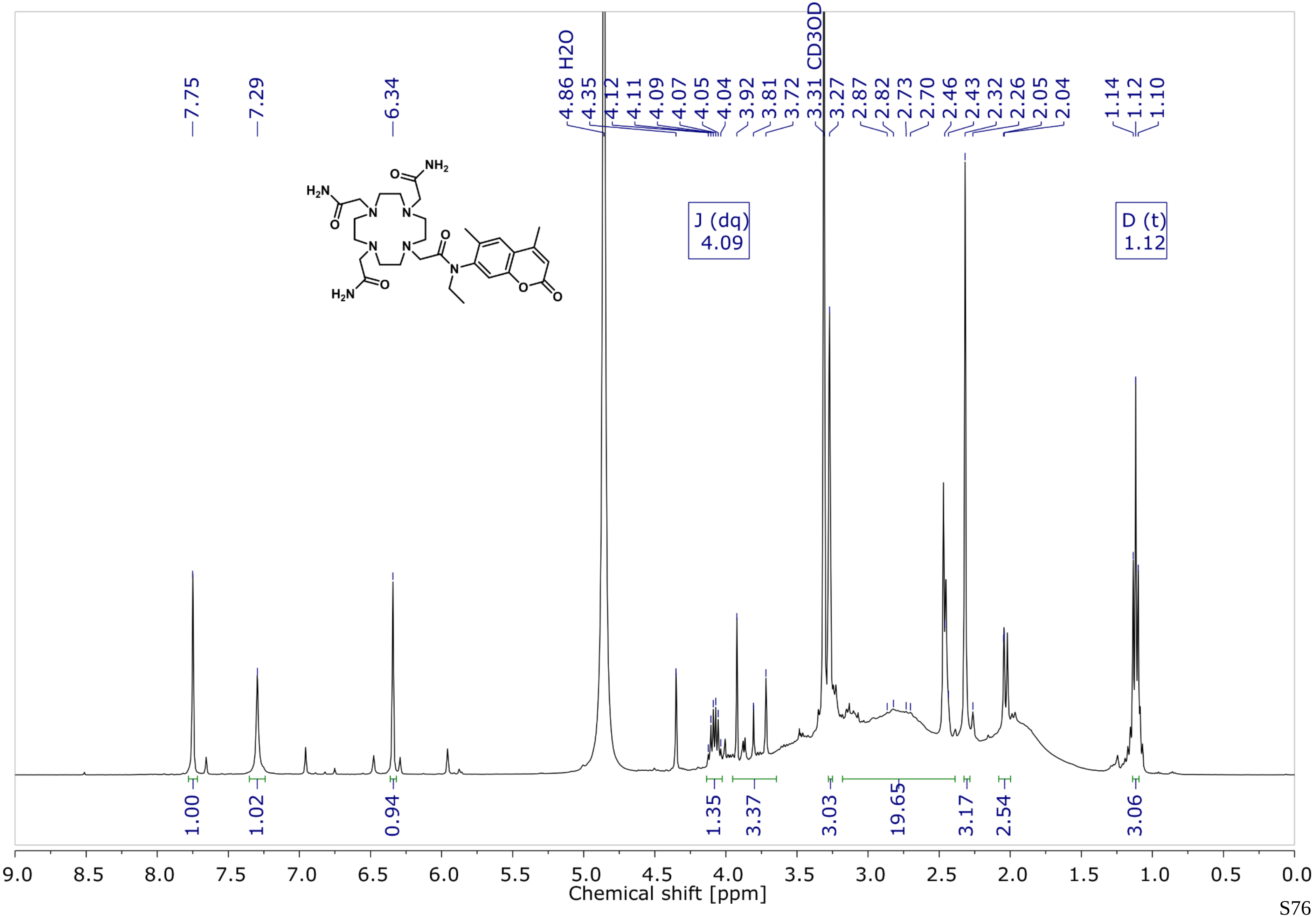

7.75
7.29
6.34
4.86 H2O
4.35
4.12
4.11
4.09
4.07
4.05
4.04
3.92
3.81
3.72
3.31 CD3OD
3.27
2.87
2.82
2.73
2.70
2.46
2.43
2.32
2.26
2.05
2.04
1.14
1.12
1.10
NH2
O
H2N
N
O
H2N
O
N
O
O
J (dq)
4.09
D (t)
1.12
1.00
1.02
0.94
1.35
3.37
3.03
19.65
3.17
2.54
3.06
9.0
8.5
8.0
7.5
7.0
6.5
6.0
5.5
5.0
4.5
4.0
3.5
3.0
2.5
2.0
1.5
1.0
0.5
0.0
Chemical shift [ppm]

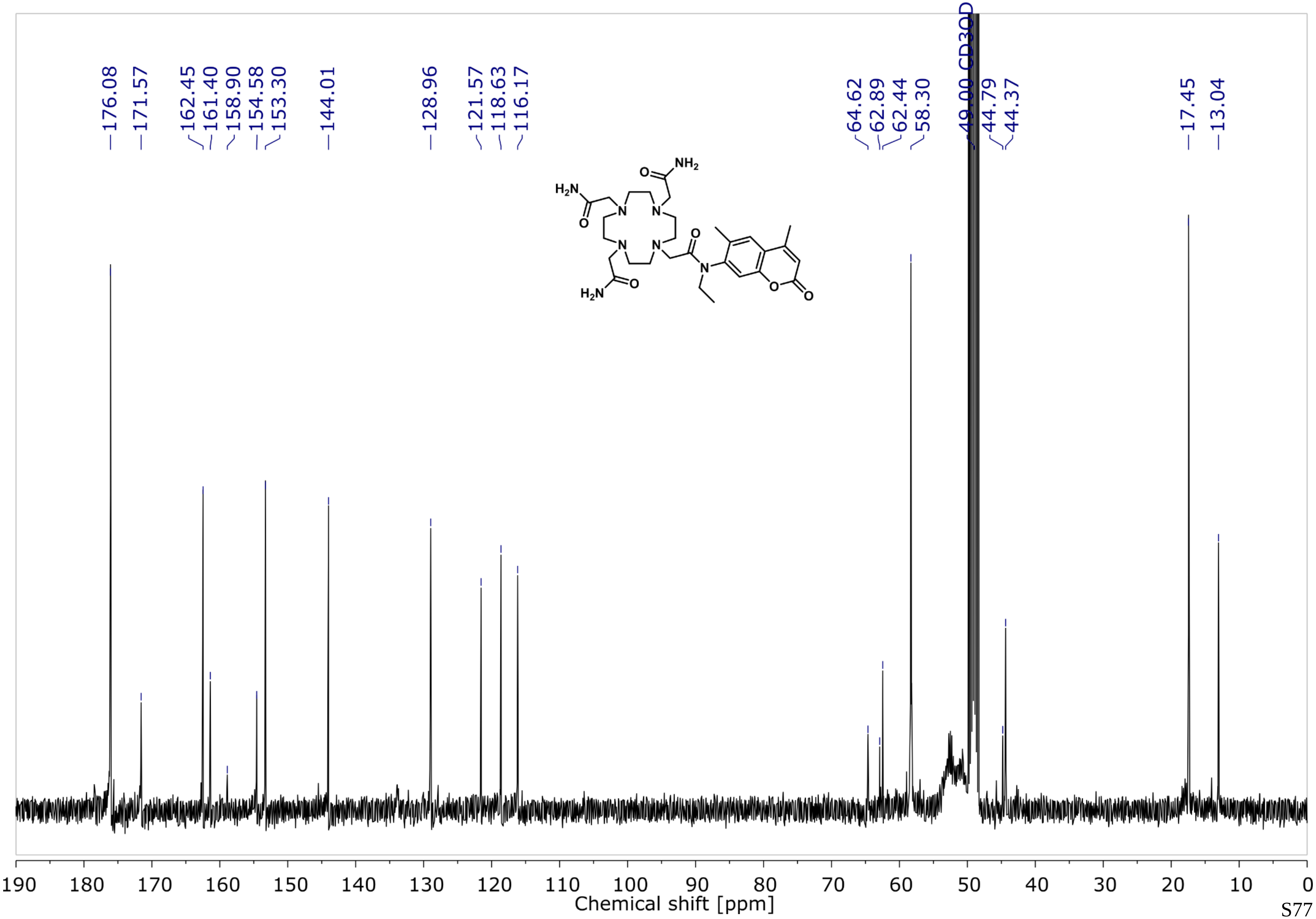
176.08
171.57
162.45
161.40
158.90
154.58
153.30
144.01
128.96
121.57
118.63
116.17
64.62
62.89
62.44
58.30
49.00 CD3OD
44.79
44.37
17.45
13.04
H_2N
NH_2
H_2N
N
O
190 180 170 160 150 140 130 120 110 100 90 80 70 60 50 40 30 20 10 0
Chemical shift [ppm]

References

1. Kovacs, D.; Phipps, D.; Orthaber, A.; Borbas, K. E., Highly luminescent lanthanide complexes sensitized by tertiary amide-linked carbostyril antennae. *Dalton Trans.* **2018,** 47, 10702-10714.
2. Kovacs, D.; Lu, X.; Mészáros, L. S.; Ott, M.; Andres, J.; Borbas, K. E., Photophysics of coumarin and carbostyril-sensitized luminescent lanthanide complexes: Implications for complex design in multiplex detection. *J. Am. Chem. Soc.* **2017,** 139, 5756-5767.
3. Connelly, N. G.; Geiger, W. E., Chemical redox agents for organometallic chemistry. *Chem. Rev.* **1996,** 96, 877-910.
4. Suzuki, K.; Kobayashi, A.; Kaneko, S.; Takehira, K.; Yoshihara, T.; Ishida, H.; Shiina, Y.; Oishi, S.; Tobita, S., Reevaluation of absolute luminescence quantum yields of standard solutions using a spectrometer with an integrating sphere and a back-thinned CCD detector. *Phys. Chem. Chem. Phys.* **2009,** 11, 9850-9860.
5. Harvey, A. H.; Gallagher, J. S.; Sengers, J. M. H. L., Revised formulation for the refractive index of water and steam as a function of wavelength, temperature and density. *J. Phys. Chem. Ref. Data* **1998,** 27, 761-774.
6. Odhner, H.; Jacobs, D. T., Refractive index of liquid D_2O for visible wavelengths. *J. Chem. Eng. Data* **2012,** 57, 166-168.
7. Supkowski, R. M.; Horrocks, W. D., Jr., On the determination of the number of water molecules, q, coordinated to europium(III) ions in solution from luminescence decay lifetimes. *Inorg. Chim. Acta* **2002,** 340, 44-48.
8. Beeby, A.; Clarkson, I. M.; Dickins, R. S.; Faulkner, S.; Parker, D.; Royle, L.; de, S. A. S.; Williams, J. A. G.; Woods, M., Non-radiative deactivation of the excited states of europium, terbium and ytterbium complexes by proximate energy-matched OH, NH and CH oscillators: an improved luminescence method for establishing solution hydration states. *J. Chem. Soc., Perkin Trans. 2* **1999**, 493-504.
9. Kovacs, D.; Mathieu, E.; Kiraev, S. R.; Wells, J. A. L.; Demeyere, E.; Sipos, A.; Borbas, K. E., Coordination environment-controlled photoinduced electron transfer quenching in luminescent europium complexes. *J. Am. Chem. Soc.* **2020,** 142, 13190-13200.
